# Functional Profiling of p53 and RB Cell Cycle Regulatory Proficiency Suggests Mechanism-Driven Molecular Stratification in Endometrial Carcinoma

**DOI:** 10.1158/2767-9764.CRC-24-0028

**Published:** 2025-04-30

**Authors:** Zelei Yang, Saie Mogre, Hyeji Jun, Ruiyang He, Sneha Ghosh Chaudhary, Upendra Raj Bhattarai, Shannan J. Ho Sui, Ursula A. Matulonis, Suzan Lazo, Aniket Shetty, Amy Cameron, Quang-Dé Nguyen, Sarah J. Hill

**Affiliations:** 1 Department of Medical Oncology, Dana-Farber Cancer Institute, Boston, Massachusetts.; 2 Division of Molecular and Cellular Oncology, Dana-Farber Cancer Institute, Boston, Massachusetts.; 3 Harvard Chan Bioinformatics Core, Harvard T.H. Chan School of Public Health, Boston, Massachusetts.; 4 Department of Medicine, Harvard Medical School, Boston, Massachusetts.; 5 Center for Patient-Derived Models, Dana-Farber Cancer Institute, Boston, Massachusetts.; 6 Lurie Family Imaging Center, Dana-Farber Cancer Institute, Boston, Massachusetts.

## Abstract

In the United States, Endometrial carcinoma (EC) is the most frequently occurring gynecologic cancer. Many ECs harbor mutations in cell cycle regulatory genes including *TP53* and *RB1*, amongst others. RB and p53 both regulate the G1/S transition while p53 also regulates the G2/M transition and mitotic progression, all of which rely on targetable regulatory kinases. It is likely that many ECs harbor targetable defects in some aspect of cell cycle regulation, but there has been no profiling of p53- or RB- linked cell cycle functional capacity and corresponding therapeutic vulnerabilities in EC cells. Here, we utilize functional and transcriptomic assays on a panel of EC cell lines and patient-derived organoids to characterize the p53 and RB cell cycle regulatory proficiency and linked therapeutic vulnerabilities in EC. We show that *TP53* genomic and functional status has poor predictive capacity for EC therapeutic response. Rather, proper RB regulation correlates with response to G1/S targeting CDK4/6 inhibitors, and dysfunction in regulation of mitotic progression correlates with response to Aurora kinase B inhibitors. A subset of *TP53* mutant ECs are *RB1* wild type, express RB protein, have intact RB regulation, and are sensitive to CDK4/6 inhibitors, suggesting that excluding patients from emerging CDK4/6 inhibitor trials based on aggressive histology or *TP53* status should be reconsidered. These findings were validated *in vivo* in xenograft models. These results can expand current EC molecular stratification to include mechanism-driven subtypes and suggest clinical trials of novel targeted therapies based on biologic understanding for advanced or recurrent EC patients.

**Significance:**

We show novel cell cycle regulatory molecular classifications and therapeutic targets for endometrial carcinoma. Intact RB regulation and mitotic progression regulatory defects correlate with CDK4/6 and Aurora kinase B inhibitor sensitivity respectively.

## Introduction

Endometrial carcinoma (EC), which arises from epithelial cells in the lining of the uterus, is the most frequently occurring gynecologic malignancy in the United States and is one of the few cancer types continuing to increase in both incidence and mortality (1). ECs are categorized into four histologic subtypes including endometrioid, serous, clear cell, and carcinosarcoma (1). There are limited therapeutic options for advanced or recurrent EC (1).

Genomic profiling across EC histologic subtypes reveals that (i) alterations in *PTEN* and *PIK3CA* are among the most common amidst the limited mutations present across EC subtypes, (ii) mutations in *TP53* and high levels of copy number alterations are common in the serous and high-grade endometrioid tumors, (iii) many tumors have mutations in mismatch repair genes leading to microsatellite instability, and (iv) there can be mutations in the *POLE* gene which lead to tumors with a hyper-mutagenic phenotype (2). *TP53* mutant status has been of particular interest as it correlates with poor clinical outcomes (1, 3, 4). Based on this genomic profiling, ECs are now categorized into four molecular subtypes including *POLE* mutant, mismatch repair deficient/microsatellite unstable, copy number alteration high/p53 abnormal, or copy number alteration low/non-specific molecular profile (NSMP; refs. 1, 2, 5, 6). Since the mismatch repair deficient and copy number high or low classifications have clinically relevant sensitivities to different immune therapy regimens, these molecular features have been incorporated into the new 2023 EC staging (1, 2, 5–7). However, despite the weight placed on these molecular subtypes clinically, how and if any of these genomic alterations leads to true functional defects that can cause EC cells to be sensitive to specific therapies is not fully understood.

A better understanding of the unique biology of the different EC subtypes is needed in order to generate better clinical stratification and more effective targeted therapies for advanced disease. In this regard, there are mutations in many cell cycle regulatory genes in different ECs including but not limited to *TP53*, *PTEN*, *CCNE1*, or *RB1* (2, 8). *TP53* and *RB1* are of particular interest as the proteins they encode regulate cell cycle transitions which might be therapeutically targetable with currently available therapies (9). p53 regulates the G1-S transition and progression from G2 into and through mitosis through multiple mechanisms (8–10). RB primarily regulates the G1-S transition (9, 11). Mutation of *TP53* or *RB1* can lead to deficiencies in regulation of cell cycle progression (8–11). Additionally for RB, although no alterations may be revealed by genomic profiling, its cell cycle regulatory role may be misregulated and not functioning as expected due to alterations in other genes involved in its regulation including but not limited to *CDK4*, *CDK6*, or *CCNE1* (9, 11). It is possible that defects, or lack thereof, in these cell cycle regulatory functions may lead to therapeutic vulnerabilities in EC cells. Therefore, a better understanding of p53 and RB cell cycle regulatory proficiency/deficiency and how this correlates with therapeutic sensitivity in EC is critical since there are now many cell cycle targeted therapies.

Currently existing cell cycle targeted therapies include but are not limited to CDK4/6, Aurora kinase B (AURKB), Aurora kinase A (AURKA), and polo-like kinase-1 (PLK1) inhibitors (12). CDK4/6 inhibitors (CDK4/6i) block phosphorylation of RB in G1 phase, allowing RB to continue to aid in transcriptional repression of cell cycle progression genes; PLK1 inhibitors (PLK1i) block mitotic entry and various aspects of mitotic progression; Aurora kinase A inhibitors (AURKAi) block G2/M progression, centrosome maturation, and bipolar mitotic spindle assembly; and Aurora kinase B inhibitors (AURKBi) block proper kinetochore-microtubule attachment, chromosome alignment, and separation of sister chromatids during mitosis, as well as, cytokinesis (12–14). CDK4/6is have recently been tested in EC, with some promising results, but no clear biomarkers for response (15–18). An AURKBi, an AURKA/B inhibitor (19–21), and several pan-Aurora kinase inhibitors have been tested across cancer types, including some showing activity in ovarian cancer, but have not been tested in EC and have no clear biomarker for response (22–30). PLK1 inhibitors and pan-PLK inhibitors have undergone clinical investigation, and although showing some activity, have often been associated with significant toxicity (31, 32).

Given the potential for cell cycle progression alterations in EC and the many cell cycle targeted therapies available, we sought here to assess the functional capacity of p53 and RB in regulating the cell cycle across different EC histologies and genotypes and to determine how regulatory proficiencies and deficiencies correspond to response to currently available cell cycle targeted therapies. We show that *TP53* genomic status has no predictive capacity for therapeutic response to cell cycle therapies. Rather, RB is expressed, properly regulated, and functioning as expected in many EC cells regardless of histologic subtype or *TP53* mutational status. Intact RB regulation in *RB1* wild type (WT) EC cells expressing RB protein, as indicated by functional and transcriptomic profiling, correlates with CDK4/6i sensitivity. Additionally, many EC cells reveal both functional and transcriptomic evidence of defects in regulating mitotic progression, in particular weakened activation of the spindle assembly checkpoint or an inability to maintain a spindle assembly checkpoint induced mitotic arrest, which correlates with sensitivity to an AURKBi. These results were validated *in vivo*. Taken together, these results indicate that subtyping ECs by *TP53* mutational status or some of the other current molecular subtypes may be insufficient. Rather, they (i) suggest that further mechanism-driven molecular stratification of ECs based on RB regulation status and mitotic progression regulatory proficiency may be a relevant therapeutic strategy, and (ii) offer two new therapies for advanced or recurrent EC patients.

## Materials and Methods

Please also see Supplementary Materials and Methods.

### Human tissue samples

Endometrial tumor samples for organoid generation were obtained from four patients undergoing surgery or having ascites drained at Brigham and Women’s Hospital or Dana-Farber Cancer Institute (DFCI, Boston, MA; Supplementary Table S1). Written informed consent was obtained for all four patients on DFCI IRB-approved protocol 02-051. The human subjects work in this manuscript was approved by the DFCI IRB and conducted in accordance with the Belmont Report and U.S. Common Rule.

### Cell lines

HEC1B (ATCC Cat. # HTB-113, RRID: CVCL_0294), AN3CA (ATCC Cat. # HTB-111, RRID: CVCL_0028), RL95-2 (ATCC Cat. # CRL-1671, RRID: CVCL_0505), and KLE (ATCC Cat. # CRL-1622, RRID: CVCL_1329) cells were purchased from ATCC. Ishikawa cells were purchased from Sigma-Aldrich (Cat. #99040201-1VL). ARK1 (RRID: CVCL_IV72) and ARK2 (RRID: CVCL_IV73) cells were obtained from Dr. Alessandro Santin at Yale University. HEC1B, AN3CA, Ishikawa, RL95-2, and KLE cells were validated by short tandem repeat (STR) profiling in the Center for Patient-Derived Models at Dana-Farber Cancer Institute. ARK1 and ARK2 cells also underwent STR profiling. The STR profiles for ARK1 and ARK2 cells have not been previously published, but the STR profiles we obtained for these models were unique from each other and all other cell lines. Additionally, we performed whole exome sequencing on ARK1 and ARK2 cells, as described in Supplementary Materials and Methods, and the same previously detected *TP53* mutations in ARK1 and ARK2 cells were detected in our analysis (Supplementary Fig. S1A and S1B; refs. 33, 34). All cell lines were confirmed to be negative for mycoplasma by PCR, and all were utilized for experiments at early passage. ARK1 and ARK2 cells were grown in Roswell Park Memorial Institute (RPMI) 1640 (Gibco Cat. #11875-093), 10% FBS (Sigma-Aldrich Cat. #F2442), and 1% penicillin/streptomycin (P/S) (Gibco Cat. #15140-122). HEC1B and AN3CA cells were grown in Minimum Essential Medium (MEM, Corning Cat. #10-010-CV), 10% FBS, and 1% P/S. KLE cells were grown in Dulbecco’s Modified Eagle Medium (DMEM)/F-12 1:1 (Gibco Cat. # 11320-033), 10% FBS, and 1% P/S. Ishikawa cells were grown in MEM, 5% FBS, 1% L-Glutamine (Gibco Cat. # 25030-081), 1% MEM Non-Essential Amino Acids (Gibco Cat. # 11140-050), and 1% P/S. RL95-2 cells were grown in DMEM/F-12 1:1, 10% FBS, 1% P/S, and 1X Insulin-Transferrin-Selenium (Gibco Cat. # 41400045). All cell lines were grown at 37°C in 5% CO_2_.

### Abemaciclib used in the study

Abemaciclib methanesulfonate (referred to as “Abemaciclib” in the main text and Supplementary Materials and Methods) was provided by Eli Lilly and Company, and this was utilized for all *in vitro* and *in vivo* experiments in which Abemaciclib was used, described here and in the Supplementary Materials and Methods.

### Bromodeoxyuridine cell cycle flow cytometry for cell lines

These methods have been described previously, and an updated version of the methods specific to this work is provided below (35). Cell lines were plated and then treated with different drugs for different studies. Treatments were as follows: (i) cells were incubated in media containing either 0.25 µM Abemaciclib or media containing an equivalent volume of Dimethyl sulfoxide (DMSO) vehicle (ATCC Cat. #4-X-5) for 24 hours, (ii) cells were incubated in media containing either 0.1 µM Barasertib (MedChemExpress Cat. # HY-10127), Alisertib (MedChemExpress Cat. # HY-10971), MK5108 (MedChemExpress Cat. # HY-13252), Onvansertib (MedChemExpress Cat. # HY-15828) or an equivalent volume of DMSO for 24 hours, (iii) cells were incubated in media containing 0.25, 1, 2.5, 5, or 9 µM Ro-3306 (MedChemExpress Cat. #HY-12529) or a volume of DMSO equivalent to the highest Ro-3306 dose for 16 hours, (iv) cells were incubated in media containing 0.05, 0.1, 0.25, 0.5, or 1 µM Barasertib or a volume of DMSO equivalent to the highest Barasertib dose for 24 hours, (v) cells were incubated for 24 hours in media containing either 0.25 µM Palbociclib (MedChemExpress Cat. # HY-50767) or media containing an equivalent volume of DMSO, (vi) cells were incubated in media containing 5, 10, 15, 20, or 25 ng/mL nocodazole (Sigma-Aldrich Cat. #SML1665) or a volume of DMSO equivalent to the highest nocodazole dose for 24 hours, or (vii) cells were first treated with media containing 9 µM Ro-3306 or media containing an equivalent volume of DMSO for 16 hours, washed five times with pre-warmed media, and then treated with media containing either 0.1 µM Barasertib, 10 ng/mL nocodazole, 20 ng/mL nocodazole, or an equivalent amount of DMSO for 24 hours. One hour prior to harvest for the respective treatments, bromodeoxyuridine (BrdU; BioLegend Cat. # 423401) was added to the media to a final concentration of approximately 10 µM. After one hour of BrdU incubation, cells were trypsinized and neutralized with serum containing media, pelleted, and washed in PBS. One mL of cold (−20°C) 70% ethanol was added to the pellets, and the pellets were stored at −20°C for a minimum of 20 minutes and up to two weeks before processing. Cells were spun to pellet the cells, ethanol was aspirated, and cells were washed once in PBS-Tween 20 (PBS-T). Cells were pelleted, and 500 µL of fresh 2N HCl diluted in ddH_2_O was added for 15 minutes at room temperature. Cells were then immediately washed in PBS-T. Cells were pelleted, and 1 mL of 0.1 M Na_2_B_4_O_7_ pH8.5 in 1% BSA was added for 30 minutes at room temperature. Cells were pelleted and 50 µL of FITC conjugated anti-BrdU antibody (BD Cat. #556028, RRID: AB_396304) at 1:10 in 1% BSA were added for 30 minutes at room temperature in the dark. Cells were washed in PBS-T and pelleted. Cells were incubated in propidium iodide RNASE staining buffer (BD Cat. #550825; RRID: AB_2868904) for a minimum of 30 minutes or until being analyzed on a BD LSR Fortessa flow cytometer. For analysis, equal numbers of cells were gated for each line for each treatment in FlowJo analysis software. All experiments were repeated two to three times.

### 5-ethynyl-2′-deoxyuridine cell cycle flow cytometry for organoids

Organoid lines were split to single cells and allowed to recover for approximately five days. Please see Supplementary Materials and Methods for detailed organoid culture methods. Media was then changed to media containing either (i) 0.25 µM Abemaciclib or an equivalent volume of DMSO, or (ii) 0.1 µM Barasertib or an equivalent volume of DMSO for 24 hours. At the 24 hour timepoint, 5-ethynyl-2′-deoxyuridine (EdU; Cayman Chemicals Cat. # 20518) was added to the treated organoids to a final concentration of 10 µM for an additional 16 hours. This made the drug treatments 40 hours and the EdU treatment the last 16 hours of the drug treatment. At the 16 hour timepoint, organoids were scraped from the plate, and digested in TrypLE (Gibco Cat. #12604-013) for 20 minutes at 37°C with shaking to get to single cells. Organoids were pelleted, washed in Cell Staining Buffer (BioLegend Cat. #420201), and then fixed in 4% paraformaldehyde (Electron Microscopy Sciences Cat. #15710-S diluted to 4% in PBS) for 15 to 20 minutes at room temperature. The single cell suspensions were washed in Cell Staining Buffer and then incubated in BioLegend’s Permeabilization/Wash buffer (BioLegend Cat. #421002) at room temperature for 20 minutes. Cells were washed in Cell Staining Buffer and then incubated in the following staining reaction mixture for 30 minutes at room temperature in the dark (Staining Reaction Mixture: 1 mM CuSO_4_, 0.1 mM THPTA, 2 µM AZDye 647 Azide Plus (Click Chemistry Tools Cat. #1482-1), and 100 mM sodium ascorbate mixed in PBS). Cells were washed in Cell Staining Buffer, and then incubated in propidium iodide/RNASE staining buffer (BD Cat. #550825) until being analyzed on a BD LSR Fortessa flow cytometer. For analysis, equal numbers of cells were gated for each line for each treatment in FlowJo analysis software. All experiments were repeated three times.

### CDK1 inhibitor/Barasertib and CDK1 inhibitor/Nocodazole flow cytometry analysis

ARK1, AN3CA, or HEC1B cells were plated in 6 cm plates on day one. The next day, the media was changed to media containing either 9 µM of the CDK1 inhibitor Ro-3306 or media containing an equivalent volume of DMSO, and the cells were incubated in this media for 16 hours. At the 16 hour timepoint, all cells were washed five times with 1 mL of pre-warmed media, and media containing either (i) 0.1 µM of the Aurora kinase B inhibitor Barasertib or an equivalent volume of DMSO, (ii) 10 ng/mL nocodazole or an equivalent volume of DMSO, or (iii) 20 ng/mL nocodazole or an equivalent volume of DMSO was added. 45 minutes, 4 hours, or 24 hours later, the cells were trypsinized and neutralized, washed once with Cell Staining Buffer (BioLegend Cat. #420201), and then stained.

Cells were incubated in Cell Staining buffer containing Zombie NIR viability dye (BioLegend Cat. #423105) at 1:200 for 20 minutes at room temperature in the dark. The cells were then washed in Cell Staining Buffer, and were then incubated in Fixation Buffer (BioLegend Cat. #420801) for 20 minutes at room temperature in the dark. If the cells were from a 45 minute or 4 hour timepoint, the cells were washed in Cell Staining Buffer and then stored in Cell Staining Buffer at 4°C in the dark until the 24 hour timepoint for the experiment was collected. Once all timepoints for an individual experiment were collected, stained with viability dye, fixed, and washed, the cells were then washed once in Permeabilization Wash Buffer (BioLegend Cat. #421002), and then were incubated in Permeabilization Wash Buffer containing unconjugated MPM2 antibody (Abcam Cat. # ab14581; RRID: AB_301354) at 1:500 for 20 minutes at room temperature in the dark. The cells were washed one time in Permeabilization Wash Buffer, and were then incubated in Permeabilization Wash Buffer containing FITC anti-mouse IgG (BioLegend Cat. #406001; RRID: AB_315029) at 1:250 for 20 minutes at room temperature in the dark. The cells were washed twice in Permeabilization Wash Buffer, and were then incubated in Permeabilization Wash Buffer containing PE conjugated anti-histone H3 phosphorylated on serine 10 antibody (BioLegend Cat. # 650807; RRID: AB_2564562) at 1:200 for 20 minutes at room temperature in the dark. The cells were washed in Cell Staining Buffer, and then stored in Cell Staining Buffer until being analyzed on a flow cytometer. Cells were analyzed on a BD LSR Fortessa Flow Cytometer. Cells were gated in a standardized way across treatments in FlowJo analysis software. The experiment was repeated three times.

An initial Fluorescence Minus One (FMO) antibody/dye validation study was performed on untreated HEC1B cells with the above dye/antibodies using the overall staining procedure described above, but with some modifications for each FMO sample. For this validation study, a single dye or antibody was left out of an FMO sample when it was being stained, as appropriate for that specific FMO sample (i.e., during just this validation experiment for the NIR FMO sample only, NIR viability dye was left out during that staining step for that sample and then all the other staining steps were performed with antibody as detailed above for that NIR FMO sample). Staining and analysis parameters for FMO study samples are indicated in the respective Figure and Figure legend when relevant.

### Animal studies

All animal studies were conducted at the Lurie Family Imaging Center at Dana-Farber Cancer Institute (DFCI) under the DFCI IACUC approved protocol #08-023. ARK1 and HEC1B cells were luciferized using AMSBIOs viral particles (Cat. # LVP433). Both cell lines underwent murine pathogen testing at Charles River Laboratories and were negative for all murine pathogens tested. For the HEC1B model study, 20 six to eight week old female ovariectomized Fox Chase SCID mice (RRID: IMSR_CRL:236) were obtained from Charles River Laboratories and implanted subcutaneously with slow release estrogen pellets (0.18 mg/pellet 90-days release, Innovative Research of America Cat. #NE-121). Mice were allowed to recover, and then all 20 animals underwent intraperitoneal injection of 1 × 10^6^ luciferized HEC1B cells. For the ARK1 model study, 20 six week old female Fox Chase SCID mice were obtained from Charles River Laboratories. The mice were allowed to recover, and then each mouse underwent intraperitoneal injection of 1 × 10^6^ luciferized ARK1 cells. Animals were monitored daily for any signs of morbidity, were monitored for changes in weight, and underwent at least weekly bioluminescent imaging (BLI) to monitor tumor formation, as described in Supplementary Materials and Methods. When the average cohort BLI signal reached a threshold of 1.5 × 10^6^ for the HEC1B model or 6.1 × 10^^5^^ for the ARK1 model, animals were randomized into treatment groups and treatments were initiated as follows. The HEC1B tumor-bearing mice were dosed daily via oral gavage with either vehicle or Abemaciclib as part of a combination study. The Abemaciclib treated mice were treated daily via oral gavage with 50 mg/kg Abemaciclib methanesulfonate provided by Eli Lilly and Company prepared in 10% DMSO to 90% 25 mM phosphate buffer pH = 2.0 (phosphate buffer generated by mixing 3.549 g Na_2_HPO_4_ (Sigma-Aldrich Cat. #567547) and 3.40 g KH_2_PO_4_ (Sigma-Aldrich Cat. #P5655) in one L of ddH_2_O, adjusted to pH 2.0 with hydrochloric acid prior to autoclaving) with 1% hydroxyethyl cellulose (HEC; Sigma-Aldrich Cat. #09368). The HEC1B model vehicle treated mice were dosed daily via oral gavage with the Abemaciclib vehicle (10% DMSO to 90% 25 mM phosphate buffer pH = 2.0 with 1% HEC) and a second vehicle [1% HEC with 0.25% Tween 80 (Sigma-Aldrich Cat. # 59924-100G-F)]. The ARK1 tumor-bearing mice were dosed daily four days per week by intraperitoneal injection with either vehicle (30 mM Tris buffer pH 9.0) or 25 mg/kg Barasertib (MedChemExpress Cat. #HY-10127). Once animals developed signs of morbidity (including 15% loss of body weight from maximum weight, poor body condition (BCS 2), and/or highly distended abdomen) they were euthanized via CO_2_ asphyxiation. At that point, ascites volume was obtained, gross photos were taken, and residual tumor was harvested for all animals possible. Of note, one animal in the Abemaciclib group was found dead in the cage 11 days after treatment initiation. The death was not considered to be related to experimental procedures, and the animal was included in the survival analysis. However, ascites and residual tumor were not able to be recovered from this animal.

### Photographic images

For images of organoids, photos of animals, or photos of hematoxylin and eosin- or immunohistochemistry- stained sections on slides, photos were taken using optimized settings on a camera (animals) or microscope with attached camera (organoids and stained sections on slides) for each individual specimen or sample as those specimens/samples became available.

### Statistics

All cell and organoid line experiments were performed in duplicate or in triplicate. All statistical analyses for non-sequencing experiments were performed using GraphPad Prism software. *P*-values were generated using either (i) a paired or unpaired t-test, or (ii) an ordinary one-way ANOVA or an ordinary two-way ANOVA with post-hoc multiple comparisons test, all as indicated in respective Figure legends. A log-rank test was performed to compare Kaplan-Meier survival curves for the mouse model studies. Statistical analyses for sequencing data were performed as indicated in the corresponding Figure legends and Supplementary Materials and Methods.

### Cartoon generation

**Figure 1:**
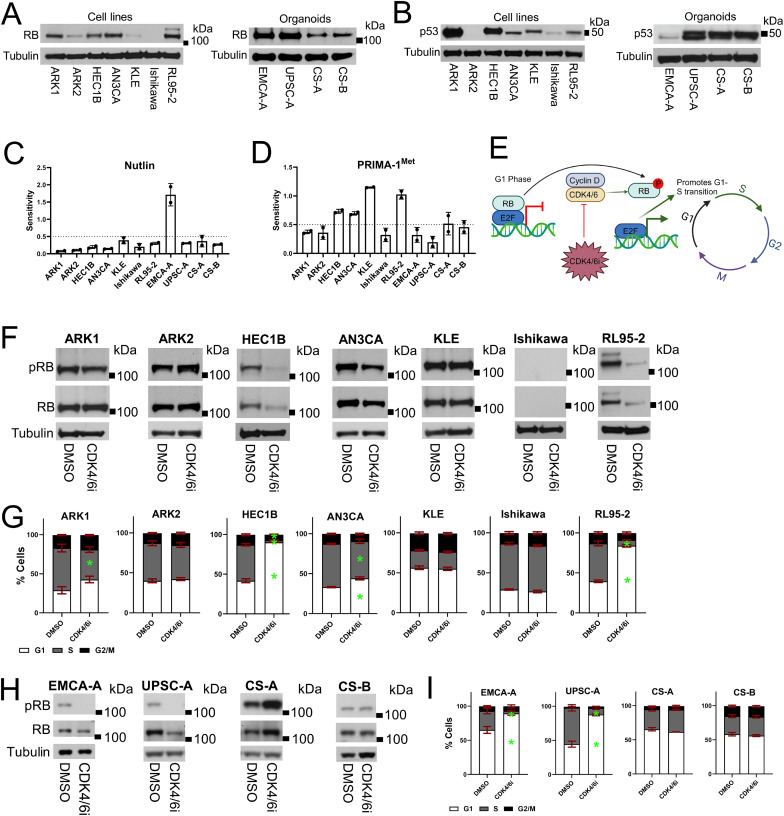
RB and some form of p53 are expressed in the majority of endometrial carcinoma cells regardless of genomic status but have varying regulatory status or functional ability. **A** and **B,** Protein lysates were prepared from untreated cell lines (left in each panel) and organoid lines (right in each panel) and analyzed by western blot. In **A**, membranes for each cell type were probed for RB, and stripped and re-probed for tubulin as a loading control. In B, membranes for each cell type were probed for p53, and then stripped and re-probed for tubulin as a loading control. **C** and **D,** Organoids and cell lines were treated with a dose curve of Nutlin-3 (Nutlin) in **C** or the methylated derivative of P53-dependent reactivation and induction of massive apoptosis-1 (PRIMA-1^Met^) in **D**, and survival was assessed after five days by CellTiter-Glo. The day five CellTiter-Glo reads along with initial CellTiter-Glo reads of untreated cells taken on the day the cells were plated and treatment initiated were then used to generate a growth rate corrected dose-response curve for each model with each agent to compensate for the varying cell cycle rates of the different models. The area over the growth rate corrected dose-response curve (AOC) was then calculated for each model with each agent. The AOC represents the sensitivity of the model to the agent (larger AOC = greater sensitivity). For additional details, please see Supplementary Materials and Methods. The experiment was repeated twice for each model with each agent. The bar graphs show the average sensitivity for each model to each agent with error bars representing standard deviation. A dashed line at 0.5 on each graph denotes an arbitrary cutoff for sensitivity of a cell or organoid line, and all models with an average sensitivity of 0.5 or above were deemed sensitive to the agent tested. **E,** Cartoon demonstrating the effects of CDK4/6 inhibitors (CDK4/6i) on RB-mediated progression from G1 into S phase. **F–I,** Cell line and organoid models were treated with either 0.25 µM of the CDK4/6 inhibitor (CDK4/6i) Abemaciclib or vehicle (DMSO) for 24 hours and then analyzed. In **F** and **H**, protein lysates were prepared from treated cells and analyzed by western blot. Membranes were first probed for RB phosphorylated on serine 807 and 811 (pRB), stripped and re-probed for RB, and lastly stripped and re-probed for tubulin as a loading control. In **G** and **I**, treated cell lines or organoids underwent bromodeoxyuridine/propidium iodide (PI) or 5-Ethynyl-2′-deoxyuridine/PI cell cycle flow cytometry profiling respectively. For the bar graphs in **G** and **I**, bars represent the percent of cells in each different cell cycle phase from three independent replicates, and error bars represent standard error of the mean. * = *P* < 0.05 compared to DMSO for the specific cell cycle phase by an ordinary two-way ANOVA with Šídák’s multiple comparisons test. If there is no *, then the comparison was not significant. The color code for the cell cycle phase is below the first graph in the panel. Please see Supplementary Fig. S4A–S4C for representative gating strategies of one of the replicates for the flow cytometry data in **G** and **I**.

Cartoons in Figs. 1E, 3A, 4A, 5A, and 6A were generated using BioRender.com. Publication licenses for all BioRender cartoons are available upon request.

### Data availability

All RNA sequencing and whole exome sequencing data can be found at GEO accession number GSE247793. All other data are available upon request from the corresponding author.

## Results

### EC cell lines and patient-derived organoids have variable RB and p53 expression

To study the influence of *TP53* and *RB1* genomic or functional alterations on cell cycle regulatory proficiency and deficiency and subsequent therapeutic response, we compiled a panel of EC cell lines and patient-derived organoids (PDOs) with differing *TP53* and *RB1* genomic status. These included the EC cell lines HEC1B, AN3CA, Ishikawa, RL95-2, and KLE; the uterine papillary serous carcinoma cell lines ARK1 and ARK2; and four PDOs generated during this study (Table 1; Supplementary Table S1; refs. 36–42). PDOs were generated from parent tumors of endometrioid carcinoma (EMCA), uterine papillary serous carcinoma (UPSC), and carcinosarcoma (CS) EC subtypes (Supplementary Table S1). ARK1, ARK2, and all four PDO lines underwent limited whole exome sequencing analysis to determine *TP53*, *RB1*, and other relevant gene mutation status (Supplementary Fig. S1A and S1B; Table 1). Publicly available data was utilized to obtain *TP53* and *RB1* mutation status for the other cell lines (Table 1; ref. 39). All cell and organoid lines, except for *TP53* WT EMCA-A, harbor primarily missense or nonsense mutations in *TP53* (Supplementary Fig. S1B; ref. 39). Additionally, all cell lines and organoids underwent immunohistochemical (IHC) analysis for p53 since p53 IHC is often used to mark *TP53* mutation status (Supplementary Fig. S1C and S1D; Table 1; refs. 1, 4). Overall, *TP53* genomic status matched with p53 IHC, with *TP53* WT cells showing heterogeneous nuclear staining and *TP53* mutant cells revealing null staining or mostly strong diffuse nuclear staining, as expected (Table 1; ref. 4).

**Table 1:** Immunohistochemical, genomic, functional/regulatory, and related therapeutic sensitivity status of p53 and RB in endometrial carcinoma models

Organoid/ cell line	p53 IHC	*TP53* gene status	Nutlin response	PRIMA-1^Met^ response	*RB1* gene status	RB regulatory status	CDK4/6 inhibitor response	Aurora Kinase B inhibitor response
ARK1	Strong diffuse nuclear staining	Mutant	Resistant	Resistant	No mutation detected	Misregulated	Resistant	Sensitive
ARK2	Null	Mutant	Resistant	Resistant	No mutation detected	Misregulated	Resistant	Sensitive
HEC1B	Strong diffuse nuclear staining	Mutant	Resistant	Sensitive	No reported mutation	Intact regulation	Sensitive	Resistant
AN3CA	Strong diffuse nuclear staining with scattered negative nuclei	Mutant	Resistant	Sensitive	No reported mutation	Misregulated	Resistant	Sensitive
KLE	Strong diffuse nuclear staining with scattered negative nuclei	Mutant	Resistant	Sensitive	No reported mutation	Misregulated	Resistant	Resistant
Ishikawa	Strong diffuse nuclear staining with scattered negative nuclei	Mutant	Resistant	Resistant	Mutant	RB protein not expressed	Resistant	Sensitive
RL95-2	Strong diffuse nuclear staining with scattered negative nuclei	Mutant	Resistant	Sensitive	No reported mutation	Intact regulation	Sensitive	Sensitive
EMCA-A	Heterogeneous	Wild type	Sensitive	Resistant	No mutation detected	Intact regulation	Sensitive	Sensitive
UPSC-A	Strong diffuse nuclear staining with scattered negative nuclei	Mutant	Resistant	Resistant	No mutation detected	Intact regulation	Sensitive	Resistant
CS-A	Strong diffuse nuclear staining with scattered negative nuclei	Mutant	Resistant	Sensitive	No mutation detected	Misregulated	Resistant	Sensitive
CS-B	Strong diffuse nuclear staining with scattered negative nuclei	Mutant	Resistant	Resistant	No mutation detected	Misregulated	Resistant	Sensitive

Summary of the p53 immunohistochemical (IHC) status, genomic status, p53 functional status, and RB regulatory status in the 11 model panel along with linked sensitivity to Nutlin-3 (Nutlin), PRIMA-1^Met^, or a CDK4/6 or Aurora kinase B inhibitor. For *TP53* and *RB1* mutational status, ARK1, ARK2, EMCA-A, UPSC-A, CS-A, and CS-B underwent whole exome sequencing in this study, and HEC1B, AN3CA, Ishikawa, RL95-2, and KLE were previously analyzed by the Cancer Cell Line Encyclopedia. Models are reported as *TP53* or *RB1* mutant if a mutation was detected by one of the above analyses. Copy number analysis is not included here as data was not available for all models. For p53 IHC, we focused on nuclear staining. Staining patterns included heterogeneous for a mix of cells with p53 nuclear staining of varying strength or no nuclear staining, null for no nuclear staining, strong diffuse nuclear staining for models in which almost all nuclei had strong nuclear staining, and strong diffuse nuclear staining with scattered negative nuclei for models that had predominantly strong nuclear staining in most cells but also rare scattered negative cells on the slide.

Abbreviations: CS, carcinosarcoma; EMCA, endometrioid carcinoma; UPSC, uterine papillary serous carcinoma.

We next assessed for expression of RB and p53 by western blot. Only one model revealed a mutation in *RB1*, Ishikawa, and all models except Ishikawa revealed RB expression at varying levels by western blot analysis with an siRNA validated antibody (Fig. 1A; Table 1; Supplementary Figs. S1A, S2A; ref. 39). We also tested for expression of p53, given that 10 of the 11 models being utilized revealed *TP53* mutations, and IHC showed at least one null staining pattern (Table 1). All models except for ARK2 revealed expression of some form of p53 protein by western blot with an siRNA validated antibody, with varying expression levels (Fig. 1B; Supplementary Fig. S2B). Others have shown that overexpressed mutant p53 may have a role in tumor cells. We tested this possibility in the two cell lines with strong mutant p53 expression, ARK1 and HEC1B, and determined that depletion of mutant p53 in these two lines leads to reduced colony formation (Supplementary Fig. S2C).

Taken together, these results suggest that RB and some form of p53 are expressed in almost all models, that p53 IHC matches *TP53* genomic status in all models, and that mutant p53 is important for the survival of at least some EC cells in which it is strongly expressed. Our next question was whether RB, despite lacking genomic alterations, and/or mutant p53 are functional in EC cells.

### RB and mutant p53 have varying functional capacity in EC cells

We thus utilized functional assays to determine if RB or mutant p53 have functional roles regardless of their mutational status or that of other related genes. To test p53 function, we assessed for sensitivity of the models to two small molecules which affect WT and mutant p53 protein differently. We tested for sensitivity to the MDM2 inhibitor Nutlin-3 (Nutlin) which releases p53 from control of its regulatory partner MDM2 thereby activating p53 and to which cells which harbor WT p53 are sensitive (43, 44). We tested for sensitivity to the methylated derivative of P53-dependent reactivation and induction of massive apoptosis-1 (PRIMA-1^Met^), which promotes proper folding and restores some function of mutant p53 leading to apoptosis in at least some cells harboring mutant p53 (44). Only the PDO with WT *TP53*, EMCA-A, was highly sensitive to Nutlin (Fig. 1C; Table 1; ref. 35). HEC1B, AN3CA, KLE, RL95-2, and CS-A, which are all *TP53* mutant but still express varying amounts of some form of p53 protein, exhibited varying sensitivity to PRIMA-1^Met^ (Fig. 1B and D; Table 1; ref. 35). The variable PRIMA-1^Met^ responses among *TP53* mutant cells are not unexpected as the background functional and genomic context of different tumor cells can potentially alter the gain of function abilities of mutant p53, causing even the same or similar mutant p53 proteins to have different abilities in different tumor cells (8, 45).

Despite the fact that all models except Ishikawa cells were *RB1* WT and expressed RB protein, we hypothesized that some of the models could have misregulation of the expressed RB protein due to alterations in RB regulatory proteins which might prevent RB in those models from functioning normally (Table 1). Thus, we tested if the RB protein expressed in most models was functioning as expected. To do this, we treated all models with a CDK4/6i, which requires intact RB signaling and thus proper RB regulation to exert its effects, and then performed functional assays to determine if our EC cells exhibited the expected RB-linked responses to this agent (9, 11). A lack of expected response in *RB1* WT, RB protein expressing cells might suggest that RB is misregulated in the model. Normally, during G1 phase RB is hypo-phosphorylated and binds to the E2F transcription factor family causing inhibition of transcription of E2F target genes such as *RRM2*, amongst others (9, 11, 46). To promote the transition from G1 to S phase, a Cyclin D-CDK4/6 and/or a Cyclin E/CDK2 complex phosphorylates RB, phosphorylated RB and E2F dissociate from each other, and E2F then promotes transcription of genes required for S phase entry (Fig. 1E; refs. 9, 11). CDK4/6is block this progression by preventing RB phosphorylation (Fig. 1E). If RB is properly regulated in an EC cell, meaning it is not mutated or deleted and there are not functional alterations in RB regulatory proteins which may also alter RB-E2F signaling, then upon CDK4/6i treatment we expect to observe the following: (i) reduced RB phosphorylation signifying response to the drug; (ii) reduced inactivating phosphorylation of CDC2 (also known as CDK1) on Tyrosine 15, a reduction of which is known to occur in G1 phase and also at the G2/M transition and signifies lack of cell cycle progression; (iii) reduced expression of the E2F target RRM2 signifying continued RB-E2F transcriptional repression of cell cycle progression; and (iv) arrest of cells in G1 phase of the cell cycle manifesting the functional ability of RB to control the G1-S transition (46–49). Thus, we treated our EC cells with an optimized and specific low dose of the CDK4/6i Abemaciclib and assessed for the above responses. We equated expected responses to CDK4/6i treatment with intact RB regulation. Upon CDK4/6i treatment, the *TP53* WT PDO EMCA-A, as well as the *TP53* mutant cell lines and PDO HEC1B, RL95-2, and UPSC-A revealed reduced RB and CDC2 phosphorylation, reduced RRM2 expression, and at least a 15% increase in G1 phase cells (Fig. 1F–I; Supplementary Fig. S2D for RRM2 antibody validation; Supplementary Fig. S3A–S3D, and representative flow cytometry gating strategy in Supplementary Fig. S4A– S4C). This suggests that RB is properly regulated in EMCA-A, UPSC-A, HEC1B, and RL95-2 cells (Table 1). As expected, Ishikawa cells, which harbor an *RB1* mutation and do not express RB protein, revealed little or no reduction in CDC2 phosphorylation or RRM2 expression, and did not show a greater than 15% increase in the percentage of G1 phase cells upon CDK4/6i treatment (Fig. 1A, F, and G; Supplementary Fig. S3A and S3B; Table 1). Although they all express RB protein and did not reveal *RB1* mutations, ARK1, ARK2, AN3CA, KLE, CS-A, and CS-B cells revealed little or no reduction in RB or CDC2 phosphorylation or RRM2 expression, and did not show a greater than 15% increase in the percentage of G1 phase cells upon CDK4/6i treatment, suggesting that RB is likely misregulated in these models and unable to respond to a CDK4/6i as a result (Fig. 1A and 1F–I; Supplementary Fig. S3; Table 1). To be certain these results were not specific to Abemaciclib, we repeated some of the western blot and flow cytometry analysis post-Palbociclib, another CDK4/6i, in the cell line models, and observed similar results as with Abemaciclib (Supplementary Fig. S5A–S5C).

Taken together, the above results suggest that mutant p53 may have residual function in some EC cells and that regardless of *TP53* genomic status, RB regulation can remain intact in EC cells. Our next question was how the different functional ability of mutant p53 or regulatory status of RB affect sensitivity to cell cycle targeted therapies.

### RB regulatory status correlates with sensitivity to CDK4/6 inhibition

We expected that *RB1* WT, RB protein expressing EC cells able to demonstrate appropriate cell cycle arrest and E2F target downregulation in our functional assays in response to a CDK4/6i, which indicates intact RB regulation, should be highly sensitive to CDK4/6is. Any lack of CDK4/6i sensitivity or functional assay response in *RB1* WT, RB protein expressing EC cells might be attributed to genomic or functional alterations in other pathways that cause misregulation of RB-E2F signaling. Therefore, we tested our EC models with varying p53 functional and RB regulatory status (i) for known mechanisms of resistance to CDK4/6 inhibition which cause RB misregulation including upregulation of CDK4, CDK6, or Cyclin E, and (ii) for CDK4/6i sensitivity (11).

**Figure 2:**
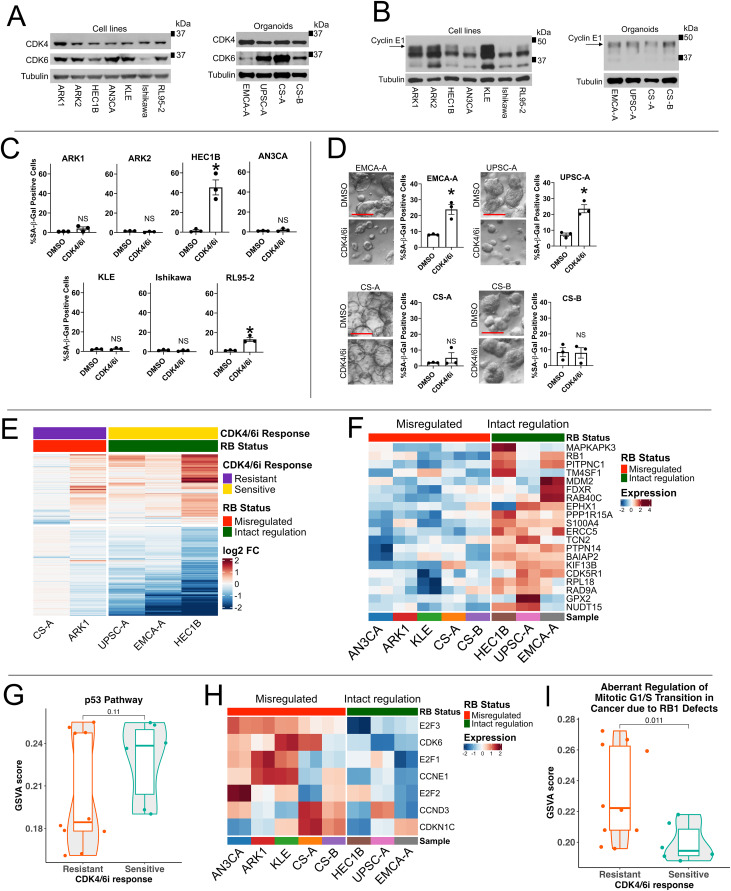
Endometrial carcinoma cell sensitivity to G1/S targeted therapies correlates with RB regulatory status. **A** and **B,** Protein lysates were prepared from untreated cell lines (left in each panel) and organoid lines (right in each panel) and analyzed by western blot. In **A**, membranes for each cell type were probed for CDK4, stripped and re-probed for CDK6, and stripped and re-probed for tubulin as a loading control. In **B**, membranes for each cell type were probed for Cyclin E1, and then stripped and re-probed for tubulin as a loading control. An arrow indicates the main Cyclin E1 isoform, and below that, low molecular weight Cyclin E1 isoforms are also visible. **C** and **D,** Cell and organoid lines were treated with media containing either 0.25 µM of the CDK4/6 inhibitor (CDK4/6i) Abemaciclib or vehicle (DMSO). Cell lines were treated for four doubling times while organoids were treated for 10 days. Cells were harvested at the appropriate timepoint, stained for senescence-associated β-galactosidase (SA-β-Gal) activity using the CellEvent Senescence Green Flow Cytometry Assay Kit, and analyzed by flow cytometry. Cells positive for the SA-β-Gal activity detection probe signal were considered senescent and referred to as SA-β-Gal positive cells. Bar graphs show the average percentage of SA-β-Gal positive cells from three separate experiments for each model, and error bars represent standard error of the mean. *P* values were calculated using a paired t-test compared to DMSO. * = *P* < 0.05 and NS= not significant. Models showing a significant increase in senescence post-CDK4/6i treatment were designated as CDK4/6i sensitive for later analyses. For the organoids in **D**, representative photos for each organoid treated with either DMSO (top) or CDK4/6i (bottom) for nine days are shown to the left of the bar graph. Scale bars represent 200 µm. Representative photos were cropped in the same way from larger photos. **E–I,** Cell lines were treated with media containing vehicle (DMSO) or 0.25 µM of the CDK4/6i Abemaciclib for one doubling time, and organoids were treated with media containing either DMSO or 0.25 µM of the CDK4/6i Abemaciclib for 24 hours for **E**. Another set of cell or organoid lines were only treated with media containing DMSO for 24 hours for analysis in **F–I**. Total RNA was prepared from all samples and underwent bulk RNA sequencing which was analyzed by the following methods. Each individual sample was sequenced in duplicate to provide technical replicates for validation. In **E**, the DMSO and CDK4/6i treated samples were compared to each other. A heatmap of all genes significantly differentially expressed (FDR-adjusted *P* value < 0.05 and |log_2_FC| > 0.5) in at least one sample between DMSO control and CDK4/6i treatment (*n* = 950 genes total) is shown, annotated by response to CDK4/6i and RB regulation status. Genes were clustered by *k*-means clustering with *k* = 2. In **F**, the baseline transcriptional profiles of the DMSO only-treated cell lines and organoids were compared to each other. A heatmap of Z-score normalized expression of the top 20 significantly differentially expressed core enrichment genes between RB intact regulation and misregulated samples involved in the p53 pathway (defined by the Hallmark p53 pathway gene set), split by RB regulation status, is shown. Significance was determined using the FDR-adjusted *P*-values. In **G**, the baseline transcriptomic profiles of the DMSO only-treated cell lines and organoids were compared and grouped based on sensitivity or resistance to CDK4/6i treatment. Gene Set Variation Analysis (GSVA) was used to calculate single-sample enrichment scores for each replicate of each model for baseline enrichment in the Hallmark p53 pathway gene set; high scores denote the upregulation of the gene set, and low scores denote the downregulation of the gene set. The violin plot shows GSVA score comparison of baseline enrichment in the Hallmark p53 pathway gene set between replicates of CDK4/6i sensitive and resistant models. *P*-values are derived from a Mann-Whitney U test comparing GSVA scores for sensitive and resistant samples. In **H**, the baseline transcriptional profiles of the DMSO-treated cell lines and organoids were compared to each other. A heatmap of Z-score normalized expression of the top seven significantly differentially expressed core enrichment genes between RB intact regulation and misregulated samples from the Reactome Aberrant Regulation of Mitotic G1/S Transition in Cancer Due to RB1 Defects gene set, split by RB regulation status, is shown. Significance was determined using the FDR-adjusted *P*-values. In **I**, the baseline transcriptomic profiles of the DMSO only-treated cell lines and organoids were compared and grouped based on sensitivity or resistance to CDK4/6i treatment. GSVA was used to calculate single-sample enrichment scores for each replicate of each model for baseline enrichment in the Reactome Aberrant Regulation of Mitotic G1/S Transition in Cancer Due to RB1 Defects gene set; high scores denote the upregulation of the gene set, and low scores denote the downregulation of the gene set. The violin plot shows GSVA score comparison of baseline enrichment in the Reactome Aberrant Regulation of Mitotic G1/S Transition in Cancer Due to RB1 Defects gene set between replicates of CDK4/6i resistant and sensitive samples. *P*-values were calculated as in **G**.

In terms of resistance, we first tested for expression of CDK4 and CDK6 by western blot using siRNA validated antibodies (Fig. 2A; Supplementary Fig. S5D). CDK4 expression was similar among the cell lines, while ARK1, ARK2, AN3CA, KLE, and RL95-2 models revealed increased CDK6 expression over other cell lines (Fig. 2A). CDK4 expression was mostly similar among organoid models; however, CS-A, CS-B, and UPSC-A revealed increased CDK6 expression compared to EMCA-A (Fig. 2A). The increased CDK6 expression observed in some of these models could contribute to RB misregulation and CDK4/6i resistance.

Finally, we tested for increased Cyclin E1 expression as some of the models harbor genomic alterations in *CCNE1*. Specifically, KLE cells are reported to have a *CCNE1* amplification while ARK1 and ARK2 cells are reported to have *CCNE1* copy number gains (33, 50, 51). Copy number analysis was not performed on the organoid models. We tested for Cyclin E1 overexpression by western blot using an siRNA validated antibody, and found varying Cyclin E1 expression levels in all models, including varying expression of low molecular weight isoforms at least in the EC cell lines (Fig. 2B; Supplementary Fig. S5E; refs. 50, 52, 53). Among the cell lines, we detected the highest expression of Cyclin E1 in the *CCNE1* amplified KLE model with slightly lower levels in the *CCNE1* copy number gain ARK1 and ARK2 models and much lower levels in the other four cell lines (33, 50). Cyclin E1 expression was similar among the organoid models (Fig. 2B). The increased Cyclin E1 expression in some of the models could contribute to any observed RB misregulation and CDK4/6i resistance.

CDK4/6is cause tumor reduction via induction of senescence (47). Thus, we tested for upregulation of activity of senescence-associated β-galactosidase (SA-β-Gal), a marker of senescent cells, post-CDK4/6i treatment versus vehicle in all models by flow cytometry using optimized dosing and timing (Supplementary Fig. S6A for dose/time optimization, Fig. 2C and D; ref. 47). Any model revealing a significant increase in senescent SA-β-Gal positive cells post-CDK4/6i treatment compared to vehicle was designated as CDK4/6i sensitive. We found that the models with intact RB regulation including HEC1B, RL95-2, EMCA-A, and UPSC-A, regardless of *TP53* status, showed a significant increase in senescent SA-β-Gal positive cells upon treatment with a CDK4/6i (Fig. 2C and D). *RB1* WT, RB protein expressing models with RB misregulation, including CS-A, CS-B, ARK1, ARK2, AN3CA, and KLE cells, as well as, *RB1* mutant Ishikawa cells did not show an increase in senescent SA-β-Gal positive cells upon CDK4/6i treatment (Fig. 2C and D). CDK4/6i resistance and RB misregulation in the models could be caused by any of the known mechanisms of resistance we tested including increased expression of CDK4, CDK6, or Cyclin E1 on western blot described above (Fig. 2A–D; Table 1; ref. 11). Overall, these results suggest that intact RB regulation, but not *TP53* mutation status, may predict CDK4/6i sensitivity in EC cells.

To test this possibility, we performed correlation testing with the goal of showing that intact RB regulation, as shown by functional assays, correlates with sensitivity to a CDK4/6i, as shown by increased senescence upon CDK4/6i treatment in EC cells. Specifically, we compared the fold change in the percentage of (i) G1-phase cells representing RB regulatory status, or (ii) SA-β-Gal positive cells representing CDK4/6i sensitivity, both after treatment with CDK4/6i versus vehicle amongst all models (Supplementary Fig. S6B). We found that sensitivity to CDK4/6 inhibition correlates with RB regulatory status, as suggested by the moderately positive correlation coefficient and significant *P*-value (*R* = 0.67, *P* = 0.025; Figs. 1G, 1I, 2C, and 2D; Supplementary Fig. S6B). This fits with findings in other disease settings where CDK4/6is are used (11).

We tested if RB regulatory status corresponded to transcriptional changes upon CDK4/6i treatment in responsive models. We treated a subset of PDOs and cell lines with vehicle or CDK4/6i and performed RNA sequencing. Upon differential gene expression analysis, we found that CDK4/6i sensitive models which had intact RB regulation exhibited a greater degree of differential expression upon CDK4/6i treatment compared to RB misregulated-CDK4/6i resistant models (Fig. 2E).

Given that RB regulatory status defined by functional assays corresponds with CDK4/6i sensitivity and CDK4/6i-induced transcriptional changes (Fig. 2E; Supplementary Fig. S6B), we next asked if RB regulatory status could be detected in the baseline transcriptional profiles of our EC models, which could then also be used as a marker for CDK4/6i response. We analyzed the baseline expression profiles of a subset of *RB1* WT, RB protein expressing cell or organoid lines classified as having intact RB regulation or misregulated RB, and thus being CDK4/6i sensitive or resistant respectively, based on our functional assays (Fig. 2F–I). We searched for differences in expression of gene sets which support RB function, specifically those in p53 and RB transcriptional pathways enriched for cell cycle regulatory genes, between our models with intact RB regulation and those with misregulated RB. Our analysis revealed that the RB but not the p53 gene set was appropriately transcriptionally up- or down-regulated in cells with intact RB regulation compared to RB misregulated cells (Fig. 2F–I). Specifically, although *RB1* itself and some other genes in the p53 transcriptional pathway were upregulated in some of the cells with intact RB regulation, transcriptional upregulation of p53 pathway genes did not correlate with CDK4/6i response (Fig. 2F and G). In contrast, *CCNE1* and *CDK6*, which can cause CDK4/6i resistance when overexpressed, along with several other genes in a gene set marking defective or misregulated RB-mediated cell cycle control, were appropriately transcriptionally downregulated in cells with intact RB regulation compared to RB misregulated cells (Fig. 2H). This decreased expression of these cell cycle genes indicating intact RB regulation correlated with CDK4/6i sensitivity (Fig. 2I; ref. 11). This suggests that baseline transcriptional profiles indicating intact RB regulation correlate with functional assay-defined CDK4/6i sensitivity and intact RB regulation in EC cells.

Taken together, these results suggest that intact RB regulatory status in cells expressing RB protein and without *RB1* mutations whether determined through functional assays on live cells or via transcriptional profiling of EC cells, corresponds to sensitivity to CDK4/6is regardless of *TP53* genomic status in a limited number of models. This is particularly exciting for the *TP53* mutant models HEC1B and UPSC-A, as these patients might currently be excluded from CDK4/6i trials based on their *TP53* status and/or possibly histology.

### Sensitivity to G2/M transition or mitosis targeted therapies does not correlate with p53 functional or RB regulatory status

**Figure 3:**
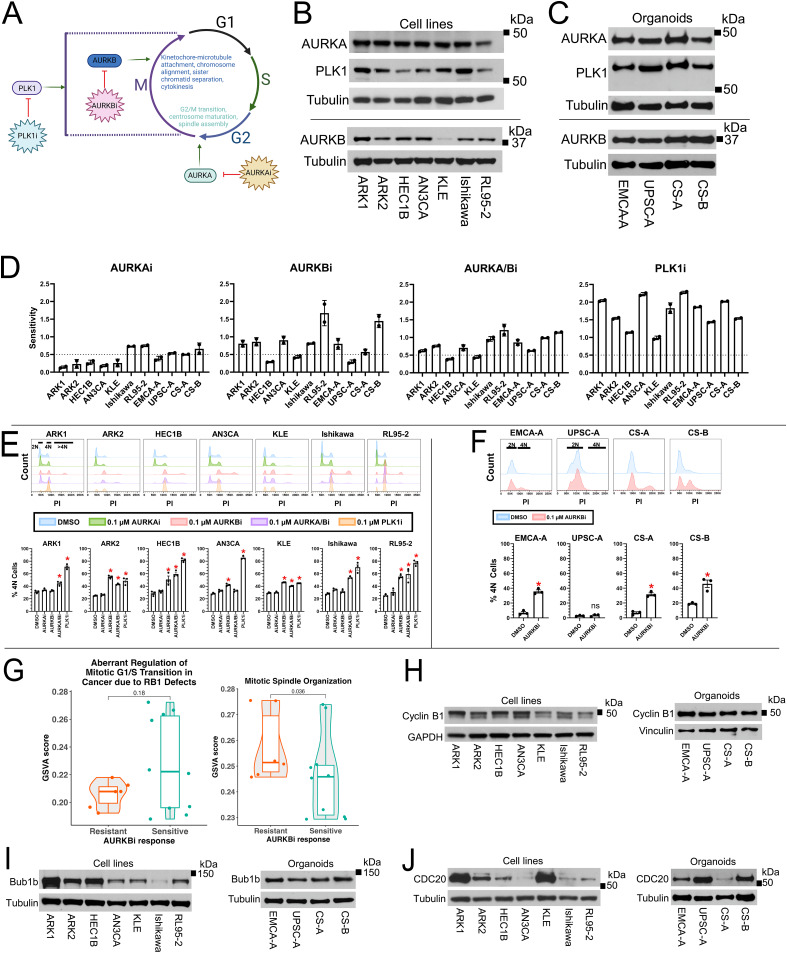
Endometrial carcinomas with transcriptionally evident mitotic spindle organization or mitotic progression regulatory defects are sensitive to Aurora Kinase B inhibition. **A,** Cartoon illustrating the different kinases governing the G2/M transition and mitotic progression and what part of the G2/M transition or mitosis the inhibitors of these kinases target (Aurora kinase A (AURKA), Aurora kinase B (AURKB), and Polo-like kinase 1 (PLK1)). **B** and **C,** Protein lysates were prepared from untreated cell lines (**B**) and organoid lines (**C**) and analyzed by western blot. On the top in each panel, one gel was run and those membranes were probed for AURKA, stripped and re-probed for PLK1, and stripped and re-probed for tubulin as a loading control. On the bottom in each panel, a second gel was run, and those membranes were probed for AURKB and then stripped and re-probed for tubulin. **D,** Organoids and cell lines were treated with a dose curve of the AURKA inhibitor MK5108 (AURKAi), the AURKB inhibitor Barasertib (AURKBi), the dual AURKA/B inhibitor Alisertib (AURKA/Bi), or the PLK1 inhibitor Onvansertib (PLK1i), and survival was assessed after five days by CellTiter-Glo. The day five CellTiter-Glo reads along with initial CellTiter-Glo reads of untreated cells taken on the day the cells were plated and treatment initiated were then used to generate a growth rate corrected dose-response curve for each model with each agent to compensate for the varying cell cycle rates of the different models. The area over the growth rate corrected dose-response curve (AOC) was then calculated for each model with each agent. The AOC represents the sensitivity of the model to the agent (larger AOC = greater sensitivity). For additional details, please see Supplementary Materials and Methods. The experiment was repeated twice for each model with each agent. The bar graphs show the average sensitivity for each model to each agent with error bars representing standard deviation. A dashed line on each graph at 0.5 denotes an arbitrary cutoff for sensitivity of a cell or organoid line, and all models with an average sensitivity of 0.5 or above were deemed sensitive to the agent tested. **E** and **F,** In E, cell lines were treated with media containing either vehicle (DMSO) or 0.1 µM of AURKAi, AURKBi, dual AURKA/Bi, or PLK1i for 24 hours, and in **F** organoids were treated with media containing either vehicle (DMSO) or 0.1 µM AURKBi for 24 hours. The differently treated models then underwent bromodeoxyuridine (BrdU)/propidium iodide (PI) for cell lines or 5-Ethynyl-2′-deoxyuridine (EdU)/PI for organoids cell cycle flow cytometry profiling followed by analysis of (i) the PI data alone for percentages of different DNA content cells [4N shown in **E** and **F** here with additional corresponding 2N and greater than 4N (for cell lines only) DNA content cell bar graphs from the same data in Supplementary Fig. S11A and S11C], and (ii) the combined PI/BrdU or PI/EdU data (shown in Supplementary Fig. S11B and S11D stacked bar graphs). In each panel, the top row shows representative PI profile plots from one of three experiments generated by analysis of the PI data alone for 2N, 4N, or greater than 4N (cell lines only) DNA content cells with examples marking each DNA content drawn on the top of the first PI plot in each panel and for the organoids also for UPSC-A. The color code for the different treatments is under the profile plots in each panel. On the bottom are bar graphs demonstrating the percentage of cells with 4N DNA content based on analysis of the PI data alone for DNA content with bars representing the average of three experiments and error bars representing standard error of the mean. * = *P* < 0.05 compared to DMSO for each treatment by an ordinary one-way ANOVA with Šídák's multiple comparisons test for **E** and with paired t-test for **F**. If there is no *, then the comparison was not significant in **E**. ns = not significant in **F**. Please see Supplementary Figs. S8 and S9 for both the PI alone and the combined PI/BrdU or PI/EdU flow cytometry gating strategies, Supplementary Fig. S11A and S11C for bar graphs showing the percentage of 2N or greater than 4N (for cell lines only) DNA content cells that correspond to (**E** and **F**) above for PI only analysis, and Supplementary Fig. S11B and S11D for stacked bar graphs demonstrating the percentage of 2N BrdU/EdU negative, BrdU/EdU positive (marking S phase), and 4N BrdU/EdU negative cells for combined BrdU/PI or EdU/PI analysis of the flow cytometry data shown in **E** and **F**. **G,** Bulk RNA sequencing was performed on total RNA prepared from a subset of the cell lines and organoids treated only with DMSO. Each individual sample was sequenced in duplicate to provide technical replicates for validation. Please note that the data analyzed here is the same bulk RNA sequencing data from DMSO treated cells analyzed in Fig. 2F–I. Here in **G**, these baseline transcriptomic profiles of the different models were compared and grouped based on sensitivity or resistance of the model to AURKBi treatment. Gene Set Variation Analysis (GSVA) was used to calculate single-sample enrichment scores for baseline enrichment in each replicate of each model in either the Reactome Aberrant Regulation of Mitotic G1/S Transition in Cancer Due to RB1 Defects (left) or the GOBP Mitotic Spindle Organization (right) gene sets; high scores denote the upregulation of gene sets, and low scores denote the downregulation of gene sets. The violin plots show GSVA score comparison of baseline enrichment in the Reactome Aberrant Regulation of Mitotic G1/S Transition in Cancer Due to RB1 Defects (left) or the GOBP Mitotic Spindle Organization (right) gene sets between all replicates of AURKBi sensitive and resistant models. *P*-values are derived from a Mann-Whitney U test comparing GSVA scores for sensitive and resistant samples. **H–J,** Protein lysates were prepared from untreated cell lines (left in each panel) and organoid lines (right in each panel) and analyzed by western blot. In **H**, one gel was run for each cell type and then membranes were first probed for Cyclin B1 and then stripped and re-probed for either GAPDH for cell lines or Vinculin for organoids as a loading control. In **I**, a second set of gels were run for each cell type and then membranes were first probed for Bub1b and then stripped and re-probed for tubulin as a loading control. In **J**, a third set of gels were run for each cell type and then membranes were first probed for CDC20 and then stripped and re-probed for tubulin as a loading control.

We next tested for sensitivity to various G2/M transition or mitotic progression targeting therapies including PLK1, AURKA, or AURKB inhibitors which each target different parts of G2/M or mitotic progression, to search for any specific defects in EC cells (Fig. 3A; refs. 12, 54). PLK1 controls progression from G2 into mitosis and various aspects of mitotic progression; AURKA controls progression from G2 into mitosis, centrosome maturation, and bipolar spindle assembly; and AURKB controls kinetochore-microtubule attachment, chromosome alignment, sister chromatid separation, and cytokinesis (Fig. 3A; refs. 12–14, 29, 32). Sensitivity to one or more of these inhibitors might suggest that the cells harbor defects in regulation of mitotic progression that make them dependent on one of these regulatory kinases for survival.

We first assessed for expression of AURKA, AURKB, and PLK1 and found varying levels of expression in all models (Fig. 3B and C; Supplementary Fig. S7A). We next assessed for sensitivity of all models to an AURKAi, an AURKBi, a dual AURKA/Bi (19–21), and a PLK1i (Fig. 3D; ref. 35). We found that only five models had limited or borderline sensitivity just above the sensitivity threshold to AURKA inhibition, all but three of the models were sensitive to AURKB inhibition, all but two of the models were sensitive to dual AURKA/B inhibition largely at the higher doses where AURKB would also be inhibited (19–21), and all models were sensitive to PLK1 inhibition (Fig. 3D). Since PLK1 controls so many aspects of G2/M progression and/or mitosis, the broad sensitivity of EC cells to this agent made it difficult to determine where the defect causing the sensitivity might be. In contrast, the broader sensitivity of EC cells to AURKB inhibition but not AURKA inhibition suggested that many EC cells harbor defects which make them heavily reliant on AURKB to safely complete mitosis.

Given this, we assessed for the importance of AURKA and AURKB in EC cell lines by depletion to determine if the above results are specific to inhibition of these different kinases. We found that depletion of either protein led to decreased colony formation in EC cells with intact RB regulation or misregulated RB suggesting that both promote cell survival (Supplementary Fig. S7B). Thus, there is something unique about AURKB inhibition in EC cells given that both AURKA and AURKB promote EC cell survival but only inhibition of AURKB causes cytotoxicity, and we sought to determine what defects might be causing the observed sensitivity.

The sensitivity did not correspond to *TP53* status (Table 1). Specifically, 10 of the 11 models are *TP53* mutant, and both *TP53* WT and mutant models demonstrated sensitivity to the AURKBi suggesting that *TP53* status does not correlate with AURKBi sensitivity, a result observed previously (Table 1; ref. 55).

We next addressed whether EC cell accumulation with a specific DNA content or in a specific phase of the cell cycle in response to AURKA, AURKB, AURKA/B, or PLK1 inhibition corresponds to sensitivity. We hypothesized that cells resistant to these different mitosis targeting agents would show signs of a G2/M or specific DNA content accumulation post-treatment, whereas those that were sensitive might lack the ability to arrest and may continue cycling with chromosomal abnormalities that might trigger apoptosis. To test for this possibility, we performed bromodeoxyuridine (BrdU)/propidium iodide (PI), or in the case of organoids 5-ethynyl-2′-deoxyuridine (EdU)/PI, cell cycle profiling on all models post-treatment with mitosis targeting agents and examined the data for signs of any post-treatment changes in cell cycle progression (Fig. 3E and F; Supplementary Figs. S8A–S8C, S9A–S9C, S10A, S10B, and S11A–S11D). These experiments required careful assessment of multiple aspects of the data as the different mitosis targeting agents being used can induce unique populations of cells.

Specifically, in this method, the cells are pulsed with BrdU or EdU, which is incorporated into the DNA of replicating cells and marks cells in S phase, while PI marks all of the DNA and reveals total DNA content of the cells (56). Cells with two copies of each chromosome have a 2N DNA content and are generally thought of as being in G1 phase if the cells are BrdU/EdU negative or early S phase if the cells are BrdU/EdU positive (56). Cells with four copies of each chromosome have a 4N DNA content and are generally thought of as being in late S phase if the cells are BrdU/EdU positive or G2/M phase if the cells are BrdU/EdU negative (56). However, it is also possible that a 4N DNA content cell may be in G1 phase, especially after release from a prolonged mitotic arrest or accelerated mitotic progression both followed by failed cytokinesis, which may be induced by some of the mitosis targeting agents used here (57). Finally, many cancer cells have abnormal larger numbers of chromosomes and thus a greater than 4N (>4N) DNA content at baseline. Of note, AURKB inhibitors in particular are known to induce some cells to accumulate with a 4N DNA content in G1 phase or a >4N DNA content which may be difficult to segregate by traditional G1/S/G2-M classification (54, 58, 59). Given this, we analyzed our BrdU-PI/EdU-PI data in multiple ways as described below.

Prior to initial experiments, we tested for the optimal drug dose to use for these experiments by treating all cell lines with a dose curve of AURKBi and performing flow cytometry cell cycle profiling 24 hours later (Supplementary Fig. S10). We examined the PI data from the cell cycle profiling for accumulation of 4N and greater than 4N DNA content cells, and based on this data we settled on 0.1 µM which induced variable 4N and greater than 4N DNA content cell accumulation in all models (Supplementary Fig. S10; ref. 54). We then treated all cell lines with each agent and organoid lines only with the AURKBi, performed BrdU/PI or EdU/PI cell cycle profiling, and assessed for accumulation of cells with a specific DNA content or in a specific cell cycle phase which may correlate with therapeutic response. This data is shown in Fig. 3E and F, with representative gating strategies shown in Supplementary Figs. S8 and S9, corresponding 2N and greater than 4N (for cell lines only) cell quantification shown in Supplementary Fig. S11A and S11C, and combined BrdU/PI or EdU/PI analysis of and bar graphs for the same data shown in Supplementary Fig. S11B and S11D.

To assess for correlation solely between accumulation of a specific DNA content and drug sensitivity, we examined the PI data alone from these BrdU/PI or EdU/PI experiments to quantify cells with 2N, 4N, and >4N DNA content (Fig. 3E and F; Supplementary Fig. S8 for representative gating strategy, and Supplementary Fig. S11A and S11C for corresponding 2N and greater than 4N cell quantification). To account for the importance of S phase in delineating different 2N and 4N DNA content populations, especially in slower cycling cell lines and organoid lines where >4N DNA content cells did not emerge, we also examined the same combined BrdU/PI or EdU/PI data for the percentage of non-S phase (BrdU/EdU negative) 4N DNA content cells (Supplementary Fig. S9 for gating strategy, and Supplementary Fig. S11B and S11D for combined BrdU/PI and EdU/PI analysis and bar graphs of the data in Fig. 3E and F). While this cell population might normally be classified as being in G2/M, they are referred to as 4N BrdU/EdU negative cells for AURKBi experiments in this study, because after AURKBi treatment at least some of these 4N DNA content cells could potentially also be in G1 phase as a result of AURKBi-induced mitotic progression with failed cytokinesis (54).

For the cell lines, we found that a post-treatment increase in the percentage of 4N or greater than 4N DNA content cells by PI analysis or an increase in 4N DNA content BrdU negative cells as shown by combined BrdU/PI analysis at the single post-treatment timepoint tested did not match well with response to AURKBi or any other agent (Fig. 3D and E; Supplementary Fig. S11A and S11B). Similarly, for the organoid lines, we found that a post-AURKBi increase in the percentage of 4N DNA content cells by PI analysis alone or 4N DNA content EdU negative cells by combined EdU/PI analysis did not match AURKBi sensitivity (Fig. 3D and 3F; Supplementary Fig. S11C and S11D). Organoid models did not accumulate with >4N DNA content at the timepoint analyzed.

To formally verify that no marker of altered cell cycle progression from BrdU/PI or EdU/PI profiling correlated with AURKBi response, we performed correlation testing. We compared AURKBi sensitivity and the fold change in the percentage of (i) 4N DNA content BrdU/EdU negative cells marked by BrdU-PI/EdU-PI analysis, (ii) 4N DNA content cells marked by PI analysis alone, or (iii) greater than 4N DNA content cells for cell lines only after PI analysis alone, all after treatment with AURKBi versus control (Supplementary Fig. S11E). We found no correlation between sensitivity with any of the above three cell cycle profiling results, as indicated by a weak correlation coefficient and an insignificant *P*-value in each case [(i) *R* = 0.24, *P* = 0.47; (ii) *R* = 0.11, *P* = 0.76; (iii) *R* = 0.13, *P* = 0.78; Supplementary Fig. S11E]. This suggested that finer mapping of the ability to arrest at a specific point in the G2/M transition or during mitotic progression might be needed to help detect a specific defect or weakness linked to AURKBi sensitivity.

Thus, we next assessed baseline transcriptomic profiles of EC cell and organoid lines for cell cycle regulation expression profiles that correlated strongly with AURKBi response. We performed single-sample Gene Set Variation Analysis on transcriptomic profiles of baseline cell lines and PDOs. We found that enrichment scores for a gene set linked to dysfunction or misregulation of RB cell cycle control were not significantly different between sensitive and resistant lines, fitting with our RB functional assay data not corresponding to AURKBi sensitivity (Table 1; Fig. 3D and G). In contrast, we found that the enrichment scores for the mitotic spindle organization gene set, which includes mostly genes encoding proteins involved in assembly/organization of mitotic spindles required for chromosome alignment and segregation during mitosis and also a small subset of genes encoding proteins that facilitate/participate in or are targeted by the spindle assembly checkpoint, were significantly different for AURKBi sensitive compared to resistant models (Fig. 3G). This result suggests that alterations or weaknesses in mitotic spindle organization and/or possibly the spindle assembly checkpoint, which would be critical in ensuring faithful chromosome segregation in spindle organization defective cells, correlate with increased response to AURKB inhibition (57). To help validate these transcriptional findings, we examined baseline protein expression levels of Cyclin B1 which is important in regulating mitotic entry and exit, Bub1b (also known as BubR1) which participates in the spindle assembly checkpoint, and CDC20 which is regulated by the spindle assembly checkpoint and ultimately aids in mitotic exit (60). Cyclin B1 expression was similar between the cell lines and also similar among the organoid models (Fig. 3H; Supplementary Fig. S12A). Bub1b expression was variable among the cell lines but similar among the organoid lines, while CDC20 revealed variable expression within the cell lines and also among the organoid models (Fig. 3I and J; Supplementary Fig. S12B and S12C). It is possible that strong up- or down- regulation of Bub1b or CDC20 may contribute to alterations or weaknesses in the regulation of mitotic progression in a model. This suggests that the functional assays indicating sensitivity to mitosis targeting therapies correspond well with transcriptional profiles indicating a weakness or alteration in mitotic spindle organization or possibly the spindle assembly checkpoint.

Taken together, these results suggest that some EC cells may respond to inhibition of AURKB, but that response does not correlate strongly with RB regulatory or p53 functional or genomic status. Rather it may correlate with defects in mitotic spindle assembly/organization or possibly defects in the spindle assembly checkpoint, on which spindle organization challenged cells would be heavily reliant. Finer mapping of mitotic progression than BrdU-PI/EdU-PI cell cycle profiling is likely needed to observe and better define such defects.

### EC cells with defects in regulating mitotic progression post-AURKBi treatment are more sensitive to AURKB inhibition

**Figure 4:**
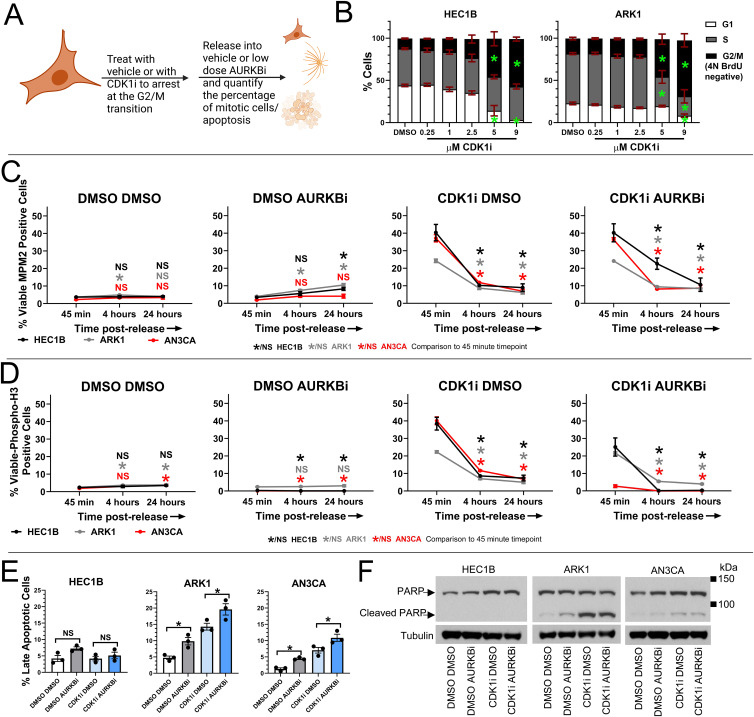
Aurora kinase B inhibitor sensitive endometrial carcinoma cells reveal more rapidly decreasing percentages of mitotic cells than resistant cells in the setting of Aurora kinase B inhibition. **A,** A cartoon demonstrating the experimental setup is shown. **B,** HEC1B and ARK1 cells were treated with vehicle (DMSO) or a dose curve of the CDK1 inhibitor (CDK1i) Ro-3306 for 16 hours. The cells then underwent bromodeoxyuridine (BrdU)/propidium iodide (PI) cell cycle flow cytometry profiling. Shown here are bar graphs with bars representing the percent of cells in each different cell cycle phase from three independent replicates with error bars representing standard error of the mean. G1 represents 2N DNA content BrdU negative cells, G2/M represents 4N DNA content BrdU negative cells, and S phase represents BrdU positive cells. * = *P* < 0.05 compared to DMSO for the specific cell cycle phase by an ordinary two-way ANOVA with Dunnett’s multiple comparisons test. If there is no *, then the comparison was not significant. The color code for the cell cycle phase is on the far right of the graphs. Please see Supplementary Fig. S12D for the flow cytometry gating strategy for the data in **B**. **C** and **D,** HEC1B, ARK1, or AN3CA cells were first treated with media containing either vehicle (DMSO) or CDK1i for 16 hours to synchronize cells at the G2/M transition, cells were washed, and then cells were treated with media containing vehicle (DMSO) or the Aurora kinase B inhibitor (AURKBi) Barasertib and analyzed by flow cytometry at 45 minutes (min), 4 hours, and 24 hours post-release for various mitotic markers. The average percentage of (**C**) Viable MPM2 positive cells or (**D**) Viable histone H3 phosphorylated on serine 10 (Phospho-H3) positive cells at each timepoint for each cell line from three separate experiments is plotted in line graphs for each treatment. The individual points for each cell line in the individual graph for each treatment represent the average of three experiments, and error bars represent standard error of the mean. An ordinary one-way ANOVA with Šídák’s multiple comparisons test was performed to assess the significance of the difference between either the 4 hour or the 24 hour timepoint and the 45 minute timepoint for each cell line within each treatment. The color code for the cell lines is shown below one of the graphs on the far left. The color code for the statistical markers is underneath the graphs in the middle. * = *P* < 0.05, and NS = not significant compared to the 45 minute timepoint for the individual cell line with the individual drug combination, with the color of the * or letters corresponding to the cell line. Please see Supplementary Fig. S13A for antibody validation, Supplementary Fig. S13B for the gating strategy, Supplementary Fig. S14A for analysis of the same data to show Phospho-H3/MPM2 double positive cells, and Supplementary Fig. S14B and S14C for an additional representation of the data with additional statistical comparisons for the flow cytometry data in **C** and **D**. **E** and **F,** HEC1B, ARK1, and AN3CA cells were treated with vehicle (DMSO) or CDK1i for 16 hours, washed, and then treated with media containing vehicle (DMSO) or AURKBi for 24 hours. Cells were then analyzed for apoptosis by flow cytometry in **E** or western blot in **F**. For flow cytometry analysis in **E**, cells were harvested at the appropriate timepoint and then immediately co-stained for Zombie NIR viability dye and Apotracker Green. Cells were then analyzed by flow cytometry, and the percentage of late apoptotic cells (Zombie viability dye and Apotracker Green double positive) was quantified. The bar graphs show the average percent of late apoptotic cells for each of the four treatments with the bars representing the average of three independent experiments with error bars representing standard error of the mean. For comparisons indicated by brackets over the treatment groups being compared, * = *P* < 0.05 and NS = not significant by an ordinary one-way ANOVA with Šídák’s multiple comparisons test. Please see Supplementary Fig. S16 for a representative gating strategy for this type of apoptosis flow cytometry data. For western blot analysis in **F**, protein lysates were prepared from the variously treated cells, the same amount of protein for each cell line for each treatment was loaded into a gel and run simultaneously to allow for comparison of markers between cell lines, and then membranes were analyzed by western blot. Membranes were first probed for PARP and cleaved PARP indicated by labels/arrows on the left and then stripped and re-probed for tubulin as a loading control. The cleaved PARP/PARP images shown are from the same exposure and can be compared for protein levels.

Thus, we next sought to test if mitotic spindle organization or spindle assembly checkpoint defects correspond with sensitivity to AURKBis. We tested this in an AURKBi resistant (HEC1B) and two AURKBi sensitive (ARK1 and AN3CA) cell lines. We sought to finely map the percentage of mitotic cells after treatment with mitosis altering agents such as an AURKBi (Fig. 4A). We hypothesized that for AURKBi sensitive cells we would observe a rapid decline in the percentage of mitotic cells in the setting of different types of mitotic perturbations, while AURKBi resistant cells would likely reveal a much slower decrease in the percentage of mitotic cells post-treatment.

To test this possibility, we first treated the cells with vehicle leaving them asynchronous or with an optimized dose of CDK1 inhibitor (CDK1i) to synchronize them at the G2/M transition and allow for finer assessment of changes in the percentage of mitotic cells immediately after release from G2 (Fig. 4A and B; Supplementary Fig. S12D for representative flow cytometry gating for Fig. 4B; ref. 58). We then washed these differentially treated cells and added media containing either vehicle or AURKBi (Fig. 4A). We then assessed the percentage of mitotic cells marked by changes in the percentage of cells positive for different mitotic markers by flow cytometry at an early (45 minute), mid (4 hour), and late (24 hour) timepoint post-release into AURKBi (Fig. 4C and D; Supplementary Figs. S13A, S13B, and S14A–S14D). We assessed for changes in the percentage of cells positive for two markers specific to mitotic cells, including (i) histone H3 phosphorylated on serine 10 (Phospho-H3), and (ii) MPM2 antigens which constitute an array of proteins phosphorylated during mitosis recognized by the MPM2 monoclonal antibody (57, 61). MPM2 antigen and Phospho-H3 levels are regulated similarly to proteins involved in controlling mitotic progression. For example, Cyclin B1 binds to CDK1 upon mitotic entry to promote mitotic progression and is ubiquitinated and targeted to be degraded by the anaphase promoting complex/cyclosome (APC-C) together with CDC20 to promote mitotic exit (62). The APC-C also indirectly controls MPM2 antigen and Phospho-H3 levels by contributing to the inactivation of mitotic kinases either (i) responsible for phosphorylation of MPM2 antigens or histone H3, or (ii) responsible for or contributing to the suppression of phosphatases that would dephosphorylate MPM2 antigens or Phospho-H3 (62, 63). We anticipated that in cells with intact mitotic progression regulation, the percentage of MPM2 and Phospho-H3 positive cells will be increased immediately after the CDK1i is washed out, signifying mitotic entry, and then will either (i) remain stable or decrease more slowly over time than cells with weaker control upon release into a mitosis targeting agent, signifying a mitotic arrest, or (ii) more rapidly decrease upon release into vehicle which would signal release from the synchronization. In contrast, in cells with mitotic progression regulatory defects, we anticipate that the percentage of MPM2 and Phospho-H3 positive cells will be increased immediately after the CDK1i is washed out, signifying mitotic entry, and then will rapidly decrease upon release into either vehicle or a mitosis targeting agent as they are likely unable to arrest in response to mitotic perturbations. We assessed both Phospho-H3 and MPM2 antigens since Phospho-H3 is an AURKB substrate, and it is possible that the percentage of Phospho-H3 positive cells may be decreased post-AURKBi as a result of the AURKBi engaging AURKB but that these decreases may not accurately reflect the effects of the AURKBi on the percentage of mitotic cells (30, 54). MPM2 antigens include many mitotic proteins which would not all be AURKB targets and should allow for assessment of the percentage of mitotic cells even if Phospho-H3 does not. The gating strategy for these experiments is shown in Supplementary Fig. S13. The data for these experiments are shown in Fig. 4C and D, with double positive marker analysis in Supplementary Fig. S14A, with an additional representation of the same data with additional statistical comparisons shown in Supplementary Fig. S14B–S14D, and with BrdU/PI cell cycle profiling in the setting of these treatments in Supplementary Fig. S15A and S15B.

All three lines revealed only very small increases in the percentage of MPM2 positive cells 24 hours after asynchronous cells were treated with AURKBi compared to either the respective 45 minute vehicle-AURKBi timepoint (Fig. 4C) or the 24 hour vehicle only control (Supplementary Fig. S14B). In contrast, all three lines revealed a significantly increased percentage of MPM2 positive cells 45 minutes post-CDK1i release compared to their 45 minute asynchronous vehicle controls, indicating that all three lines were synchronized at the G2/M transition initially and entered mitosis (Fig. 4C; Supplementary Fig. S14B). Upon release into vehicle post-CDK1i treatment, all three lines showed similarly decreasing percentages of MPM2 positive cells over time (Fig. 4C; Supplementary Fig. S14B). In contrast, upon release into AURKBi post-CDK1i treatment, ARK1 and AN3CA cells showed a larger decrease in percentages of MPM2 positive cells at 4 hours compared to HEC1B cells, indicating a more rapid decrease in the percentage of mitotic cells in ARK1 and AN3CA cells (Fig. 4C; Supplementary Fig. S14B). At 24 hours post-release from CDK1i into AURKBi, ARK1, AN3CA, and HEC1B cells revealed similarly decreased percentages of MPM2 positive cells, consistent with an eventual decrease in the percentage of mitotic cells for all models (Fig. 4C; Supplementary Fig. S14B).

The Phospho-H3 results were harder to interpret as multiple cell lines revealed decreased percentages of Phospho-H3 positive cells that were most consistent with decreases due to the AURKBi engaging AURKB and blocking H3 phosphorylation possibly without altering the percentage of mitotic cells (30, 54). It was clear that the anti-Phospho-H3 antibody was functional in this assay, as all three lines revealed a significantly increased percentage of Phospho-H3 positive cells 45 minutes post-CDK1i release into vehicle compared to their 45 minute asynchronous vehicle controls, suggesting that all three lines were synchronized at the G2/M transition initially and entered mitosis post-release (Fig. 4D; Supplementary Fig. S14C). Upon release into vehicle post-CDK1i treatment, all three lines showed similarly decreasing percentages of Phospho-H3 positive cells over time (Fig. 4D; Supplementary Fig. S14C). However, there were greatly reduced percentages of Phospho-H3 single positive and Phospho-H3/MPM2 double positive cells for both HEC1B and AN3CA cells at all timepoints in the presence of AURKBi either in the setting of treatment of asynchronous cells or post-CDK1i release, making these results difficult to interpret (Fig. 4D; Supplementary Fig. S14A, S14C, and S14D). In contrast, in ARK1 cells the Phospho-H3 did not appear to have been altered in the same way by AURKBi at the dose tested. In ARK1 cells, the Phospho-H3 results were very similar to the MPM2 results, with percentages of Phospho-H3 single positive and Phospho-H3/MPM2 double positive cells rapidly decreasing upon release from CDK1i into AURKBi (Fig. 4C and D; Supplementary Fig. S14). In examining DNA content from BrdU/PI cell cycle flow cytometry profiles 24 hours after asynchronous cells were treated with AURKBi or after release from CDK1i into AURKBi, HEC1B and AN3CA cells revealed primarily 4N and greater than 4N DNA content cells, and ARK1 cells revealed populations of 2N, 4N, and greater than 4N DNA content cells (Supplementary Fig. S15). Given the decreased percentage of MPM2 positive cells post-CDK1i release into AURKBi in all three cell lines at the 24 hour timepoint, these results suggest that upon release from CDK1i, all three models do show decreasing percentages of mitotic cells at different rates in the setting of AURKBi treatment, but that the DNA content accumulation states, and possibly final outcomes, are different among the various models (Fig. 4C; Supplementary Fig. S15).

Given the more rapid decrease in the percentage of mitotic cells in ARK1 and AN3CA cells compared to HEC1B cells post-release from CDK1i into AURKBi, the differing DNA content 24 hours post-AURKBi treatment indicated by BrdU/PI cell cycle profiling, and the known sensitivity of ARK1 and AN3CA cells to AURKBi (Figs. 3D and 4C and Supplementary Fig. S15), we assessed for apoptosis in HEC1B, ARK1, and AN3CA cells at the final 24 hour timepoint for all four treatment conditions by (i) flow cytometry analysis of the percentage of late apoptotic cells, and (ii) western blot for the apoptosis marker cleaved PARP. Both ARK1 and AN3CA cells revealed (i) a small but significant increase in the percentage of late apoptotic cells by flow cytometry and increased expression of cleaved PARP in the setting of treatment of asynchronous cells with AURKBi compared to vehicle alone, and (ii) an increased percentage of late apoptotic cells by flow cytometry in the setting of AURKBi treatment after CDK1i release compared to vehicle after CDK1i release (Fig. 4E and F; Supplementary Fig. S16A–S16C for representative flow cytometry gating). HEC1B cells did not reveal increased late apoptotic cells by flow cytometry or increased Cleaved PARP with any treatment (Fig. 4E and F).

Taken together, these combined MPM2 time course, PI DNA content profiling, and apoptosis results post-CDK1i release into AURKBi suggest that ARK1 and AN3CA cells may harbor a mitotic progression regulatory defect allowing the percentage of mitotic cells to more rapidly decrease in the setting of AURKBi-induced abnormalities in kinetochore-microtubule attachment, chromosome alignment or separation, or cytokinesis, and that these cells are not able to tolerate traversing or possibly exiting mitosis, potentially as a 4N cell, and undergo apoptosis post-release into AURKBi (Fig. 4C–F; Supplementary Figs. S14 and S15). In contrast, these post-CDK1i release into AURKBi results suggest that HEC1B cells have a slower decrease in the percentage of mitotic cells in the setting of AURKBi-induced abnormalities, and although the cells do eventually reveal a decreased percentage of mitotic cells suggesting a mitotic exit, they are able to survive potential entry into G1 phase possibly in a 4N state indicated by the BrdU/PI data (Fig. 4C–F; Supplementary Figs. S14 and S15; ref. 57). The ARK1 results must be interpreted with the caveat that although the MPM2 results do indicate a more rapid decrease in the percentage of mitotic cells post-release into AURKBi, the fact that the AURKB substrate Phospho-H3 was still detectable in ARK1 cells at the lower AURKBi dose, when it was not easily detectable for HEC1B or AN3CA cells in the setting of AURKBi treatment, raises the possibility that ARK1 cells may have less engagement of AURKB by the AURKBi at the dose utilized here. Additionally, AURKB inhibitors can have many effects on mitotic progression making it hard to be certain where the mitotic progression regulatory defect in ARK1 and AN3CA cells is. Thus, to validate that the ARK1 model does have a mitotic progression regulatory defect and to better determine where that defect is in both the ARK1 and AN3CA models, it was necessary to follow the percentage of mitotic cells in the setting of treatment with an agent targeting a more limited aspect of mitosis.

### EC cells with defects in regulating mitotic progression post-nocodazole treatment are more sensitive to AURKB inhibition

**Figure 5:**
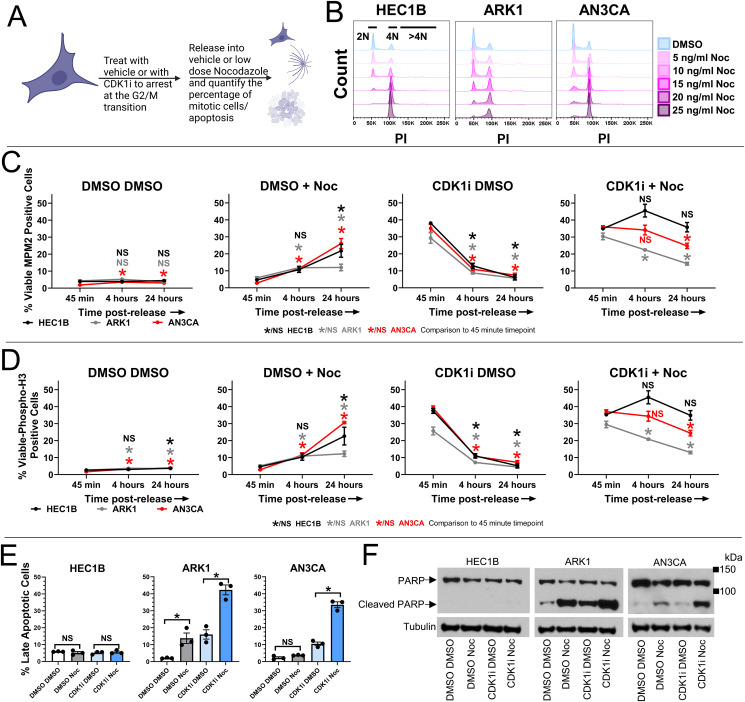
Aurora kinase B inhibitor sensitive endometrial carcinoma cells show more rapidly decreasing percentages of mitotic cells than resistant models in the setting of nocodazole-induced microtubule instability. **A,** A cartoon demonstrating the experimental setup is shown. **B,** HEC1B, ARK1, and AN3CA cells were treated for 24 hours with vehicle (DMSO) or a dose curve of nocodazole (Noc) and then underwent bromodeoxyuridine (BrdU)/propidium iodide (PI) flow cytometry cell cycle profiling. The experiment was repeated three times. Shown here are representative PI profile plots from analysis of the PI data alone for the percentage of cells with a specific DNA content from one of three independent replicates for each cell line. A line marking 2N, 4N, and greater than 4N (>4N) DNA content is shown on the top of the HEC1B plot. The color code for the doses is shown to the right of the plots. Please see Supplementary Fig. S17A for bar graphs of the percentage of cells with 2N, 4N, and greater than 4N DNA content from analysis of the PI data alone for all three replicates of this experiment, and also Supplementary Fig. S17B for stacked bar graphs showing G1, S, and G2/M (4N BrdU negative cells) combined BrdU/PI analysis of all three replicates of this experiment. **C** and **D,** HEC1B, ARK1, or AN3CA cells were first treated with media containing either vehicle (DMSO) or the CDK1 inhibitor (CDK1i) Ro-3306 for 16 hours to synchronize cells at the G2/M transition, cells were washed, and then cells were treated with media containing vehicle (DMSO) or 10 ng/mL nocodazole (Noc) and analyzed by flow cytometry at 45 minutes (min), 4 hours, and 24 hours post-release for various mitotic markers. The average percentage of (**C**) Viable MPM2 positive cells or (**D**) Viable histone H3 phosphorylated on serine 10 (Phospho-H3) positive cells at each timepoint for each cell line from three separate experiments is plotted in line graphs for each treatment. The individual points for each cell line in the individual graph for each treatment represent the average of three experiments, and error bars represent standard error of the mean. An ordinary one-way ANOVA with Šídák’s multiple comparisons test was performed to assess the significance of the difference between either the 4 hour or the 24 hour timepoint and the 45 minute timepoint for each cell line within each treatment. The color code for the cell lines is shown below one of the graphs on the far left. The color code for the statistical markers is underneath the graphs in the middle. * = *P* < 0.05, and NS = not significant compared to the 45 minute timepoint for the individual cell line with the individual drug combination, with the color of the * or letters corresponding to the cell line. Please see Supplementary Fig. S13A for antibody validation and S13B for the gating strategy for the data in **C** and **D**, Supplementary Fig. S18A for analysis of the Phospho-H3/MPM2 double positive cells for the flow cytometry data in **C** and **D**, and Supplementary Fig. S18B and S18C for an additional representation with additional statistical comparisons for the data in **C** and **D**. **E** and **F,** HEC1B, ARK1, and AN3CA cells were treated with vehicle (DMSO) or CDK1i for 16 hours, washed, and then treated with media containing vehicle (DMSO) or 10 ng/mL Noc for 24 hours. Cells were then analyzed for apoptosis by flow cytometry in **E** or western blot in **F**. For flow cytometry analysis in **E**, cells were harvested at the appropriate timepoint and then immediately co-stained for Zombie NIR viability dye and Apotracker Green. Cells were then analyzed by flow cytometry, and the percentage of late apoptotic cells (Zombie viability dye and Apotracker Green double positive) was quantified. The bar graphs show the average percent of late apoptotic cells for each of the four treatments with the bars representing the average of three independent experiments with error bars representing standard error of the mean. For comparisons indicated by brackets over the treatment groups being compared, * = *P* < 0.05 and NS = not significant by an ordinary one-way ANOVA with Šídák’s multiple comparisons test. Please see Supplementary Fig. S16 for a representative gating strategy. For western blot analysis in **F**, protein lysates were prepared from the variously treated cells, the same amount of protein for each cell line for each treatment was loaded into a gel and run simultaneously to allow for comparison of markers between cell lines, and then membranes were analyzed by western blot. Membranes were first probed for PARP and cleaved PARP indicated by labels/arrows on the left and then stripped and re-probed for tubulin as a loading control. The cleaved PARP/PARP images shown are from the same exposure and can be compared for protein levels.

Based on our combined CDK1i-AURKBi synchronization-release assay and transcriptional profiling results, we hypothesized that the mitotic progression regulatory defect present in AURKBi sensitive cells like ARK1 and AN3CA was most likely a spindle assembly checkpoint defect (Figs. 3G, 4C and D; Supplementary Fig. S14). Multiple drugs which stabilize or destabilize microtubules and directly engage the spindle assembly checkpoint are available (57). Thus, to further validate our results indicating a mitotic progression regulatory defect in ARK1 and AN3CA cells and to more directly assess if these cells harbor a spindle assembly checkpoint defect, we performed the same series of CDK1i synchronization/release experiments now with release into nocodazole which destabilizes microtubules (Fig. 5A; ref. 57). We first determined the optimal dose of nocodazole for synchronization/release assays by exposing ARK1, HEC1B, and AN3CA cells to a dose curve of nocodazole and performing BrdU/PI cell cycle profiling (Fig. 5B; Supplementary Fig. S17A and S17B). Higher doses of nocodazole were required to drive ARK1 and AN3CA cells to accumulate with 4N DNA content by PI analysis alone or in G2/M phase (marked by 4N DNA content BrdU negative cells) by combined BrdU/PI analysis over a 24 hour period compared to HEC1B cells, suggesting that ARK1 and AN3CA cells may have a weaker ability to arrest the cell cycle in response to the microtubule instability nocodazole induces (Fig. 5B; Supplementary Fig. S17A and S17B). We ultimately centered on 10 ng/mL nocodazole as an optimal dose, with a higher dose inducing stronger arrest with some toxicity being 20 ng/mL (Fig. 5B; Supplementary Fig. S17A and S17B).

Post-optimization, cells were treated with vehicle or CDK1i, washed, and treated with vehicle or low dose 10 ng/mL nocodazole and we then followed the percentage of mitotic cells post-release. These results are shown in Fig. 5C and D, with additional analysis showing double positive cells in Supplementary Fig. S18A, with an additional representation of the data with additional statistical comparisons in Supplementary Fig. S18B–S18D, and BrdU/PI cell cycle profiling in Supplementary Fig. S19A–S19C. All three cell lines revealed increased percentages of MPM2 single, Phospho-H3 single, and Phospho-H3/MPM2 double positive cells 24 hours after asynchronous cells were treated with nocodazole compared to (i) the 24 hour vehicle only control, where all increases were significant for each line (Supplementary Fig. S18B–S18D), and (ii) the 45 minute vehicle nocodazole timepoints for each line (Fig. 5C and D; Supplementary Fig. S18A–S18D). These increases in the percentage of cells positive for mitotic markers indicated that each line likely has a functional spindle assembly checkpoint able to induce mitotic arrest after nocodazole treatment, but that the strength of the activation of the checkpoint in the setting of this treatment was variable between the models given the varying percent increases in cells positive for the markers between models, with ARK1 cells having the smallest percentage increases (Fig. 5C and D; Supplementary Fig. S18; ref. 57). All three cell lines revealed significantly increased percentages of MPM2 single, Phospho-H3 single, and Phospho-H3/MPM2 double positive cells at 45 minutes post-CDK1i release compared to vehicle only controls at 45 minutes confirming that the cells were synchronized by the CDK1i (Supplementary Fig. S18B–S18D), and these percentages all decreased over time upon addition of vehicle containing media signifying release (Fig. 5C and D; Supplementary Fig. S18A). However, percentages of MPM2 single, Phospho-H3 single, and Phospho-H3/MPM2 double positive cells significantly decreased post-CDK1i release into nocodazole at 4 and 24 hours in ARK1 cells and 24 hours post-release in AN3CA cells, both compared to HEC1B cells in which percentages of cells positive for all mitotic markers remained higher over time post-release (Fig 5C and D; Supplementary Fig. S18). In examining BrdU/PI cell cycle flow cytometry profiles 24 hours after 10 ng/mL nocodazole alone or after release from CDK1i into 10 ng/mL nocodazole compared to vehicle or CDK1i/vehicle respectively, all cell lines showed increased 4N DNA content cells by PI analysis and increased G2/M phase cells (marked by 4N DNA content BrdU negative cells) by combined BrdU/PI analysis suggesting some degree of G2/M or 4N accumulation for each model (Supplementary Fig. S19).

To be certain that the 10 ng/mL nocodazole dose tested was not too low to reveal different phenotypes between models, we performed a similar series of experiments with a higher nocodazole dose of 20 ng/mL in HEC1B and ARK1 cells (Supplementary Fig. S20A–S20C and with an additional representation of the data and additional statistical comparisons in Supplementary Fig. S21A–S21C, and with cell cycle profiling in Supplementary Fig. S21D–S21F). In these experiments, we observed much stronger increases in the percentages of MPM2 single, Phospho-H3 single, and Phospho-H3/MPM2 double positive cells than with 10 ng/mL nocodazole 24 hours after nocodazole treatment alone versus vehicle suggesting a stronger response to the higher dose (Fig 5C and D; Supplementary Figs. S18, S20, and S21A–S21C). This pattern is similar to the results with 10 ng/mL nocodazole and again supports that both models have an intact spindle assembly checkpoint activated by nocodazole treatment, and given that the increases in the percentages of positive cells were less in the ARK1 compared to HEC1B cells, these findings again support that the checkpoint activation after nocodazole treatment is weaker in ARK1 cells (Fig. 5C and D; Supplementary Figs. S18, S20, and S21A–S21C). Strikingly, there was still a significant decrease in the percentage of MPM2 single, Phospho-H3 single, and Phospho-H3/MPM2 double positive cells for the ARK1 model but not the HEC1B model at 24 hours post-CDK1i release into nocodazole (Supplementary Figs. S20 and S21A–S21C). The HEC1B model maintained higher percentages of cells positive for all markers at 24 hours post-CDK1i release into nocodazole (Supplementary Figs. S20 and S21A–S21C). In examining BrdU/PI cell cycle flow cytometry profiles 24 hours after 20 ng/mL nocodazole alone or after release from CDK1i into 20 ng/mL nocodazole, HEC1B and ARK1 cells showed an even greater increase in the percentage of 4N DNA content cells by PI analysis and G2/M cells (marked by 4N DNA content BrdU negative cells) by combined BrdU/PI analysis than with 10 ng/mL nocodazole (Supplementary Figs. S19 and S21D–S21F). Overall, these results with higher dose nocodazole were similar to the lower dose, again showing decreased percentages of mitotic cells for ARK1 but not HEC1B cells after release from CDK1i into nocodazole (Fig. 5C and D; Supplementary Figs. S18A and S20).

Given that BrdU/PI cell cycle profiling indicated that HEC1B, ARK1, and AN3CA cells do accumulate with a 4N DNA content that is BrdU negative post-release from CDK1i into nocodazole, but that the ARK1 and AN3CA cells showed significantly decreasing percentages of MPM2, Phospho-H3, and Phospho-H3/MPM2 positive cells in this treatment setting while HEC1B cells did not, we hypothesized that ARK1 and AN3CA cells may be traversing or possibly exiting mitosis, perhaps as 4N cells, and undergoing apoptosis (Fig. 5C and D; Supplementary Figs. S18A and S20). Thus, we next sought to determine if the more rapid decrease in the percentage of mitotic cells upon release into nocodazole that we observed in ARK1 and AN3CA cells corresponded to increased apoptosis in these cells post-release. We tested for increased apoptosis by both flow cytometry and by western blot for cleaved PARP. We found that after treatment with 10 ng/mL nocodazole versus vehicle (i) ARK1 cells revealed increased late apoptotic cells by flow cytometry and increased cleaved PARP expression by western blot, and (ii) AN3CA cells revealed increased cleaved PARP expression by western blot (Fig. 5E and F; Supplementary Fig. S16). ARK1 and AN3CA cells revealed increased apoptosis markers by both metrics after treatment with CDK1i/10 ng/mL nocodazole versus CDK1i/vehicle (Fig. 5E and F; Supplementary Fig. S16). HEC1B cells did not reveal increases in any apoptotic markers with any 10 ng/mL nocodazole combination (Fig. 5E and F; Supplementary Fig. S16). Finally, ARK1 cells also revealed increased late apoptotic cells by flow cytometry and increased cleaved PARP expression by western blot after treatment with 20 ng/mL nocodazole versus vehicle or CDK1i/20 ng/mL nocodazole versus CDK1i/vehicle, while HEC1B cells only revealed a very small increase in late apoptotic cells by flow cytometry and increased cleaved PARP expression by western blot with CDK1i/20 ng/mL nocodazole compared to CDK1i/vehicle (Supplementary Figs. S16, S22A, and S22B).

Overall, these results indicate that all three cell lines likely have a functional spindle assembly checkpoint, but that the checkpoint activation in the setting of nocodazole treatment is of varying strength across models, with ARK1 cells being the weakest (Fig. 5C and D; Supplementary Figs. S18A and S20). These results also indicate that an additional mitotic progression regulatory defect in ARK1 and AN3CA cells may be a weakened ability to maintain a checkpoint induced mitotic arrest. Specifically, post-CDK1i release into nocodazole, the HEC1B cells, which had strong checkpoint activation, largely maintained the checkpoint induced arrest, and avoided undergoing apoptosis (Fig. 5C–F; Supplementary Figs. S18A, S20, S22A, and S22B). In contrast, post-CDK1i release into nocodazole, the AN3CA and ARK1 cells were unable to maintain the checkpoint induced arrest in mitosis as shown by their decreasing percentages of MPM2 and Phospho-H3 positive cells, and they more rapidly traversed and possibly exited mitosis, potentially in a 4N state indicated by BrdU/PI profiling, and underwent apoptosis potentially either during this mitotic exit or upon reaching G1 phase (Fig. 5C–F; Supplementary Figs. S18A, S20, S22A, and S22B; ref. 57). Taken together, these results support that EC cells with mitotic progression regulatory defects, including but not limited to a reduced strength in the activation of the spindle assembly checkpoint or an inability to maintain a spindle assembly checkpoint induced arrest, are more sensitive to AURKB inhibition regardless of p53 functional or RB regulatory status.

### RB regulatory status and mitotic progression regulatory capacity are effective markers of sensitivity to specific cell cycle targeting therapies *in vivo*

Based on our functional results, we hypothesized that RB regulatory status may correlate better with response to G1/S targeted therapies, while mitotic progression regulatory ability may correlate better with response to therapies which target specific aspects of mitosis. Here we sought to provide evidence that our transcriptional and functional studies matched *in vivo* response.

**Figure 6:**
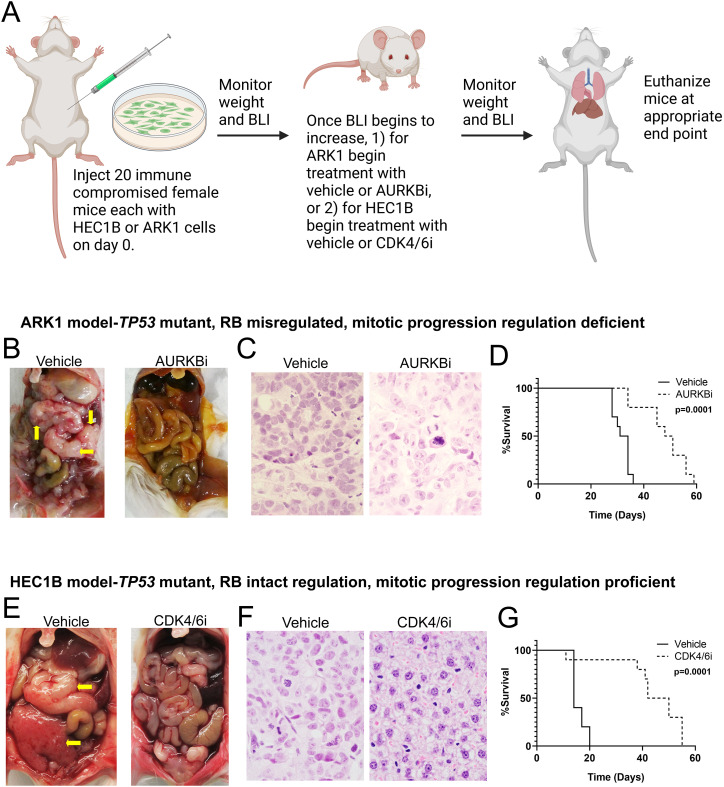
RB intact regulation and mitotic progression regulation deficient endometrial carcinoma cells demonstrate different responses to cell cycle targeting therapies *in vivo*. **A,** Cartoon demonstrating *in vivo* study experimental setup. For each study, after treatment initiation, mice were euthanized once they developed signs of morbidity; and then ascites volume was obtained, gross photos were taken, and residual tumor was harvested for all animals possible. **B–D,** Immune compromised mice were intraperitoneally injected with luciferized ARK1 cells. Mice were monitored by bioluminescent imaging (BLI) for tumor formation, and treatment with vehicle or the Aurora kinase B inhibitor (AURKBi) Barasertib was initiated once a BLI signal threshold was reached. Representative gross photos of vehicle and AURKBi treated animals are shown in **B**, with arrows indicating representative solid tumors. Please note that the gross photos were cropped from larger photos. Representative photos of hematoxylin and eosin (H&E) stained sections of residual tumor are shown for vehicle (left) and AURKBi (right) treated animals in **C**. Kaplan-Meier survival curves starting at day of treatment initiation are shown in **D **for the vehicle and AURKBi treated groups. A *P*-value was determined using the log-rank test, and *P *< 0.05 was considered significant. Please see Supplementary Fig. S22C for corresponding ascites data for this animal study. **E–G,** Immune compromised mice were intraperitoneally injected with luciferized HEC1B cells. Mice were monitored by BLI for tumor formation, and treatment with vehicle or the CDK4/6 inhibitor (CDK4/6i) Abemaciclib was initiated once a BLI signal threshold was reached. Representative gross photos of vehicle and CDK4/6i treated animals are shown in **E**, with arrows indicating representative solid tumors. Please note that the gross photos were cropped from larger photos. Representative photos of H&E stained sections of residual tumor are shown for vehicle (left) and CDK4/6i (right) treated animals in **F**. Kaplan-Meier survival curves starting at day of treatment initiation are shown in **G** for the vehicle and CDK4/6i treated groups. A *P*-value was determined using the log-rank test, and *P *< 0.05 was considered significant. Please see Supplementary Fig. S22D for corresponding ascites data for this animal study.

We intraperitoneally injected immune compromised mice with either the *TP53* mutant, RB misregulated, mitotic progression regulatory deficient ARK1 model or the *TP53* mutant, RB intact regulation, mitotic progression regulatory proficient HEC1B model, followed carefully for tumor formation via bioluminescent imaging (BLI), and then began treatment with vehicle or either AURKBi or CDK4/6i respectively (Fig. 6A). All animals in each study formed tumors as evidenced by increasing BLI signal intensity post-injection. The ARK1 model demonstrated grossly reduced tumor burden with histologic validation of tumor formation, increased survival, and significantly reduced ascites volume in the AURKBi treated animals compared to vehicle (Fig. 6B–D; Supplementary Fig. S22C). The median survival time was 32.5 days for vehicle treated mice and 49.5 days for AURKBi treated mice (Fig. 6D). The HEC1B model demonstrated grossly reduced tumor burden with histologic validation of tumor formation, increased survival, and a trend of reduced ascites volume in the CDK4/6i treated animals compared to vehicle (Fig. 6E–G; Supplementary Fig. S22D). The median survival time was 14 days for vehicle treated mice and 46 days for CDK4/6i treated mice (Fig. 6G).

The above *in vivo* results support our *in vitro* data and suggest that in addition to current molecular subtypes, EC patients may also be molecularly stratified as (i) *RB1* WT and RB protein expressing with either intact RB regulation or misregulated RB, and (ii) mitotic progression regulatory proficient or deficient, for potential later treatment with G1/S or mitotic progression targeted therapies respectively.

## Discussion

EC is one of the few cancer types with increasing incidence and mortality, and new therapies are desperately needed (1). Many ECs harbor either mutations or copy number alterations in genes which may alter their ability to control cell cycle progression, including but not limited to *TP53*, *CCNE1*, *RB1*, and/or *PTEN*; and the resulting cell cycle regulatory defects may be relevant therapeutic targets (2). However, functional analysis is necessary to determine if any cell cycle regulatory defects and subsequent therapeutic vulnerabilities result from such alterations. Here, using a panel of EC cell lines and PDOs, we assessed the impact of p53 and RB cell cycle regulatory functional deficiency or proficiency on sensitivity of EC cells to different cell cycle targeted therapies. *TP53* genomic and functional status had no impact on response to G1/S or mitotic regulatory kinase targeted therapies (Figs. 1–3). In contrast, intact RB regulation in EC cells with no *RB1* mutation and expressing RB protein, as indicated by both functional assays and baseline transcriptional profiles, correlated with sensitivity to G1/S targeting CDK4/6is (Figs. 1, 2, and 6E–G; Supplementary Fig. S6B). Additionally, defects in regulation of mitotic progression indicated by functional assays and baseline transcriptomic profiling correlated with sensitivity to mitosis targeting AURKBis (Figs. 3–5 and 6B–D; Supplementary Fig. S11E). Our findings have significant implications for EC biology, how ECs are currently molecularly stratified, and for future EC therapeutic exploration.

First, our CDK1i synchronization/AURKBi or nocodazole release experiments indicate that EC cells have varying spindle assembly checkpoint activation strength after nocodazole treatment, possible additional mitotic progression regulatory defects, and varying abilities to survive rapid or prolonged mitotic exit possibly as 4N cells, all of which may be therapeutically targetable. Specifically, ARK1 and AN3CA cells showed a more rapid decrease in the percentage of mitotic cells upon release from CDK1i into either AURKBi or nocodazole and subsequently underwent apoptosis (Figs. 4 and 5; Supplementary Figs. S14, S15, S18–S21, S22A, and S22B). This rapid decrease in the percentage of mitotic cells could be attributed to either the reduced strength of activation of the spindle assembly checkpoint in the setting of microtubule instability in ARK1 cells and/or possibly additional defects in both models which prevent them from maintaining a mitotic arrest when treated with AURKBi or nocodazole (Figs. 4 and 5; Supplementary Figs. S14A, S18A, and S20). The mechanism of the weakened spindle assembly checkpoint activation in ARK1 cells, the inability of ARK1 or AN3CA cells to maintain a checkpoint induced arrest, and/or possibly additional mitotic progression regulatory defects for both lines will be important areas for future investigation as they may represent relevant therapeutic targets or at least biomarkers for response to currently available mitosis targeting therapies. In contrast, HEC1B cells had strong activation of the spindle assembly checkpoint and maintained a higher percentage of mitotic cells for a longer period of time than ARK1 or AN3CA cells upon release from CDK1i into AURKBi or nocodazole, indicating a strong mitotic progression regulatory capacity (Figs. 4 and 5; Supplementary Figs. S14A, S18A, and S20). The majority of the HEC1B cells either (i) eventually demonstrated a decreased percentage of mitotic cells upon release into AURKBi, or (ii) maintained the arrest upon release into nocodazole, especially at the higher dose (Figs. 4 and 5; Supplementary Figs. S14, S15, S18–S21). Finally, the HEC1B cells ultimately survived (i) traversing or possibly exiting mitosis, potentially as 4N cells, in the setting of AURKBi, and (ii) the prolonged arrest induced by nocodazole, both as indicated by the lack of or very limited induction of apoptosis by either the AURKBi or nocodazole ± initial CDK1i synchronization and release (Figs. 3D, 4E, 4F, 5E, and 5F; Supplementary Fig. S22A and S22B). This increased survival could be due in part to a stronger anti-apoptotic response in HEC1B cells, among many possibilities (64, 65). Determining how cells with a strong mitotic progression regulatory capacity such as HEC1B cells are (i) able to survive a prolonged mitotic arrest, (ii) eventually able to complete and possibly exit mitosis after a prolonged arrest, and (iii) able to exit mitosis in some cases as 4N cells and survive, will all be important future areas of investigation as they may be mechanisms of resistance to current mitosis targeting therapies in ECs and may represent rational future therapeutic targets (54, 57).

Second, our findings suggest an additional method for molecular profiling and stratification of ECs. Currently there are four EC molecular profiles based on targeted genomic sequencing panels, which may lead to categorization of ECs for later treatment based on mismatch repair or copy number status, or potentially for more aggressive treatment schedules based on *TP53* status given recent findings (1, 3, 4, 66). This molecular and therapeutic stratification is being done with a very limited understanding of the biologic contribution of these or other detected genomic alterations to EC therapeutic response. Based on our findings here linking EC cell cycle regulatory proficiency and deficiency, basal tumor cell transcriptomic states, and cell cycle targeted therapeutic vulnerabilities, we would also consider adding baseline transcriptional profiling to current EC molecular profiling. Specifically, we would assess transcriptomic profiles for misregulation of (i) the RB pathway, or (ii) mitotic spindle organization, the spindle assembly checkpoint, or possibly other mitotic progression regulatory pathways. We could then add (i) intact RB regulation or misregulated RB, especially in EC cells with no *RB1* mutations and expressing RB protein, or (ii) mitotic progression regulatory proficient or deficient to the current EC molecular subtypes, which may indicate vulnerability to CDK4/6is or AURKBis respectively (Figs. 2H, 2I, and 3G). These transcriptomic profiles would achieve additional biologic mechanism-based stratification that cannot be accomplished with current genomic profiling. Mutations or copy number changes in *RB1* or alterations in other genes which might alter RB function may be detected by genomic sequencing, but RB function can only be assessed transcriptionally or in live cell functional assays (11). Defects in regulation of mitotic progression can only be assessed transcriptionally or functionally. Extensive future baseline transcriptional profiling of additional EC models and of patient samples with known CDK4/6i or AURKBi response status will be needed to validate this type of RB and mitotic progression stratification and refine gene lists to be expression profiled. Similar studies could be done for other mechanism/therapy pairings. However, based on these findings, it may be possible to someday have an expression-based molecular profile for ECs similar to currently available expression profiling used for breast cancer stratification (67).

Third, our findings highlight the importance of performing matched functional, sensitivity, and transcriptomic analyses on genomically characterized EC models. Currently, IHC and genomic profiling are used to assess biology and possible therapeutic vulnerabilities of ECs in the clinic (1). For example, IHC is often used alone to determine *TP53* status (4). Our IHC on EC models matched the *TP53* genomic status for all models (Table 1); however, the combined IHC, genomic, and functional *TP53* status in our models did not correspond to sensitivity to any cell cycle kinase targeted therapies, which was unexpected given the known roles of p53 in cell cycle regulation (Figs. 2 and 3; refs. 8–10). Rather, RB regulatory and transcriptional status corresponded to CDK4/6i sensitivity, and mitotic progression regulatory functional and transcriptomic status corresponded to AURKBi sensitivity. Functional profiling of p53 and RB had not been done to this extent in EC before. Further mechanistic work will be necessary to understand the exact mechanism(s) of RB misregulation or mitotic progression regulation dysfunction in different ECs. However, these results highlight how novel therapeutic vulnerabilities can be identified and biologic insights made in EC through functionally and transcriptomically profiling genomically characterized EC models for proficiency or deficiency in basic cellular and molecular pathways such as cell cycle regulation, DNA damage repair, and beyond.

Additionally, these results have implications for current and emerging clinical trials. Specifically, a recent clinical trial with the CDK4/6i Abemaciclib in EC suggested that *TP53* mutations in the tumor may predict lack of response (15). In contrast, our results suggest that *TP53* mutant, *RB1* WT tumors with intact RB regulation can respond to CDK4/6i and that future CDK4/6i trials in EC could include *TP53* mutant tumors which may include serous or other aggressive histologies thereby expanding the patient cohort. For example, model UPSC-A was generated from a *TP53* mutant serous EC, however, RB regulation is intact in this model and the model is sensitive to CDK4/6 inhibition (Figs. 1 and 2). This patient received multiple therapies but never a CDK4/6i (Supplementary Table S1). It is possible given the RB regulatory status that this patient may have benefited from receiving a CDK4/6i. Given our findings, for CDK4/6i trials in EC going forward, RB regulatory status based on transcriptomic profiling of pre-treatment tumor tissue could be utilized as one of the inclusion criteria instead of histology or *TP53* genomic status. This will require validation by further genomic, transcriptomic, sensitivity, and functional assessment of RB and CDK4/6is in additional EC models of all histologic subtypes and genomic backgrounds.

Finally, these results have clinical implications for patients with more aggressive serous and high-grade endometrioid histologies. Serous, high-grade endometrioid, and carcinosarcoma ECs often present at a more advanced stage or recur rapidly, and there is limited biologic understanding of these aggressive tumors and thus limited targeted therapeutic options (1). With the exception of model EMCA-A, all of our models represent *TP53* mutant ECs, with several among these more aggressive EC subtypes (Table 1; Supplementary Table S1; refs. 36–42). Here we provide biologic insight into an unexpected proficiency of RB regulation in a subset of these tumors and a new targetable defect in the regulation of mitotic progression in another subset of these tumors, thereby offering two new testable candidate biomarkers and targeted therapies for these extremely difficult to treat EC subtypes. In this regard, more refined therapies targeting mitotic progression defects or at least more targeted delivery methods will be an important area of further investigation as current Aurora kinase targeted therapies reveal some toxicities in other tumor types (68, 69).

Taken together, our results suggest that current use of genomics or IHC to molecularly categorize ECs without understanding the biology of these tumors is ineffective. Through RB and p53 functional analysis paired with sensitivity and transcriptional testing in genomically characterized EC models, we have (i) defined new cell cycle regulatory proficiencies and deficiencies completely independent of *TP53* mutational status or histologic subtype to target in EC, (ii) defined two new mechanism-driven methods of molecularly categorizing ECs, and (iii) identified two new therapies with which to target the most aggressive EC subtypes. Continued characterization of the basic biology of ECs will be critical in further clinically and molecularly stratifying these tumors and also in continuing to identify new mechanism-driven therapeutic targets for this increasingly prevalent and deadly cancer type.

## Supplementary Material

Supplementary Methods This file contains Supplementary Materials and Methods along with accompanying Supplementary References.

Table S1 Supplementary Table S1

Figure S1 Supplementary Figure S1

Figure S2 Supplementary Figure S2

Figure S3 Supplementary Figure S3

Figure S4 Supplementary Figure S4

Figure S5 Supplementary Figure S5

Figure S6 Supplementary Figure S6

Figure S7 Supplementary Figure S7

Figure S8 Supplementary Figure S8

Figure S9 Supplementary Figure S9

Figure S10 Supplementary Figure S10

Figure S11 Supplementary Figure S11

Figure S12 Supplementary Figure S12

Figure S13 Supplementary Figure S13

Figure S14 Supplementary Figure S14

Figure S15 Supplementary Figure S15

Figure S16 Supplementary Figure S16

Figure S17 Supplementary Figure S17

Figure S18 Supplementary Figure S18

Figure S19 Supplementary Figure S19

Figure S20 Supplementary Figure S20

Figure S21 Supplementary Figure S21

Figure S22 Supplementary Figure S22
