# Supplementary material for: Functional Profiling of p53 and RB Cell Cycle Regulatory Proficiency Suggests Mechanism-Driven Molecular Stratification in Endometrial Carcinoma
Source: Cancer Res Commun. 2025 Apr 30;5(4):719–42. doi: 10.1158/2767-9764.CRC-24-0028 (PMC12042793; doi:10.1158/2767-9764.CRC-24-0028)
Supplement: Supplementary Methods — This file contains Supplementary Materials and Methods along with accompanying Supplementary References. [file crc-24-0028_supplementary_methods_suppsm.pdf]

SUPPLEMENTARY MATERIALS AND METHODS:

Bioluminescence imaging (BLI) for *in vivo* studies: For both the Abemaciclib and Barasertib *in vivo* studies described in Main Text Materials and Methods, BLI was used to follow all animals for tumor formation after cell injection to determine when to start drug treatments. The following BLI methods in quotation marks are provided verbatim from the Lurie Family Imaging Center and have been described previously (1). "Tumor growth was monitored at least weekly using the IVIS Spectrum In Vivo Imaging System (PerkinElmer) (1). Briefly, mice were injected subcutaneously with 75 mg/kg D-luciferin potassium salt (Promega E1605) in sterile PBS and anesthetized with 2% isoflurane in medical air (1). Serial bioluminescence images were acquired using the automated exposure set-up (1). The peak bioluminescence signal intensity within selected regions of interest (ROI) was quantified using Living Image Software (PerkinElmer) and expressed as photon flux (p/sec/cm^2/sr) (1). The ROI were placed on the abdomen of each animal."

Organoid generation and culture: Ascites and solid tumor samples were processed as follows for organoid generation. The ascites sample was spun at 1500 RPM for three minutes at room temperature in a table top centrifuge, the supernatant was aspirated, the cell pellet was resuspended and incubated in 1X Red Blood Cell lysis buffer (BioLegend Cat. #420301 is the 10X buffer) for five minutes at room temperature, the cells were spun at 1500 RPM for three minutes at room temperature in a table top centrifuge, the pellet was resuspended in Dulbecco's Modified Eagle Medium (DMEM) (Gibco Cat. #11965-092) with 10% FBS (Sigma-Aldrich Cat. #F2442) and 1% penicillin/streptomycin (P/S) (Gibco Cat. #15140-122), the cells were spun at 1500 RPM for three minutes at room temperature in a table top centrifuge, the media was aspirated, and then cells were either viably frozen and stored at -80°C or plated for organoid generation as described below. For solid tumors, upon receipt, a portion of solid tumor was snap frozen and stored at -80°C, and another portion was processed as follows. The solid tumor was first mechanically crushed using the flat edge of a syringe in a tissue culture plate in DMEM.

The crushed solid tumor was then poured onto a 100μm cell strainer filter (CELLTREAT Cat. #229485) placed on an open 50mL conical tube to filter large pieces of solid tissue, and any remaining solid tumor on the filter was then grated against the mesh filter with the flat edge of the syringe. The filter was rinsed with DMEM to allow for any further cells to move through the filter into the conical tube. The filter was then discarded and the filtered solid tumor cells in media were then spun at 1500 RPM for three minutes at room temperature in a table top centrifuge, the supernatant was aspirated, the cell pellet was resuspended and incubated in 1X Red Blood Cell lysis buffer for five minutes at room temperature, the cells were spun at 1500 RPM for three minutes at room temperature in a table top centrifuge, the pellet was resuspended in DMEM with 10% FBS and 1% P/S, the cells were spun at 1500 RPM for three minutes at room temperature in a table top centrifuge, the media was aspirated, and then cells were plated for organoid generation as described below.

Solid tumor or ascites cell pellets were resuspended in a volume of 50% Matrigel (Corning Cat. #356231) and 50% organoid media (described below) that was equal to the size of the cell pellet. This cell suspension was then plated in 48-well plates (CELLTREAT Cat. #229548) as 15μL domes in an appropriate number of wells for volume. Plates were incubated at 37°C for 10 minutes prior to adding 250μL of the below organoid media to each well. Organoids were grown in advanced DMEM/F-12 (Gibco Cat. #12634-028), supplemented with 1% P/S, 1% HEPES (Gibco Cat. # 15630080), 1X Glutamax (Gibco Cat. # 35050061), 500nM A83-01 (BioGems Cat. #9094360), 10μM SB202190 (BioGems Cat. #1523072), 10mM Nicotinamide (Sigma-Aldrich Cat. #N0636), 1X B-27 (Gibco Cat. #17504044), 1.25mM N-acetylcysteine (Sigma-Aldrich Cat. #A9165), 1μM Prostaglandin E2 (BioGems Cat. # 3632464), 10% Noggin and 10% R-Spondin1 conditioned media (for R-Spondin1 prepared from HA-R-Spondin1-FC 293T cells (R & D Systems Cat. # 3710-001-01) according to the manufacturer's protocol or for Noggin as described previously but with transfection of only 3μg of plasmid (2)), 10 ng/mL FGF-10 (Peprotech Cat. #100-26), 50 ng/mL EGF (Peprotech Cat. #100-15), and 10

ng/mL FGF2 (Peprotech Cat. #100-18B). After media was added to each well, plates were then incubated at 37°C in 5% CO_2. To establish organoids, media was changed every three days until large spheres formed. Organoids were then scraped from wells, spun at 3000 RPM for three minutes in a tabletop centrifuge, and the pellet was then incubated at 37°C in TrypLE (Gibco Cat. #12604-013) for 20 minutes with repeated shaking to digest to single cells. The single cells were spun at 1500 RPM for three minutes in a table top centrifuge, resuspended in a volume of 50% Matrigel/50% organoid media to allow for a 1:2 split, and then plated as 15μL domes in 48 well plates followed by incubation at 37°C for 10 minutes and then media addition. All organoid lines were then maintained at 37°C in 5% CO_2. Organoids were split and expanded for several passages as described above to allow for establishment of a line that could be expanded for experiments or frozen and thawed. Organoids were confirmed to be mycoplasma free by PCR, were expanded through the splitting procedure described above, and were then utilized at the earliest possible passage for all experiments.

Organoid and ARK cell line genomic sequencing: Genomic DNA from cell lines, parent tumors, and early passage organoids was generated using Qiagen's QIAamp DNA Mini Kit (Qiagen Cat. #51304). Low passage organoids were validated as matching the parent tumor tissue from which they were derived via STR (short tandem repeat) profiling of the organoid and parent tumor in the Center for Patient-Derived Models at Dana-Farber Cancer Institute. Genomic DNA from the organoids EMCA-A, UPSC-A, CS-A, and CS-B, and the cell lines ARK1 and ARK2 underwent whole exome sequencing at Novogene (Sacramento, CA) according to their protocols. Specifically, the following methods in quotation marks are provided by Novogene. "Agilent SureSelect Human All Exon V6 was utilized for library preparation. Sequencing occurred on the NovaSeq 6000 with a PE150 sequencing strategy."

The same *MSH2* and *MSH6* mutations detected in previous clinical genomic sequencing of the EMCA-A parent tumor were also detected in the EMCA-A organoid model by the whole exome sequencing described above, providing additional validation of this model (Figure S1A).

Additionally, the same *PIK3R1*, *TP53*, and *PPP2R1A* mutations detected in previous clinical genomic sequencing of the tumor from the original surgical resection for UPSC-A were also detected in the UPSC-A organoid model by the above whole exome sequencing, providing additional validation of this model (Figure S1A).

Whole Exome Sequencing Analysis: The below methods surrounded by quotation marks were provided verbatim by the Center for Patient-Derived Models at Dana-Farber Cancer Institute, and this language may represent a standard pipeline utilized there. Some of the methods have been previously reported and are updated below for this work (3).

"The human genome hg19 was used as a reference for samples EMCA-A, UPSC-A, CS-A, CS-B, ARK1, and ARK2. The raw reads were aligned using BWA-0.7.17 (RRID: SCR_010910) (4). These hg19 mapped bam (binary alignment map) files were coordinate sorted using Samtools-1.9 (RRID: SCR_002105) (5). Duplicate reads were marked and removed from the BAM files using Picard-2.23.3 *MarkDuplicates* tool (RRID: SCR_006525). Then additionally SetNmAndUqTags was used to calculate NM (Edit distance to the reference), MD (String encoding mismatched and deleted reference bases), and UQ (Phred likelihood of the segment, conditional on the mapping being correct). The de-duplicated file was then recalibrated using the gatk-4.1.4.1 *BaseRecalibrator* and *ApplyBQSR* tools (RRID: SCR_001876) (6). The recalibrated bam files were used to run the GATK tool *CollectF1R2Counts* and *LearnReadOrientationModel* to get the maximum likelihood estimates of artifact prior probabilities in the orientation bias mixture model filter. The variant calling was then performed on organoid or cell line samples using gatk-4.1.4.1/*Mutect2* pipeline with the default parameters and filters except for the following modifications: (i) 'af-of-alleles-not-in-resource' was set to 0, (ii) 'MateOnSameContigOrNoMappedMateReadFilter' was disabled, and (iii) a germline resource from the gnomAD database was included (3). The capture targets intervals used for Mutect2 was Agilent SureSelect DNA - SureSelect Human All Exon V6. The generated variant calls were further filtered using the *FilterMutectCalls* module of GATK. The

output from GetPileupSummaries and CalculateContamination modules of GATK were used in the *FilterMutectCalls* step. The final output in VCF (Variant Call File) format was annotated with Ensemble Variant Effect Predictor (ensembl-vep-96.0) (RRID: SCR_007931), and this annotated file was further converted into a maf file using vcf2maf-1.6.16 (7,8). The calls were additionally annotated with OncoKB dataset using oncokb-annotator (9). The variants in the maf file are filtered to include only non-synonymous call i.e. Nonstop_Mutation, Frame_Shift_Del, Frame_Shift_Ins, In_Frame_Del, In_Frame_Ins, Missense_Mutation, Nonsense_Mutation, Splice_Site, Splice_Region. The data is further filtered to remove variants that are not annotated as "PASS" from the mutect filter or isn't annotated as "Likely Oncogenic" or "Oncogenic". Further, the file was filtered to remove variants that are likely germline according to gnomAD (RRID: SCR_014964) (10) (variants with a population frequency > 0.001 were removed) but from the filtered list the variants are rescued which are seen in COSMIC v95 (RRID: SCR_002260) (11) at least twice. Variants are further filtered out for low VAF cases (<5%) and minimum depth of 10 reads, but the variants were rescued which are seen in COSMIC at least twice. The rescued variants are removed if the VAF of less than 1% even if they are seen in COSMIC at least twice."

Bulk RNA Sequencing: For cell lines, doubling times were calculated as described previously (12). For HEC1B and ARK1 models, cells were treated for approximately one doubling time (approximately 26 hours for ARK1 and 33.5 hours for HEC1B) with either 0.25μM Abemaciclib or media containing Dimethyl sulfoxide (DMSO) vehicle (ATCC Cat. #4-X-5) (used for analysis as batch 1). For organoids, a subset of the organoids were treated with media containing either 0.25μM Abemaciclib or media containing DMSO vehicle for 24 hours (used for analysis as batch 2). For additional cell lines and organoids to be used only for baseline transcriptomic comparisons, the cells were treated with media containing DMSO at 1:100,000 for 24 hours (used for analysis as batch 3). After treatments, cell lines were harvested and organoids were scraped from the plates, cells were pelleted, and all cell pellets were stored at -80°C until RNA

was prepared. RNA was prepared using Qiagen's RNEasy Mini kit (Qiagen Cat #74104) with on-column DNASE digest (Qiagen Cat. #79254) according to the manufacturer's protocol. RNA was snap frozen and sent to GENEWIZ (now part of Azenta Life Sciences, South Plainfield, NJ) for Standard RNA Sequencing. Each individual sample was sequenced in duplicate to provide technical replicates for validation. The following library preparation and sequencing methods in quotation marks are provided verbatim by GENEWIZ and may represent a standard pipeline used there.

"RNA samples were quantified using a Qubit 2.0 Fluorometer (Life Technologies, Carlsbad, CA, USA), and RNA integrity was checked using an Agilent TapeStation 4200 (Agilent Technologies, Palo Alto, CA, USA).

RNA sequencing libraries were prepared using the NEBNext Ultra II RNA Library Prep Kit for Illumina using the manufacturer's instructions (NEB, Ipswich, MA, USA). Briefly, mRNAs were initially enriched with Oligod(T) beads. Enriched mRNAs were fragmented for 15 minutes at 94°C. First-strand and second-strand cDNA were subsequently synthesized. cDNA fragments were end-repaired and adenylated at 3' ends, and universal adapters were ligated to cDNA fragments, followed by index addition and library enrichment by PCR with limited cycles. The sequencing library was validated on the Agilent TapeStation (Agilent Technologies, Palo Alto, CA, USA), and quantified by using a Qubit 2.0 Fluorometer (Invitrogen, Carlsbad, CA) as well as by quantitative PCR (KAPA Biosystems, Wilmington, MA, USA).

The sequencing libraries were clustered on a flow cell. After clustering, the flow cell was loaded on the Illumina instrument according to the manufacturer's instructions. The samples were sequenced using a 2x150bp Paired-End (PE) configuration, targeting 30 M reads/sample. The control software conducted image analysis and base calling. Raw sequence data (.bcl files) generated by the sequencer were converted into fastq files and de-multiplexed using Illumina's bcl2fastq 2.20 software. One mismatch was allowed for index sequence identification." Batch 1 utilized an Illumina HiSeq 4000 instrument, while batches 2 and 3 utilized an Illumina NovaSeq

6000 instrument.

RNA Sequencing Analysis:

RNA-sequencing analysis: RNA-sequencing (RNA-seq) analysis for all replicates of all samples from the bulk RNA sequencing above was performed using the VIPER pipeline with default parameters with individual VIPER runs for batches 1, 2, and 3 above (13). Sequenced reads were aligned to the hg19 reference genome assembly and gene counts were quantified using STAR aligner as part of VIPER (RRID: SCR_004463) (14).

Differential gene expression analysis: Differentially expressed (DE) genes were identified between the CDK4/6 inhibitor treatment and control DMSO samples for each cell line or organoid from batches 1 and 2 above using *DESeq2* (RRID: SCR_015687) (15). Technical batches were considered in the DESeq2 model to correct for batch effects. To remove genes with low expression, genes with less than or equal to 10 reads across all the samples were removed for each cell line or organoid before performing the differential expression analysis. Differentially expressed genes in the RB intact regulation group vs. RB misregulated group in baseline DMSO only treated samples from batch 3 were also identified using *DESeq2*, without controlling for batch effects.

Gene set enrichment analysis (GSEA): To identify core enrichment genes distinguishing RB intact regulation and misregulated samples in baseline (DMSO treated) samples from batch 3, we performed GSEA (RRID: SCR_003199) using the Hallmark and Reactome gene sets from MSigDB (16,17). Genes were ranked based on the Wald statistics calculated from the DESeq2 differential expression analysis. The analysis was conducted using the 'GSEA' function from the R package clusterProfiler (RRID: SCR_016884) (18).

Gene set variation analysis (GSVA): To estimate the pathway activity in baseline (DMSO treated) samples from batch 3, GSVA (RRID: SCR_021058) was performed using the Gene Ontology Biological Process, Hallmark, and Reactome gene sets from MSigDB (19). The input read counts were variance-stabilizing transformed using the *DESeq2* 'vst' function for the GSVA

analysis. Only genes with greater than or equal to 10 counts in two or more samples were included in the analysis. To compare the GSVA scores of Reactome Aberrant Regulation of Mitotic G1/S Transition in Cancer Due to RB1 Defects or Hallmark p53 Pathway gene sets between the CDK4/6 inhibitor sensitive and resistant models (sensitivity status based on functional profiling in Figures 2C and 2D), a Mann-Whitney U test was used. To compare the GSVA scores of Reactome Aberrant Regulation of Mitotic G1/S Transition in Cancer Due to RB1 Defects or GOBP Mitotic Spindle Organization gene sets between the Aurora kinase B inhibitor (AURKBi) sensitive and resistant models (sensitivity status based on functional profiling in Figure 3D), a Mann-Whitney U test was used. The 'ssgsea' algorithm from the R package *GSVA* was used for the GSVA analysis.

Correlation analysis: Pearson's correlation analysis was performed to investigate the relationship between RB regulation status and the sensitivity to CDK4/6 inhibition based on the flow cytometry results shown in Figures 1G, 1I, 2C, and 2D. RB regulation status, determined by G1 cell cycle arrest, was measured as the fold change in the percentage of G1 phase cells after treatment with CDK4/6 inhibitor compared to vehicle (DMSO). Sensitivity to a CDK4/6 inhibitor was quantified by the fold change in the percentage of senescence-associated β-galactosidase (SA-β-Gal) positive cells after treatment with CDK4/6 inhibitor compared to vehicle (DMSO). The analysis involved correlating these fold changes.

Additionally, the correlation between the ability of cells to accumulate as 4N bromodeoxyuridine (BrdU) for cell lines or 5-Ethynyl-2'-deoxyuridine (EdU) for organoids negative cells by combined BrdU-propidium iodide (PI)/EdU-PI analysis, or to accumulate with a 4N or greater than 4N DNA content by PI analysis alone post-Aurora kinase B (AURKB) inhibition and the sensitivity to AURKB inhibition was assessed using Pearson's correlation analysis based on the flow cytometry and sensitivity results shown in Figures 3D, 3E, 3F, S11A, S11B, and S11D. The fold change in the percentage of 4N BrdU/EdU negative cells (Figures

S11B and S11D), the percentage of 4N cells (Figures 3E and 3F), or the percentage of greater than 4N cells (Figure S11A) upon AURKB inhibitor treatment compared to vehicle (DMSO) was correlated with the sensitivity to AURKB inhibition, calculated by the area over the growth rate corrected dose-response curves (explained in the CellTiter-Glo analysis of survival after drug treatments section below) (Figure 3D). The greater than 4N analysis was done for cell lines only.

All correlation analyses utilized median values derived from two or three replicates per cell line or organoid. To conduct the analyses and generate scatter plots, R packages *ggpubr* (RRID: SCR_021139) and *ggplot2* (RRID: SCR_014601) were used.

Organoid and cell line immunohistochemistry (IHC) and tissue sample staining:
Organoids were scraped from an appropriate number of wells, washed in PBS, fixed in 10% neutral buffered formalin v/v (Fisher Cat. # 032-060) for 15 minutes, washed in PBS, pelleted, and then resuspended at high density in Histogel (Epredia Cat. #HG4000012). Histogel pellets were processed and embedded, unstained slides cut, and slides stained in the Brigham and Women's Hospital Specialized Histopathology Core. Cell lines were harvested and pelleted, cell pellets washed in PBS, fixed in 10% neutral buffered formalin v/v for 15 minutes, washed twice in PBS, and then taken to the Brigham and Women's Hospital Specialized Histopathology Core where cell pellets were processed, embedded, and unstained slides cut for various staining also in the core. Immunohistochemistry was performed on the Leica Bond III automated staining platform using the Leica Biosystems Refine Detection Kit (Leica DS9800). The p53 staining (anti-p53 antibody from Cell Signaling Technology, Cat. #48818, RRID: AB_2713958) was run at 1:100 dilution with citrate antigen retrieval. One slide also underwent hematoxylin and eosin staining for each line. Slides were analyzed for staining, and representative photos were taken at 40x or 100x magnification using an Olympus BX41TF microscope with an Olympus DP27 camera attached using Olympus cellSens software using optimized settings for each sample. For p53 IHC, we focused on nuclear staining. Staining patterns included heterogeneous for a mix of cells with p53 nuclear staining of varying strength or no nuclear staining, null for no

nuclear staining, strong diffuse nuclear staining for models in which almost all nuclei had strong nuclear staining, and strong diffuse nuclear staining with scattered negative nuclei for models that had predominantly strong nuclear staining in most cells but also rare scattered negative cells on the slide.

Residual solid tumor samples were collected from the vehicle or Barasertib treated animals in Figures 6B, 6C, and 6D or from the vehicle or Abemaciclib treated animals in Figures 6E, 6F, and 6G when possible. Tumors were fixed in 10% neutral buffered formalin v/v for at least one hour, and taken to the Brigham and Women's Hospital Specialized Histopathology Core where they were processed, embedded, and sectioned onto slides. For a subset of representative tumors from each study, one slide underwent hematoxylin and eosin staining; and these stained sections were then photographed for the representative photos for each study using an Olympus BX41TF microscope with an Olympus DP27 camera attached using Olympus cellSens software using optimized settings for each sample. Representative photos are shown in Figures 6C and 6F.

Cell line transfection: These methods have been described previously, and though similar, an updated version of the methods specific to this work are provided below (20). Cells were plated in a 6 cm dish on day 1. The following day cells were transfected with 10pmol of the appropriate siRNA using Lipofectamine RNAiMax (Invitrogen Cat. # 13778150) and Optimem (Gibco Cat. #31985070) according to the manufacturer's protocol (amounts of reagents were scaled up or down by surface area if different sized plates were transfected). Media was changed at the end of the day, and the transfection was repeated the following day. Cells were utilized for downstream applications 48-72 hours after the first transfection. siRNAs used included the following: siControl (Qiagen AllStars Negative Control Cat. #1027280), sip53-#5 (Horizon Discovery Cat. #D-003329-05-0002, GAGGUUGGCUCUGACUGUA), sip53-#7 (Horizon Discovery Cat. #D-003329-07-0002, GCACAGAGGAAGAGAAUCU), siRB-#5 (Horizon Discovery Cat. #D-003296-05-0002, GAAAGGACAUGUGAACUUA), siRB-#7 (Horizon

Discovery Cat. #D-003296-07-0002, GAAAUGACUUCUACUCGAA), siAURKA-#2 (Horizon Discovery Cat. #D-003545-02-0002, GAACUUACUUCUUGGAUCA), siAURKA-#5 (Horizon Discovery Cat. #D-003545-05-0002, CAAAUGCCCUGUCUUACUG), siAURKB-#7 (Horizon Discovery Cat. #D-003326-07-0002, CAGAAGAGCUGCACAUUUG), siAURKB-#8 (Horizon Discovery Cat. #D-003326-08-0002, CCAAACUGCUCAGGCAUAA), siPLK1-#5 (Horizon Discovery Cat. #D-003290-05-0002, CAACCAAAGUCGAAUAUGA), siPLK1-#7(Horizon Discovery Cat. #D-003290-07-0002, GAAGAUGUCCAUGGAAAUA), siCDK4-#5 (Horizon Discovery Cat. #D-003238-05-0002, GCAGCACUCUUAUCUACAU), siCDK4-#7 (Horizon Discovery Cat. #D-003238-07-0002, UCGAAAGCCUCUCUUCUGU), siCDK6-#5 (Horizon Discovery Cat. #D-003240-05-0002, GCAAAGACCUACUUCUGAA), siCDK6-#9 (Horizon Discovery Cat. #D-003240-09-0002, GGCCUUGCCCGCAUCUAUA), siCCNE1-#7 (Horizon Discovery Cat. #D-003213-07-0002, GGAGGUGUGUGAAGUCUAU), siCCNE1-#9 (Horizon Discovery Cat. #D-003213-09-0002, GUAUAUGGCGACACAAGAA), siBUB1B-#1 (Horizon Discovery Cat. #D-004101-01-0002, GGAAGAAGAUCUAGAUGUA), siBUB1B-#2 (Horizon Discovery Cat. #D-004101-02-0002, CAAGAUGGCUGUAUUGUUU), siCCNB1-#5 (Horizon Discovery Cat. #D-003206-05-0002, CAACAUUACCUGUCAUAUA), siCCNB1-#6 (Horizon Discovery Cat. #D-003206-06-0002, CCAAAUACCUGAUGGAACU), siCDC20-#13 (Horizon Discovery Cat. #D-003225-13-0002, GCAGAAACGGCUUCGAAAU), siCDC20-#27 (Horizon Discovery Cat. #D-003225-27-0002, CGGAAGACCUGCCGUUACA, siRRM2-#1 (Horizon Discovery Cat. #D-010379-01-0002, GCACUCUAAUGAAGCAAUA), siRRM2-#5 (Horizon Discovery Cat. #D-010379-05-0002, GAGUAGAGAACCCAUUUGA).

Colony Formation Assays: These methods have been described previously, and though similar, an updated version of the methods specific to this work are provided below (20). Cell lines were transfected as described above. Approximately 48 hours after the first transfection, cells were trypsinized and neutralized, counted, and then plated in 6 cm dishes at a suitable density for colony formation. Seven days later, media was removed, and cells were stained with

crystal violet staining solution (0.1% Crystal Violet in 20% EtOH). Plates were dried, and colonies were counted using a Celigo Image Cytometer (Nexcelom Bioscience, Lawrence, MA), Model #200-BFFL-5C, with standardized scanning and counting parameters. For each siRNA in an individual experiment, the cells were plated into three individual plates. The average number of colonies from the three plates was calculated and then divided by the average number of colonies from the control siRNA plates to obtain the percentage of colonies relative to the control. This was repeated three separate times, and the average of three experiments is shown in bar graphs.

CellTiter-Glo analysis of survival after drug treatments: These methods have been described previously, and though similar, an updated version of the methods specific to this work are provided below (20). For cell lines, the same number of cells were plated into each well of a 96-well plate (Corning Cat. # 3903) in the morning, and drug was added four to eight hours later. For organoids, organoids were digested with TrypLE (Gibco Cat. #12604-013) to single cells, and then mixed into a single cell suspension in media and Matrigel (Corning Cat. #356231) to have a final Matrigel concentration of 10-20%. The suspension was plated in domes in wells of a 96 well plate (Corning Cat. # 3903) such that there were equal numbers of cells in every well. The domes were allowed to solidify for 15 minutes at 37°C, and then media containing drug was added over the dome. For both cell lines and organoids, for each dose of drug, three wells of cells were plated. Dose curves for each different drug are listed below. Additionally, on the day of plating and treatment initiation, for both cell lines and organoids, four extra wells of cells per model were plated in a separate plate, and media with no drug was added. Those wells then immediately received the same volume of CellTiter-Glo (Promega Cat. #G7572) that the treated wells would eventually receive, and luminescence was read on a CLARIOstar Plus plate reader (BMG Labtech Ortenberg Germany) to provide an initial read for later growth rate correction. For the drug treated plates for both cell lines and organoids, the plates were incubated at 37°C in 5% CO_2 for five days, CellTiter-Glo was added, and

luminescence was read on the CLARIOstar Plus plate reader. The day five CellTiter-Glo reads along with the initial CellTiter-Glo reads of untreated cells taken on the day the cells were plated and treatment initiated were then used to generate a growth rate corrected dose-response curve for each model with each agent as described previously, since different cell lines and organoids grow at different rates at baseline (20,21). The area over the growth rate corrected dose-response curve was then calculated in GraphPad Prism, and this area over the curve represents sensitivity of the model to the agent. The larger the area, the greater the sensitivity, as described previously (20). The experiment was repeated twice for each model with each agent.

Drug dose curves:

Nutlin-3 (MedChemExpress Cat. # HY-50696): 0μM (media containing a volume of DMSO vehicle equivalent to the highest drug volume), 0.005μM, 0.05μM, 0.5μM, 1μM, 5μM, 10μM, 20μM.

PRIMA-1^{Met} (MedChemExpress Cat. # HY-19980): 0μM (media containing a volume of DMSO vehicle equivalent to the highest drug volume), 0.005μM, 0.05μM, 0.1μM, 0.5μM, 1μM, 5μM, 10μM.

Barasertib (MedChemExpress Cat. # HY-10127): 0μM (media containing a volume of DMSO vehicle equivalent to the highest drug volume), 0.001μM, 0.005μM, 0.01μM, 0.025μM, 0.05μM, 0.5μM, 1μM.

MK5108 (MedChemExpress Cat. # HY-13252): 0μM (media containing a volume of DMSO vehicle equivalent to the highest drug volume), 0.001μM, 0.005μM, 0.01μM, 0.025μM, 0.05μM, 0.5μM, 1μM.

Alisertib (MedChemExpress Cat. # HY-10971): 0μM (media containing a volume of DMSO vehicle equivalent to the highest drug volume), 0.001μM, 0.005μM, 0.01μM, 0.025μM, 0.05μM, 0.5μM, 1μM.

Onvansertib (MedChemExpress Cat. # HY-15828): 0μM (media containing a volume of DMSO

vehicle equivalent to the highest drug volume), 0.001μM, 0.005μM, 0.01μM, 0.025μM, 0.05μM, 0.5μM, 1μM.

Senescence-associated β-galactosidase activity flow cytometry: For cell lines, the doubling time for each cell line was calculated as described previously (12). In brief, a known number of cells were plated at low density in multiple wells of a six well plate. The cells were then trypsinized and viable cells were counted at 24 hour intervals using a hemacytometer with trypan blue (Gibco Cat. # 15250-061) used to mark dead cells. The doubling times were calculated on early passage cells using the formula described previously (12). Doubling times approximated using this method were as follows: ARK1 = 26 hours, ARK2 = 48.7 hours, HEC1B = 33.5 hours, AN3CA = 36 hours, KLE = 82.2 hours, Ishikawa = 39.5 hours, and RL95-2 = 27.1 hours.

As a pilot, to optimize dosing and timing, HEC1B cells were plated at low density, allowed to settle, and were then treated with media containing either 0.05 μM Abemaciclib, 0.25μM Abemaciclib, or DMSO vehicle. The cells were grown in this media for one, two, four, or five total doubling times with media being changed every other day. At the end of each doubling time, cells were trypsinized and neutralized, washed once in PBS, fixed in 2% paraformaldehyde (Electron Microscopy Sciences Cat. #15710-S diluted to 2% in PBS) for 15 minutes, washed in PBS, and stored at 4°C in Cell Staining Buffer (BioLegend Cat. #420201) until staining as described below. For the experiments shown in Figure 2C, cell lines were plated at low density, allowed to settle, and were then treated with media containing either 0.25μM Abemaciclib or DMSO vehicle. The cells were grown in this media for approximately four total doubling times with media being changed every other day. At the end of the fourth doubling time, cells were trypsinized and neutralized, washed once in PBS, fixed in 2% paraformaldehyde for 15 minutes, washed in PBS, and stored at 4°C in Cell Staining Buffer (BioLegend Cat. #420201) until staining. For organoids, organoids were digested to a single cell suspension and plated into domes as described above in the organoid generation and culture

section on day one. The organoids were allowed to recover and form small spheres for at least four days. On the fifth day, the media was changed to organoid media containing either 0.25μM Abemaciclib or DMSO vehicle; and the organoids were grown in this media for 10 total days with media being changed every other day. On the 10th day, the organoids were scraped from the plate, digested to a single cell suspension using TrypLE, washed in PBS, fixed in 2% paraformaldehyde for 15 minutes, washed in PBS, and stored at 4°C in Cell Staining Buffer until staining occurred. For staining, Invitrogen's CellEvent Senescence Green Flow Cytometry Assay Kit (Cat. #C10841) was used to detect senescence-associated β-galactosidase (SA-β-Gal) activity in treated cells and controls. An appropriate volume of Senescence Buffer containing the Senescence Green Probe was pre-warmed to 37°C in a water bath. 100μL of the buffer/probe mix was added to the cell pellets, the pellets were mixed, and then the suspensions were incubated at 37°C for one to two hours in a Fisherbrand 18L Low Temperature Incubator (Fisher Cat. #15-015-2632). Cells were then washed with Cell Staining Buffer and then analyzed on a BD LSR Fortessa Flow Cytometer. Please note, for Figure 2C, an initial subset of the cell lines, after the above SA-β-Gal staining, underwent an additional wash in Permeabilization Wash Buffer (BioLegend Cat. #421002), incubation in Permeabilization Wash Buffer, a wash in Cell Staining Buffer, and then were analyzed on the BD LSR Fortessa Flow Cytometer. The additional wash/incubation step yielded no extra results and was removed from the staining protocol for remaining experiments, and staining was done as above without any permeabilization. For all models with all staining formats, analysis was as follows. Cells positive for CellEvent Senescence Green signal were referred to as senescent or SA-β-gal positive cells, as green positivity indicates SA-β-gal activity which is a marker of senescent cells. The experiment was repeated three times for all models, and the flow cytometry data was gated in a standardized way on a standardized number of cells for each replicate for each model using FlowJo analysis software. Specifically, to gate the samples, the baseline gate was set on the

DMSO control for a model in one experiment individually, this gate was applied to all samples for that model for that experiment, and then the percentage of green or SA-β-gal positive cells was quantified for each sample for that model for that experiment.

In addition, photos were taken of all organoid models plated and then treated with DMSO vehicle or 0.25μM Abemaciclib for nine days exactly as described above at 4x magnification using a Laxco LMI3-PH1 microscope with a SebaCam camera attached using optimized settings for each sample. Photos were taken post-treatment for at least two independent replicates for each model. Representative photos of treated organoids are shown in Figure 2D.

Lysate preparation and western blots: These methods have been described previously, and an updated version of the methods specific to this work are provided below (20). Cell lines and organoids underwent various treatments for western blot analysis including 1) no treatment to study baseline protein levels; 2) transfection of cell lines with siRNAs as described above with harvest approximately 48 hours after the first transfection; 3) treatment for 24 hours with media containing either 0.25μM Abemaciclib or an equivalent volume of DMSO; 4) treatment for 24 hours with a dose curve of Abemaciclib including DMSO at a volume equivalent to the highest dose or 0.02μM, 0.1μM, 0.25μM, 0.5uM, or 1.0μM Abemaciclib; 5) treatment for 24 hours with media containing either 0.25μM Palbociclib (MedChemExpress Cat. #HY-50767) or an equivalent volume of DMSO; or 6) first treated with 9μM of the CDK1 inhibitor Ro-3306 (MedChemExpress Cat. #HY-12529) or media containing an equivalent volume of DMSO for 16 hours, washed five times with pre-warmed media, and then treated with either 0.1μM of the Aurora kinase B inhibitor Barasertib, 10ng/mL nocodazole (Sigma-Aldrich Cat. #SML-1665), 20ng/mL nocodazole, or an equivalent amount of DMSO for 24 hours. Cell lines were trypsinized and neutralized, washed with PBS, and stored at -80°C. Organoids were scraped from the plate, pelleted, incubated in Corning's Cell Recovery Solution (Cat. #354253) for 20 minutes at 4°C with end over end rotation to remove Matrigel, washed in PBS, pelleted, and

stored at -80°C. Pellets were lysed in ice cold NETN300 (300mM NaCl, 50mM Tris pH 8, 1mM EDTA, 0.5% NP40, 10% Glycerol) containing EDTA free Protease Inhibitor (Roche Cat. # 11873580001) for 15 minutes at 4°C with end over end rotation. Lysates were spun at maximum speed in a tabletop microcentrifuge for 15 minutes at 4°C. The supernatants were saved, and the concentrations were obtained using the Bio-Rad Protein Assay Kit (Bio-Rad Cat. # 5000001) on a spectrophotometer. Lysates were then normalized to be the same concentration by adding the appropriate volume of Laemmli Buffer with β-mercaptoethanol (Boston Bioproducts Cat. #BP-110NR supplemented with fresh β-mercaptoethanol for each use). For western blots, equal µg amounts of appropriate lysates were loaded into 4-12% Bis-Tris Gels (Invitrogen Cat. #NP0335BOX or #NP0336BOX) and run in MOPS running buffer (Invitrogen Cat. # NP0001). Proteins were then transferred to 0.45µm nitrocellulose membranes (Amersham Cat. #10600002) using a wet transfer box with wet Transfer Buffer (200mL methanol, 2.4g Tris (Invitrogen Cat. #15504-020), 14.4g Glycine, and 1g SDS all mixed in ddH_2O to a final volume of one liter). Membranes were blocked in 5% skim milk in TBS-T (Boston BioProducts Cat. # IBB-180X is 20X) for 20 minutes and then incubated overnight at 4°C in the appropriate primary antibody. Primary antibodies in this study included anti-p53 (Santa Cruz Biotechnology Cat. #sc-6243, RRID: AB_653753), anti-RB (Cell Signaling Technology Cat. #9309, RRID: AB_823629), anti-RB phosphorylated on Serine 807/811 (Cell Signaling Technology Cat. #8516, RRID: AB_11178658), anti-Phosphorylated CDC2 (Tyr15) (Cell Signaling Technology Cat. #4539, RRID: AB_560953), anti-CDC2 (Cell Signaling Technology Cat. #9116, RRID: AB_2074795), anti-GAPDH (BioLegend Cat. #649201, RRID: AB_10613283), anti-Aurora kinase A (Cell Signaling Technology Cat. #12100, RRID: AB_2797820), anti-Aurora kinase B (BioLegend Cat. #936201, RRID: AB_2832908), anti-PLK1 (BioLegend Cat. #627701, RRID: AB_439756), anti-CDK4 (Cell Signaling Technology Cat. #12790, RRID: AB_2631166), anti-CDK6 (Cell Signaling Technology Cat. #3136, RRID: AB_2229289), anti-Cyclin E1 (Cell Signaling Technology Cat.

#4129T, RRID: AB_2071200), anti-Bub1b (BubR1) (Bethyl Cat. #A300-386A, RRID: AB_386097), anti-Cyclin B1 (BioLegend Cat. #647901; RRID: AB_2072274), anti-CDC20 (BioLegend Cat. #934701; RRID: AB_2820217), anti-Cleaved PARP and full-length PARP (Cell Signaling Technology Cat. #9546T; RRID: AB_2160593), anti-RRM2 (Abnova Cat. #H00006241-M01A; RRID: AB_10662143), anti-Vinculin (Santa Cruz Biotechnology Cat. #sc-25336, RRID: AB_628438), and anti-Tubulin (Sigma-Aldrich Cat. #T9026, RRID: AB_477593). After overnight incubation in primary antibody, western blots were washed with TBS-T and then incubated for 45 minutes at room temperature in secondary antibody. Secondary antibodies included HRP conjugated anti-rabbit IgG, anti-mouse IgG, and anti-rat IgG (Cell Signaling Technology Cat. #s 7074, RRID: AB_2099233; 7076, RRID: AB_330924; and 7077, RRID: AB_10694715 respectively). Western blots were washed with TBS-T and then developed using BioLegend's plus or premium ECL (BioLegend Cat. #s 426316 or 426319) and exposed to film (Fisher Cat. # NC9556985). Western blots were stripped using Abcam's Mild Stripping Buffer (10mL Tween 20, 15g glycine, 1g SDS in 1L ddH$_2$0 at pH 2.2) for 30 minutes with rotation at room temperature, followed by three 10 minute washes in TBS-T, followed by blocking in 5% skim milk in TBS-T. Stripping allowed for re-incubation of membranes with different primary antibodies as needed and as indicated in Figure legends. Developed films were scanned in a standardized way in grayscale on an Epson Perfection V550 Photo Scanner, Model #J252B.

Apoptosis flow cytometry: ARK1, HEC1B, or AN3CA cells were plated in a 6cm dish on day one. The next day media containing either 9µM of the CDK1 inhibitor Ro-3306 or media containing an equivalent volume of DMSO was added, and the cells were incubated in this media for 16 hours. At the 16 hour timepoint, cells were washed five times with 1mL of pre-warmed media, and media containing either 0.1µM of the Aurora kinase B inhibitor Barasertib, 10ng/mL nocodazole, 20ng/mL nocodazole, or an equivalent amount of DMSO was added for 24 hours. At the 24 hour timepoint, cells were trypsinized and neutralized, and then washed once in Cell Staining buffer (BioLegend Cat. # 420201). Cells were then incubated in Cell

Staining buffer containing Zombie NIR dye (BioLegend Cat. #423105) and Apotracker Green (BioLegend Cat. # 427401) both at 1:200 for 20 minutes at room temperature in the dark. Cells were washed in Cell Staining buffer, resuspended in Cell Staining buffer, and then immediately analyzed on a BD LSR Fortessa flow cytometer. The experiment was repeated three times for each treatment combination. Experiments were analyzed in a standardized way using FlowJo analysis software. The percentage of Zombie NIR/Apotracker Green double positive cells was then calculated across treatments and plotted in bar graphs.

SUPPLEMENTARY REFERENCES

1. Wang Y, Buck A, Grimaud M, Culhane AC, Kodangattil S, Razimbaud C*, et al.* Anti-CAIX BBzeta CAR4/8 T cells exhibit superior efficacy in a ccRCC mouse model. Mol Ther Oncolytics **2022**;24:385-99
2. Heijmans J, van Lidth de Jeude JF, Koo BK, Rosekrans SL, Wielenga MC, van de Wetering M*, et al.* ER stress causes rapid loss of intestinal epithelial stemness through activation of the unfolded protein response. Cell Rep **2013**;3:1128-39
3. Touat M, Li YY, Boynton AN, Spurr LF, Iorgulescu JB, Bohrson CL*, et al.* Mechanisms and therapeutic implications of hypermutation in gliomas. Nature **2020**;580:517-23
4. Li H, Durbin R. Fast and accurate short read alignment with Burrows-Wheeler transform. Bioinformatics **2009**;25:1754-60
5. Danecek P, Bonfield JK, Liddle J, Marshall J, Ohan V, Pollard MO*, et al.* Twelve years of SAMtools and BCFtools. Gigascience **2021**;10
6. McKenna A, Hanna M, Banks E, Sivachenko A, Cibulskis K, Kernytsky A*, et al.* The Genome Analysis Toolkit: a MapReduce framework for analyzing next-generation DNA sequencing data. Genome Res **2010**;20:1297-303
7. McLaren W, Gil L, Hunt SE, Riat HS, Ritchie GR, Thormann A*, et al.* The Ensembl

Variant Effect Predictor. Genome Biol **2016**;17:122

8. Kandoth C. mskcc/vcf2maf: vcf2maf v1.6.16. **2020**

9. Chakravarty D, Gao J, Phillips SM, Kundra R, Zhang H, Wang J*, et al.* OncoKB: A Precision Oncology Knowledge Base. JCO Precis Oncol **2017**;2017

10. Karczewski KJ, Francioli LC, Tiao G, Cummings BB, Alfoldi J, Wang Q*, et al.* The mutational constraint spectrum quantified from variation in 141,456 humans. Nature **2020**;581:434-43

11. Tate JG, Bamford S, Jubb HC, Sondka Z, Beare DM, Bindal N*, et al.* COSMIC: the Catalogue Of Somatic Mutations In Cancer. Nucleic Acids Res **2019**;47:D941-D7

12. Torres-Guzman R, Calsina B, Hermoso A, Baquero C, Alvarez B, Amat J*, et al.* Preclinical characterization of abemaciclib in hormone receptor positive breast cancer. Oncotarget **2017**;8:69493-507

13. Cornwell M, Vangala M, Taing L, Herbert Z, Koster J, Li B*, et al.* VIPER: Visualization Pipeline for RNA-seq, a Snakemake workflow for efficient and complete RNA-seq analysis. BMC Bioinformatics **2018**;19:135

14. Dobin A, Davis CA, Schlesinger F, Drenkow J, Zaleski C, Jha S*, et al.* STAR: ultrafast universal RNA-seq aligner. Bioinformatics **2013**;29:15-21

15. Love MI, Huber W, Anders S. Moderated estimation of fold change and dispersion for RNA-seq data with DESeq2. Genome Biol **2014**;15:550

16. Subramanian A, Tamayo P, Mootha VK, Mukherjee S, Ebert BL, Gillette MA*, et al.* Gene set enrichment analysis: a knowledge-based approach for interpreting genome-wide expression profiles. Proc Natl Acad Sci U S A **2005**;102:15545-50

17. Mootha VK, Lindgren CM, Eriksson KF, Subramanian A, Sihag S, Lehar J*, et al.* PGC-1alpha-responsive genes involved in oxidative phosphorylation are coordinately downregulated in human diabetes. Nat Genet **2003**;34:267-73

18. Wu T, Hu E, Xu S, Chen M, Guo P, Dai Z*, et al.* clusterProfiler 4.0: A universal

enrichment tool for interpreting omics data. Innovation (Camb) **2021**;2:100141

19. Hanzelmann S, Castelo R, Guinney J. GSVA: gene set variation analysis for microarray and RNA-seq data. BMC Bioinformatics **2013**;14:7

20. Yang Z, Mogre S, He R, Berdan EL, Ho Sui SJ, Hill SJ. The ORFIUS complex regulates ORC2 localization at replication origins. NAR Cancer **2024**;6:zcae003

21. Hafner M, Niepel M, Chung M, Sorger PK. Growth rate inhibition metrics correct for confounders in measuring sensitivity to cancer drugs. Nat Methods **2016**;13:521-7
