# Supplementary material for: Functional Profiling of p53 and RB Cell Cycle Regulatory Proficiency Suggests Mechanism-Driven Molecular Stratification in Endometrial Carcinoma
Source: Cancer Res Commun. 2025 Apr 30;5(4):719–42. doi: 10.1158/2767-9764.CRC-24-0028 (PMC12042793; doi:10.1158/2767-9764.CRC-24-0028)
Supplement: Table S1 — Supplementary Table S1 [file crc-24-0028_table_s1_suppst1.pdf]

Table S1. Histology and treatment status of parent tumors utilized for organoid generation.
EMCA=endometrioid carcinoma, UPSC=uterine papillary serous carcinoma, CS=carcinosarcoma

Organoid Name	Site of parent tissue	Histologic Diagnosis	Treatment status
EMCA-A	Uterine mass	Grade 1-2 Endometrioid Endometrial Carcinoma	Untreated
UPSC-A	Recurrent ascites	Recurrent high-grade serous carcinoma of likely endometrial origin, based on anatomic distribution of tumor, WT1 negativity by immunohistochemistry, and clinical genomic sequencing identification of *ERBB2* amplification, *TP53* mutation, *PIK3R1* mutation, and *PPP2R1A* mutation all in the tumor from the original surgical resection. *See note.	Many treatments including carboplatin/paclitaxel, carboplatin/doxorubicin, pembrolizumab/lenvatinib, adavosertib
CS-A	Uterine mass	Endometrial Carcinosarcoma	Untreated
CS-B	Uterine mass	Endometrial Carcinosarcoma	Untreated

*NOTE: For UPSC-A, clinical validation of a recurrent ascites sample as metastatic carcinoma, consistent with spread from the patient's known Müllerian primary was also performed. The same *PIK3R1*, *TP53*, and *PPP2R1A* mutations identified in the tumor in the original surgical specimen are also identified in the organoid model used in this work. This group of mutations along with *ERBB2* amplification is common in uterine papillary serous carcinoma (Cancer Genome Atlas Research Network; Kandoth C, Schultz N, Cherniack AD, Akbani R, Liu Y, et al. Integrated genomic characterization of endometrial carcinoma. Nature 2013;497:67-73).
