# Supplementary material for: Functional Profiling of p53 and RB Cell Cycle Regulatory Proficiency Suggests Mechanism-Driven Molecular Stratification in Endometrial Carcinoma
Source: Cancer Res Commun. 2025 Apr 30;5(4):719–42. doi: 10.1158/2767-9764.CRC-24-0028 (PMC12042793; doi:10.1158/2767-9764.CRC-24-0028)
Supplement: Figure S1 — Supplementary Figure S1 [file crc-24-0028_figure_s1_suppsf1.pdf]

Figure S1

A

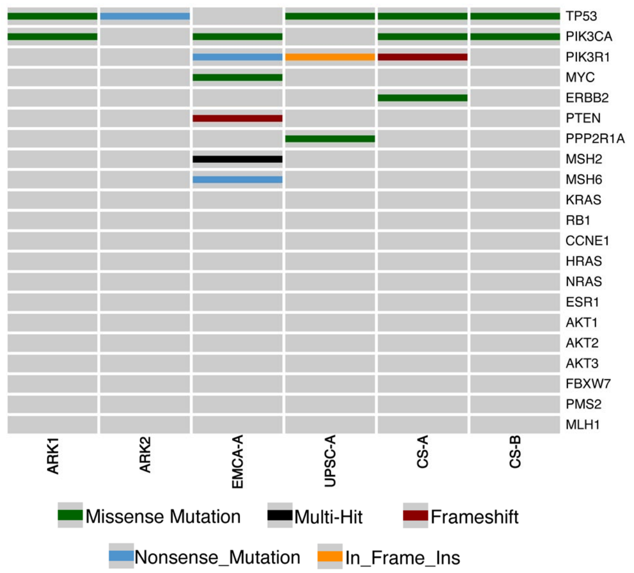

B

Organoid/Cell Line	*TP53* Mutation Status	p53 Amino Acid and DNA alterations
**ARK1**	Missense	p.R248W (c.742C>T)
**ARK2**	Nonsense	p.Q165* (c.493C>T)
**EMCA-A**	Wild type	-
**UPSC-A**	Missense	p.R273C (c.817C>T)
**CS-A**	Missense	p.G245S (c.733G>A)
**CS-B**	Missense	p.D281E (c.843C>G)

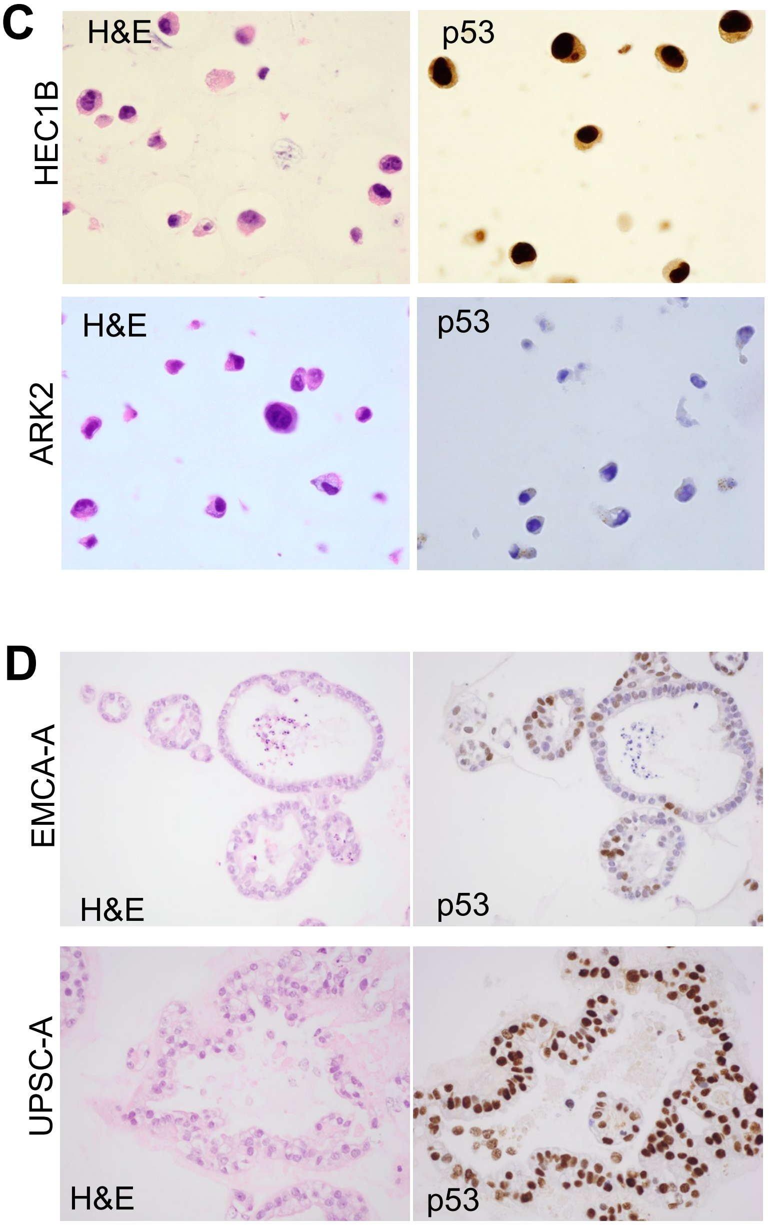

Figure S1. Whole exome sequencing data for organoids and two cell lines, and representative images. A) All organoid models and the two cell lines, ARK1 and ARK2, for which genomic sequencing data is not publicly available, underwent limited whole exome sequencing. Mutational status of key genes is shown with gene name on the right, model name on the bottom, and a key to mutation type below the panel. (EMCA=endometrioid carcinoma, UPSC=uterine papillary serous carcinoma, CS= carcinosarcoma, In_Frame_Ins=In frame insertion). **B)** *TP53* mutations detected in the limited whole exome sequencing data for all organoids and two cell lines are shown. Amino acid alterations (p.) and loci for *TP53* mutations on the coding sequence (c.) are shown in the right column. **C)** Cell lines and **D)** organoid lines underwent immunohistochemical staining for p53. Representative images of HEC1B and ARK2 cell lines stained for p53 (right) or for hematoxylin and eosin (H&E) (left) are shown in C. Representative photos of immunohistochemical staining of organoid lines EMCA-A and UPSC-A for p53 (right) or for hematoxylin and eosin (H&E) stain as a control (left) are shown in D.
