# Supplementary material for: Functional Profiling of p53 and RB Cell Cycle Regulatory Proficiency Suggests Mechanism-Driven Molecular Stratification in Endometrial Carcinoma
Source: Cancer Res Commun. 2025 Apr 30;5(4):719–42. doi: 10.1158/2767-9764.CRC-24-0028 (PMC12042793; doi:10.1158/2767-9764.CRC-24-0028)
Supplement: Figure S2 — Supplementary Figure S2 [file crc-24-0028_figure_s2_suppsf2.pdf]

Figure S2

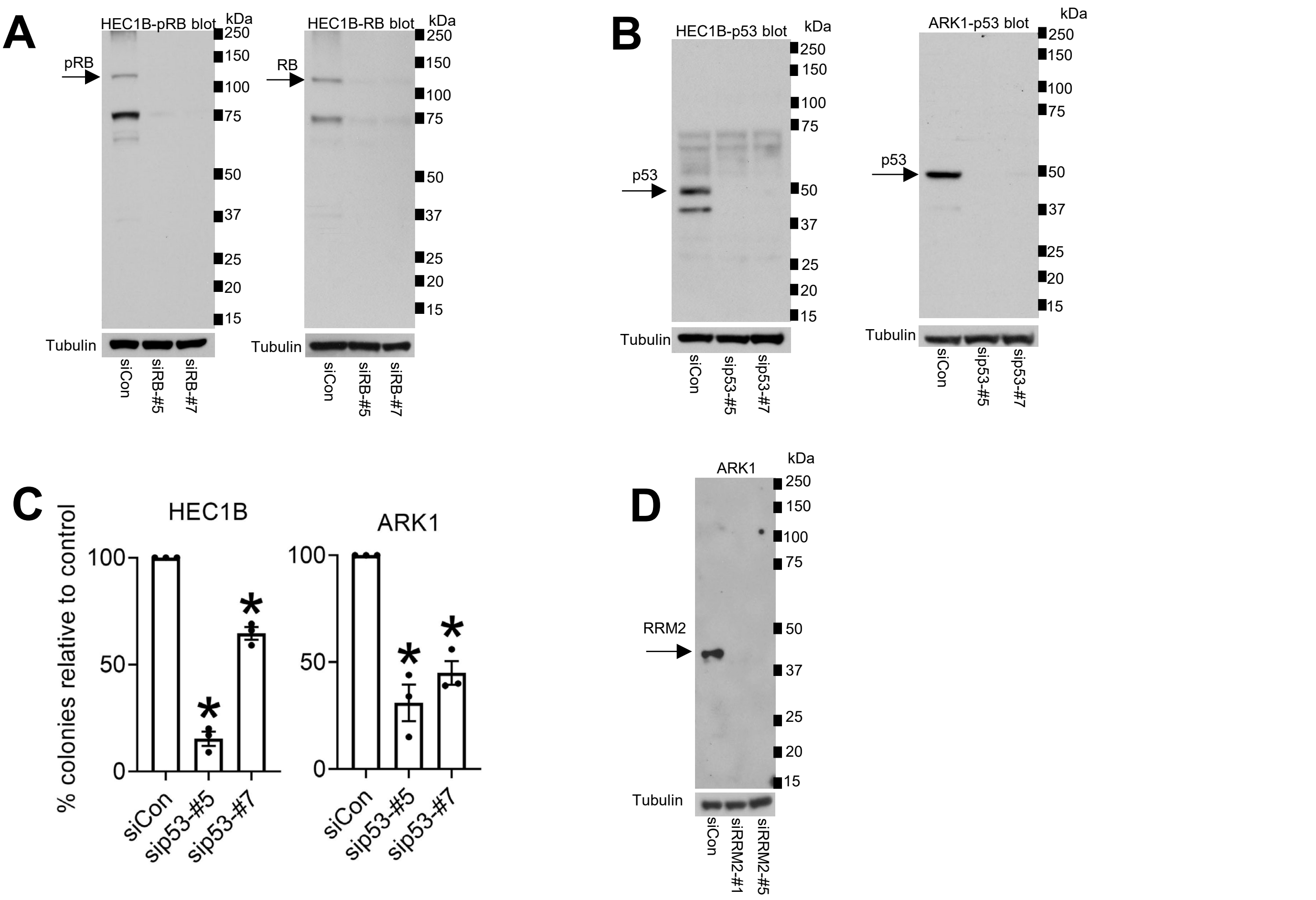

Figure S2. Western blot validation data. A) HEC1B cells were transfected with a control siRNA (siCon) or two RB-specific siRNAs (siRB-#5 and siRB-#7). Cell lysates were analyzed by western blot. A membrane from one gel was first stained for RB phosphorylated on serine 807/811 (pRB) and then stripped and re-probed for tubulin (left). A second membrane from a different gel was first stained for RB and then stripped and re-probed for tubulin. The main RB isoform is indicated by an arrow. **B)** HEC1B and ARK1 cells were transfected with a control siRNA (siCon) or two p53-specific siRNAs (sip53-#5 and sip53-#7). Lysates from transfected cells were analyzed by western blot. In each case, the membrane was first stained for p53 and then stripped and re-probed for tubulin. The main p53 isoform is indicated by an arrow in each blot. **C)** HEC1B and ARK1 cells were transfected with siCon or two p53-specific siRNAs (sip53-#5 and sip53-#7) and plated for colony formation. The percentage of colonies compared to the control was calculated. Bar graphs represent the average percentage of colonies compared to the control from three experiments, and error bars represent standard error of the mean. *=$p<0.05$ by paired t-test and also by ordinary one-way ANOVA with Dunnett's multiple comparisons test compared to siCon. **D)** ARK1 cells were transfected with a control siRNA (siCon) or two RRM2-specific siRNAs (siRRM2-#1 and siRRM2-#5). Lysates from transfected cells were analyzed by western blot. Membranes were first stained for RRM2 and then stripped and re-probed for tubulin.
