# Supplementary material for: Functional Profiling of p53 and RB Cell Cycle Regulatory Proficiency Suggests Mechanism-Driven Molecular Stratification in Endometrial Carcinoma
Source: Cancer Res Commun. 2025 Apr 30;5(4):719–42. doi: 10.1158/2767-9764.CRC-24-0028 (PMC12042793; doi:10.1158/2767-9764.CRC-24-0028)
Supplement: Figure S3 — Supplementary Figure S3 [file crc-24-0028_figure_s3_suppsf3.pdf]

Figure S3

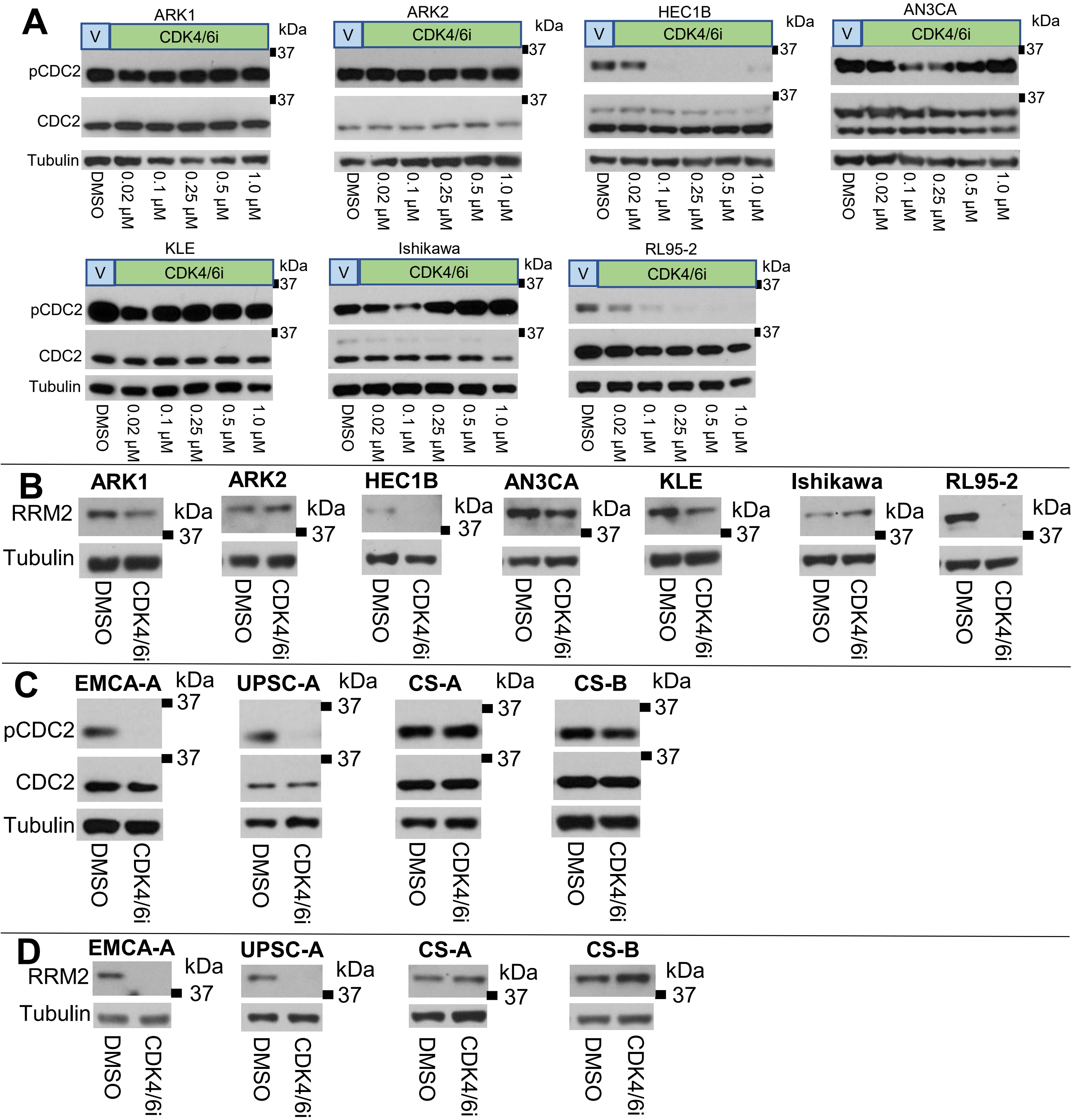

Figure S3. Only some cell lines and organoid models respond to CDK4/6 inhibition. A) Cell lines were treated with vehicle=V (DMSO) or a dose curve of the CDK4/6 inhibitor (CDK4/6i) Abemaciclib (doses indicated below blots) for 24 hours. Cell lysates were then analyzed by western blot. Membranes were cut between the 37 and 50kDa marker. The top portion of the blot was stained for tubulin. The bottom portion of the blot was first stained for CDC2 phosphorylated on Tyrosine 15 (pCDC2) and then stripped and re-probed for CDC2. CDC2 is also known as CDK1. **B)** Cell lines were treated with vehicle (DMSO) or 0.25µM of the CDK4/6i Abemaciclib for 24 hours and then analyzed by western blot. Membranes were first stained for RRM2 and then stripped and re-probed for tubulin. **C and D)** Organoids were treated with vehicle (DMSO) or 0.25µM of the CDK4/6i Abemaciclib for 24 hours and then analyzed by western blot. In C, membranes were first stained for pCDC2, stripped and re-probed for CDC2, and then stripped and re-probed for tubulin. In D, membranes were first stained for RRM2 and then stripped and re-probed for tubulin.
