# Supplementary material for: Functional Profiling of p53 and RB Cell Cycle Regulatory Proficiency Suggests Mechanism-Driven Molecular Stratification in Endometrial Carcinoma
Source: Cancer Res Commun. 2025 Apr 30;5(4):719–42. doi: 10.1158/2767-9764.CRC-24-0028 (PMC12042793; doi:10.1158/2767-9764.CRC-24-0028)
Supplement: Figure S4 — Supplementary Figure S4 [file crc-24-0028_figure_s4_suppsf4.pdf]

Figure S4

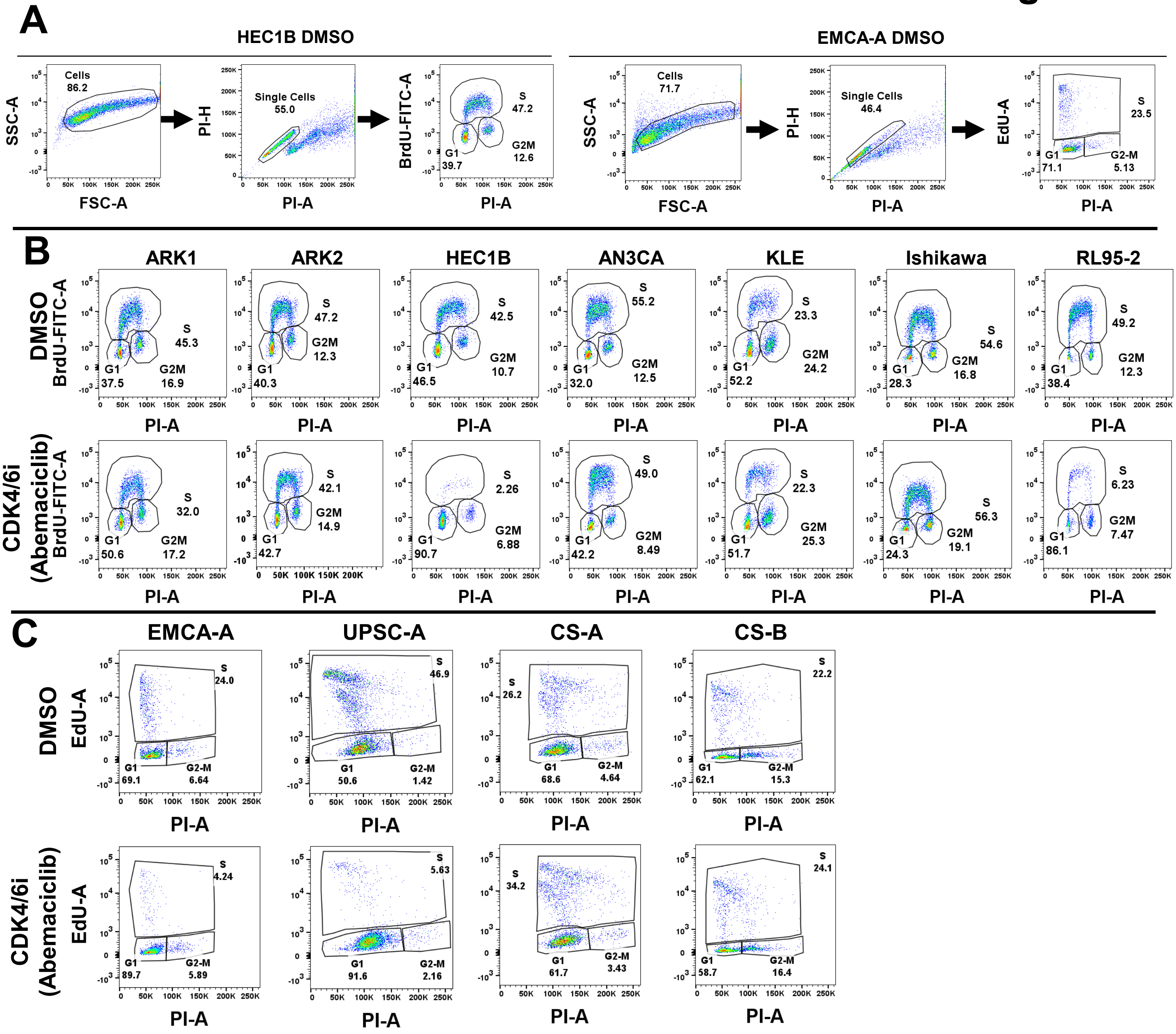

Figure S4. Gating strategies for CDK4/6 inhibitor cell cycle flow cytometry corresponding to Figures 1G and 1I. Cell or organoid lines were treated with vehicle (DMSO) or 0.25μM of the CDK4/6 inhibitor (CDK4/6i) Abemaciclib for 24 hours. The cell lines were pulsed with bromodeoxyuridine (BrdU) or the organoids were pulsed with 5-Ethynyl-2'-deoxyuridine (EdU). Cells were harvested, fixed, and stained with appropriate antibodies or chemicals as well as propidium iodide (PI) and then analyzed on a flow cytometer. Gating strategies from one replicate of some of the data in Figures 1G and 1I are shown here as described below. **A)** Shown here is a general gating strategy for one replicate of the DMSO treated HEC1B cell line on the left or the EMCA-A organoid line on the right. In each case, cells were first gated on the side scatter (SSC)/forward scatter (FSC) plot. From cells, singlets representing cells with 2N and 4N DNA content were gated on the PI-Height (H)/PI-Area (A) plot. Singlets were then plotted with PI on the X axis and either BrdU-FITC for cell lines or EdU for organoids on the Y axis. Cells which had a 2N DNA content and were negative for BrdU or EdU were gated as G1 phase. Cells which had a 4N DNA content and were negative for BrdU or EdU were gated as G2/M phase. Cells which were EdU or BrdU positive were gated and quantified as being in S phase. **B)** BrdU (Y-axis)/PI (X-axis) gating strategies are shown for all cell lines treated with vehicle (top) or CDK4/6i Abemaciclib (bottom) corresponding to Figure 1G. **C)** EdU (Y-axis)/PI (X-axis) gating strategies are shown for all organoid lines treated with vehicle (top) or CDK4/6i Abemaciclib (bottom) corresponding to Figure 1I.
