# Supplementary material for: Functional Profiling of p53 and RB Cell Cycle Regulatory Proficiency Suggests Mechanism-Driven Molecular Stratification in Endometrial Carcinoma
Source: Cancer Res Commun. 2025 Apr 30;5(4):719–42. doi: 10.1158/2767-9764.CRC-24-0028 (PMC12042793; doi:10.1158/2767-9764.CRC-24-0028)
Supplement: Figure S5 — Supplementary Figure S5 [file crc-24-0028_figure_s5_suppsf5.pdf]

Figure S5

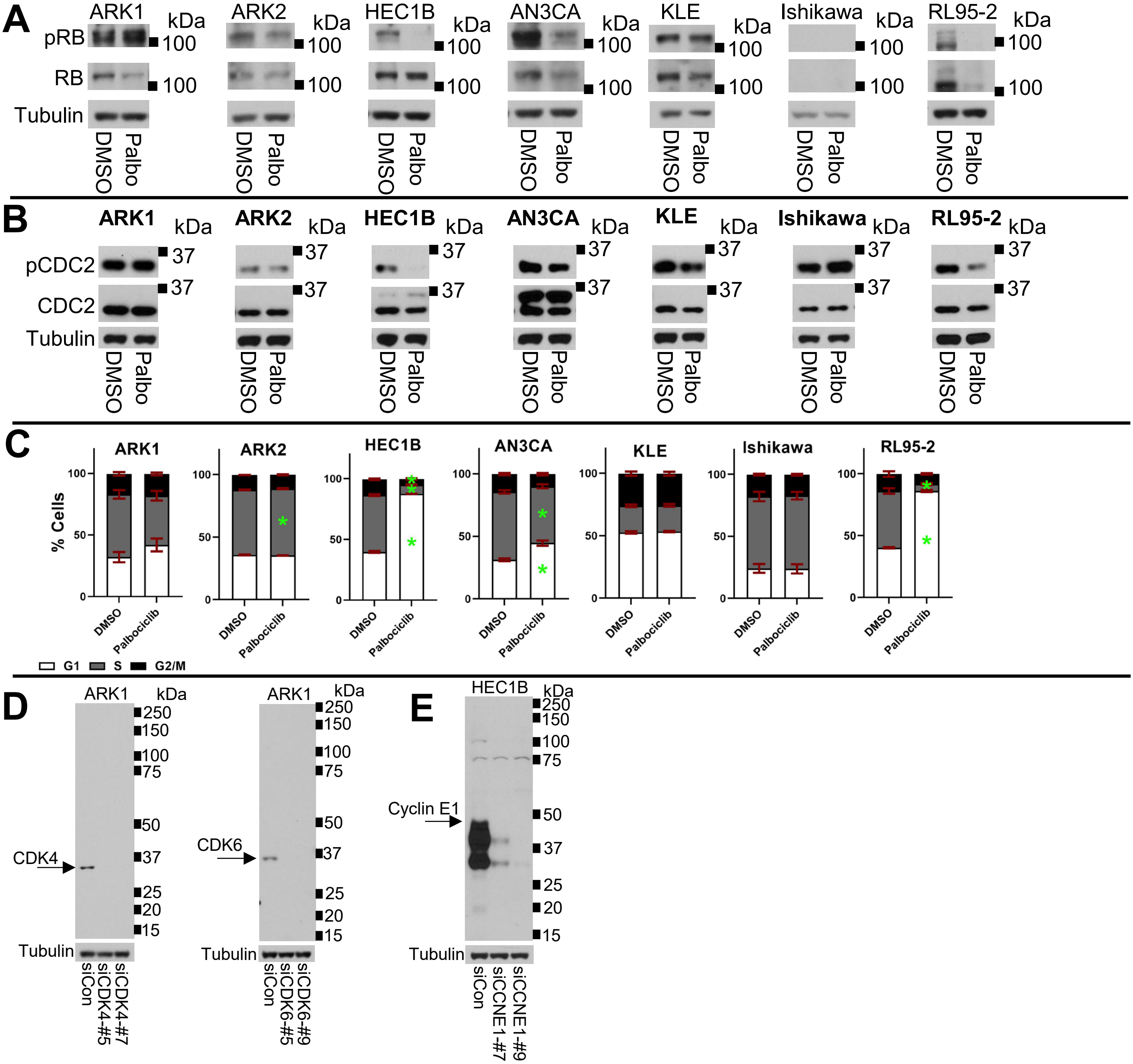
A
ARK1
ARK2
HEC1B
AN3CA
KLE
Ishikawa
RL95-2
kDa
pRB
RB
Tubulin
100
DMSO
Palbo
B
pCDC2
CDC2
37
C
% Cells
100
50
0
DMSO
Palbociclib
G1
S
G2/M
D
CDK4
CDK6
250
150
75
25
20
15
siCon
siCDK4-#5
siCDK4-#7
siCDK6-#5
siCDK6-#9
E
Cyclin E1
siCCNE1-#7
siCCNE1-#9

Figure S5. Cell lines respond similarly to Palbociclib and Abemaciclib. A, B, and C) Cell lines were treated with either 0.25μM of the CDK4/6 inhibitor Palbociclib (Palbo) or vehicle (DMSO) for 24 hours and then underwent western blot or flow cytometry analysis. For western blot analysis in A, equal μg amounts of the same lysates were loaded side-by-side on a gel, and the membrane was cut to allow the same lysates to be probed for different markers simultaneously. One membrane was first probed for RB and then stripped and re-probed for tubulin. The other membrane was probed for RB phosphorylated on serine 807 and 811 (pRB). In Panel B, lysates were loaded once on a gel, then the membrane was probed first for CDC2 phosphorylated on Tyrosine 15 (pCDC2), stripped and re-probed for CDC2, and then stripped and re-probed for tubulin. In C, control or palbociclib treated cells underwent bromodeoxyuridine/propidium iodide cell cycle flow cytometry profiling. For the bar graphs in C, bars represent the percent of cells in each different cell cycle phase from three independent replicates and error bars represent standard error of the mean. *=p<0.05 compared to DMSO for the specific cell cycle phase by an ordinary two-way ANOVA with Šídák's multiple comparisons test. If there is no *, then the comparison was not significant. The color code for the cell cycle phase is below one of the graphs. The gating strategy utilized was the same as that demonstrated for Abemaciclib in Supplementary Figure S4. **D)** ARK1 cells were transfected with a control siRNA (siCon) or two CDK4-specific siRNAs (siCDK4-#5 and siCDK4-#7) on the left or two CDK6-specific siRNAs (siCDK6-#5 and siCDK6-#9) on the right. Transfected cells were analyzed by western blot. The membrane was stained for CDK4 and then stripped and re-probed for tubulin (left), or the membrane was stained for CDK6 and then stripped and re-probed for tubulin (right). The main CDK4 or CDK6 isoform is indicated by an arrow in each blot. **E)** HEC1B cells were transfected with a control siRNA (siCon) or two CCNE1-specific siRNAs (siCCNE1-#7 and siCCNE1-#9). Transfected cells were analyzed by western blot. The membrane was stained for Cyclin E1 and then stripped and re-probed for tubulin. An arrow indicates the main Cyclin E1 isoform, and all bands below that represent low molecular weight Cyclin E1 isoforms.
