# Supplementary material for: Functional Profiling of p53 and RB Cell Cycle Regulatory Proficiency Suggests Mechanism-Driven Molecular Stratification in Endometrial Carcinoma
Source: Cancer Res Commun. 2025 Apr 30;5(4):719–42. doi: 10.1158/2767-9764.CRC-24-0028 (PMC12042793; doi:10.1158/2767-9764.CRC-24-0028)
Supplement: Figure S6 — Supplementary Figure S6 [file crc-24-0028_figure_s6_suppsf6.pdf]

Figure S6

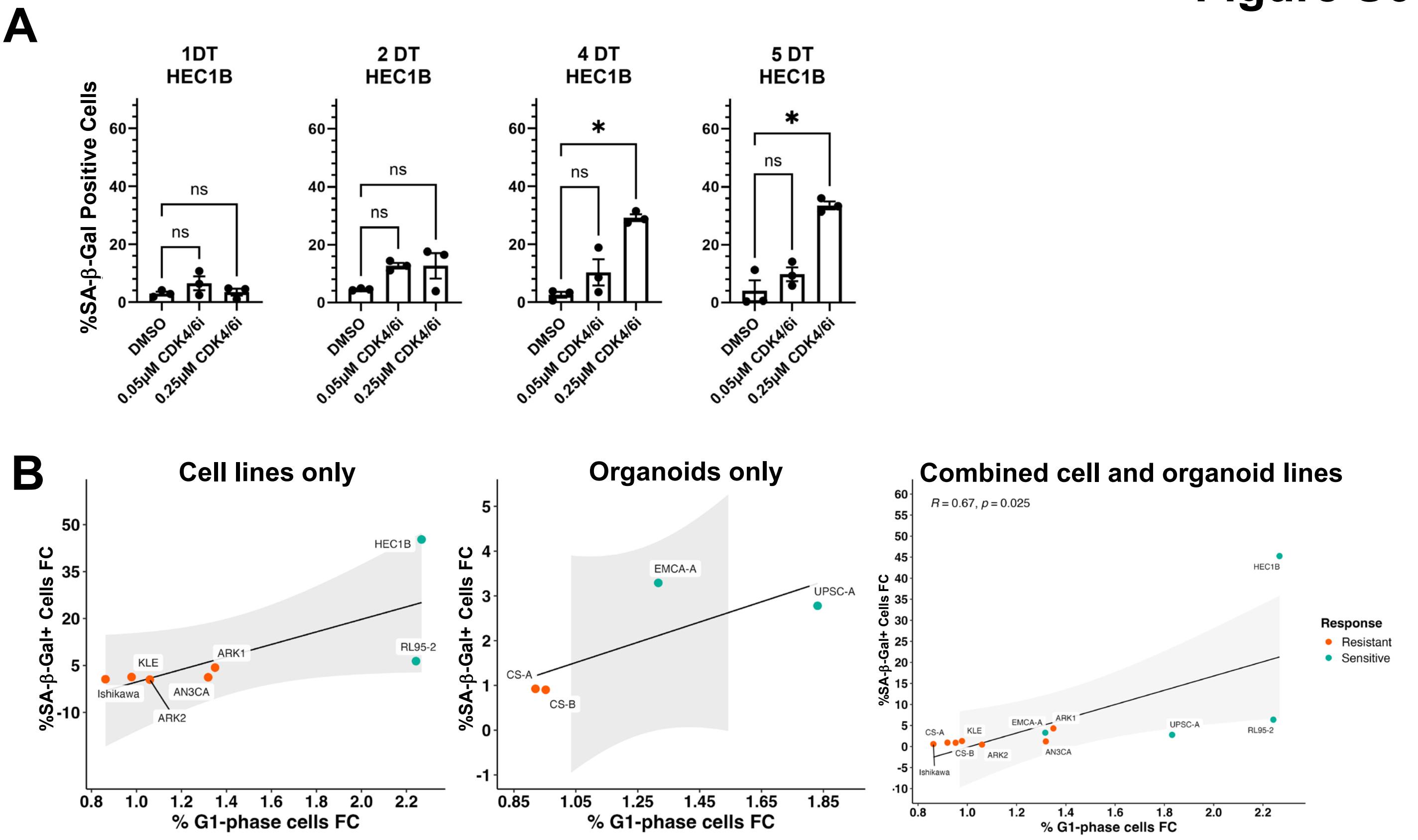

Figure S6. Dosing and timing optimization for CDK4/6 inhibitor senescence-associated β–galactosidase assay and correlation analysis. A) The doubling time was calculated for the HEC1B cell line which had shown a strong response to Abemaciclib by western blot and cell cycle profiling. HEC1B cells were then treated with media containing vehicle (DMSO) or 0.05 or 0.25 µM of the CDK4/6 inhibitor (CDK4/6i) Abemaciclib for one, two, four, or five doubling times (DTs) with media changes every 48 hours. Cells were harvested at appropriate timepoints. At the end of the timecourse all cells were stained for senescence-associated β-galactosidase (SA-β-Gal) activity using the CellEvent Senescence Green Flow Cytometry Assay Kit, and analyzed by flow cytometry. Cells positive for the SA-β-Gal activity detection probe were considered senescent and referred to as SA-β-Gal positive cells. The timecourse was repeated three times. The average percentage of SA-β-Gal positive cells from the replicates is shown in bar graphs for each timepoint with error bars representing standard error of the mean. p-values were calculated for each timepoint using an ordinary one-way ANOVA with Šídák's multiple comparisons test. *=p<0.05. ns=not significant. **B)** These scatter plots illustrate the correlation between the median fold change (FC) in the percentage of G1-phase cells following treatment with 0.25µM CDK4/6 inhibitor compared to vehicle (DMSO) (from Figures 1G and 1I) and the median FC in the percentage of SA-β-Gal positive cells after CDK4/6 inhibition compared to vehicle (DMSO) (from Figures 2C and 2D). The plots are shown for cell lines alone on the left, organoids alone in the middle, and all cell and organoid models combined on the right. The different model types are shown individually and also in the final combined analysis since the organoid models have higher baseline SA-β-Gal positivity than the cell lines. Thus, the overall SA-β-Gal positive fold change is smaller for each organoid making the models which are sensitive fall lower on the Y-axis in the combined model correlation plot on the right near some of the resistant cell lines. To illustrate that there is a difference between sensitive and resistant organoid models, we plotted the correlation analyses separated by the type of model on the left and in the middle, one each for the cell lines and organoids. These analyses show distinct clusters for resistant and sensitive models for both the organoids and the cell lines but are on different scales. We then plotted all models for one analysis on the far right. Statistics were calculated for all models combined as the number of models was small. The '*R*' value in the far-right figure for all models combined represents the correlation coefficient, indicating the strength and direction of the relationship, while '*p*' represents the p-value obtained from Pearson's correlation analysis. The grey shaded region in the graphs represents the 95% confidence interval for the black linear regression line in the graph. A color code for CDK4/6i sensitive and resistant models is shown to the right of the combined plot.
