# Supplementary material for: Functional Profiling of p53 and RB Cell Cycle Regulatory Proficiency Suggests Mechanism-Driven Molecular Stratification in Endometrial Carcinoma
Source: Cancer Res Commun. 2025 Apr 30;5(4):719–42. doi: 10.1158/2767-9764.CRC-24-0028 (PMC12042793; doi:10.1158/2767-9764.CRC-24-0028)
Supplement: Figure S7 — Supplementary Figure S7 [file crc-24-0028_figure_s7_suppsf7.pdf]

Figure S7

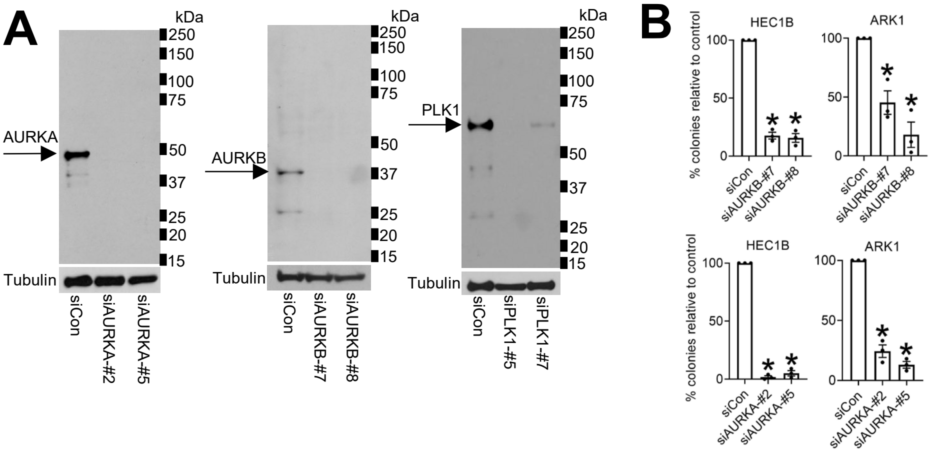

Figure S7. Mitotic kinase antibody validation and colony formation assays. A) ARK1 cells were transfected with a control siRNA (siCon) and either two Aurora kinase A (AURKA)-specific siRNAs (siAURKA-#2 and siAURKA-#5) on the left, two Aurora kinase B (AURKB) specific siRNAs (siAURKB-#7 and siAURKB-#8) in the middle, or two polo-like kinase 1 (PLK1)-specific siRNAs (siPLK1-#5 and siPLK1-#7) on the right. The cell lysates were then analyzed by western blot. On the left, the membrane was stained for AURKA and then stripped and re-probed for tubulin. In the middle, the membrane was stained for AURKB and then stripped and re-probed for tubulin. On the right, the membrane was stained for PLK1 and then stripped and re-probed for tubulin. **B)** HEC1B and ARK1 cells were transfected with siCon or two AURKB-specific siRNAs (siAURKB-#7 or siAURKB-#8) on top or two AURKA-specific siRNAs (siAURKA-#2 or siAURKA-#5) on the bottom. Cells were plated at a suitable density for colony formation, and the percentage of colonies relative to the control was then calculated after a standardized incubation time. The bar graphs represent the average of three independent experiments with error bars representing standard error of the mean. *= $p<0.05$ compared to siCon by paired t-test and also by ordinary one-way ANOVA with Dunnett’s multiple comparisons test.
