# Supplementary material for: Functional Profiling of p53 and RB Cell Cycle Regulatory Proficiency Suggests Mechanism-Driven Molecular Stratification in Endometrial Carcinoma
Source: Cancer Res Commun. 2025 Apr 30;5(4):719–42. doi: 10.1158/2767-9764.CRC-24-0028 (PMC12042793; doi:10.1158/2767-9764.CRC-24-0028)
Supplement: Figure S8 — Supplementary Figure S8 [file crc-24-0028_figure_s8_suppsf8.pdf]

Figure S8

Gating Strategy #1 for mitosis targeting drug BrdU/EdU-PI cell cycle flow cytometry experiments

PI only flow cytometry gating for DNA content

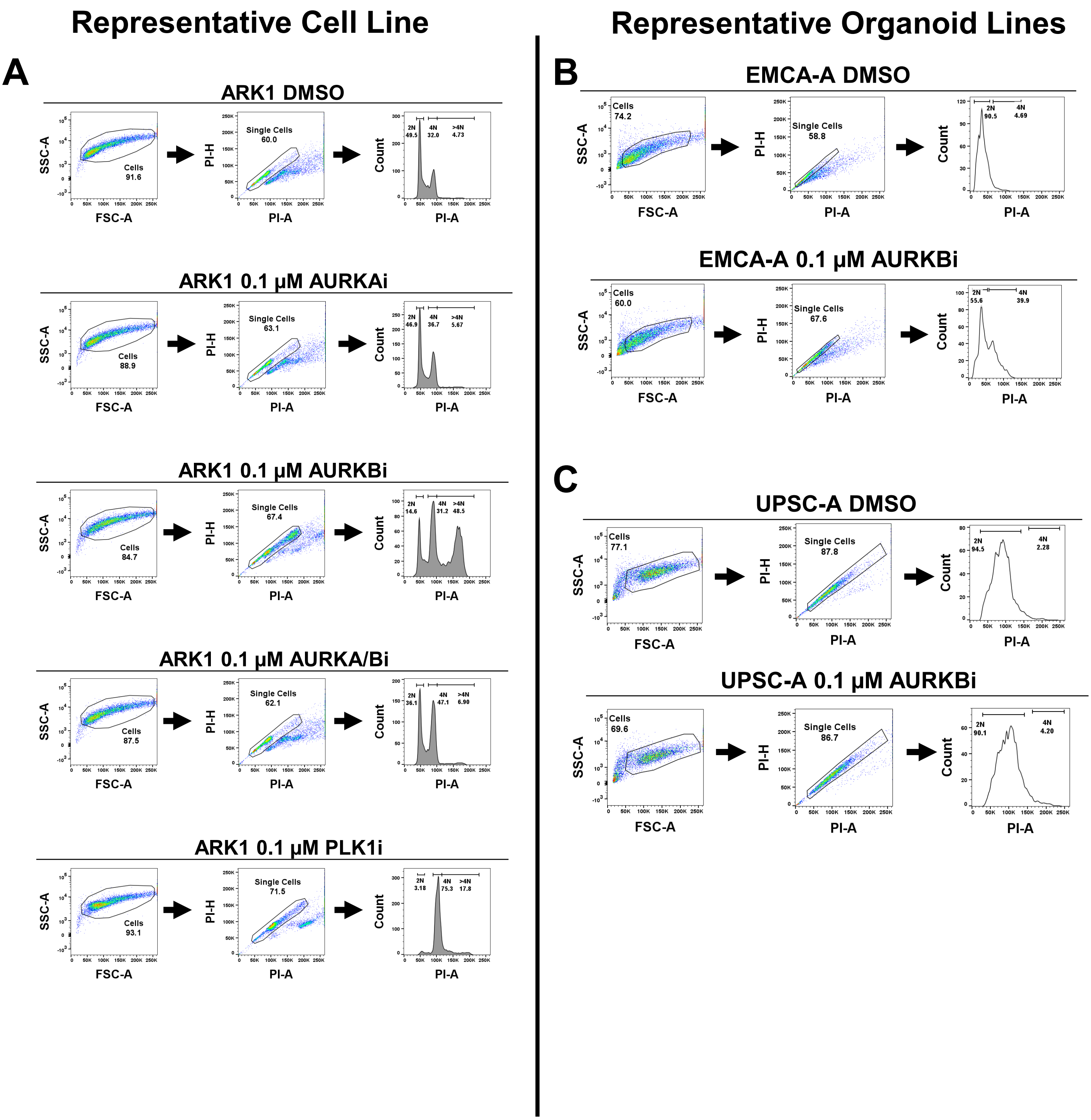

Figure S8. Endometrial carcinoma cells show varied DNA content changes in response to different mitosis targeting agents. A, B, and C) Shown here is the gating strategy for cell cycle flow cytometry analysis of cell lines or organoid lines for propidium iodide (PI) analysis alone corresponding to the data in Figures 3E, 3F, S9, and S11. Please note that for each cell or organoid line, the experiment detailed below was repeated three times, and the data from those three replicates was analyzed and represented in two ways with representative gating strategies performed on one of the replicates for the two analysis methods shown here and in Figure S9. Cell lines were treated with vehicle (DMSO) or 0.1 μM of the Aurora kinase A inhibitor MK5108 (AURKAi), the Aurora kinase B inhibitor Barasertib (AURKBi), the dual Aurora kinase A/B inhibitor (AURKA/Bi) Alisertib, or the PLK1 inhibitor onvansertib (PLK1i) for 24 hours. Organoid lines were treated with vehicle (DMSO) or 0.1μM AURKBi for 24 hours. Prior to harvest, the cell lines were pulsed with bromodeoxyuridine (BrdU) and the organoids were pulsed with 5-ethynyl-2'-deoxyuridine (EdU). Cells were then harvested, fixed, and stained with appropriate antibodies or chemicals as well as propidium iodide (PI) to mark total DNA content and then analyzed by flow cytometry.

In a subset of the cell lines but not the slower cycling organoid lines, the AURKBi induced a greater than 4N (>4N) DNA content population which it has been previously shown to do, and thus we analyzed this data in two ways to ensure no findings were missed. To take the >4N DNA content population into account we analyzed the PI data alone for DNA content, and to take S phase into account we separately analyzed the combined BrdU/EdU-PI data. Shown here is the gating strategy for the PI analysis alone on one of the replicates from a few representative models, and the BrdU/EdU-PI analysis on one of the replicates from a few representative models is shown in Figure S9. The gating strategy for ARK1 cells, which did exhibit a >4N DNA content population with AURKBi, is shown in Panel **A** in the left column for all four mitosis targeting drugs compared to the DMSO control. The PI gating strategy for EMCA-A organoids or UPSC-A organoids with DMSO and AURKBi is shown in the right column in panels **B** and **C** respectively.

In each case, cells were first gated on the side scatter (SSC)/forward scatter (FSC) plot shown on the left. From cells, single cells (singlets) were gated on the PI-Area (PI-A)/PI-Height (PI-H) plot to include 2N, 4N, and greater than 4N (>4N) DNA content if present. Singlets were then shown in a PI profile plot with PI on the X-axis and cell counts on the Y-axis to allow gating of 2N, 4N, or greater than 4N DNA content cells as shown.
