# Supplementary material for: Functional Profiling of p53 and RB Cell Cycle Regulatory Proficiency Suggests Mechanism-Driven Molecular Stratification in Endometrial Carcinoma
Source: Cancer Res Commun. 2025 Apr 30;5(4):719–42. doi: 10.1158/2767-9764.CRC-24-0028 (PMC12042793; doi:10.1158/2767-9764.CRC-24-0028)
Supplement: Figure S9 — Supplementary Figure S9 [file crc-24-0028_figure_s9_suppsf9.pdf]

Figure S9

Gating Strategy #2 for mitosis targeting drug BrdU/EdU-PI cell cycle flow cytometry experiments

Combined BrdU/EdU-PI gating to include S Phase Cells

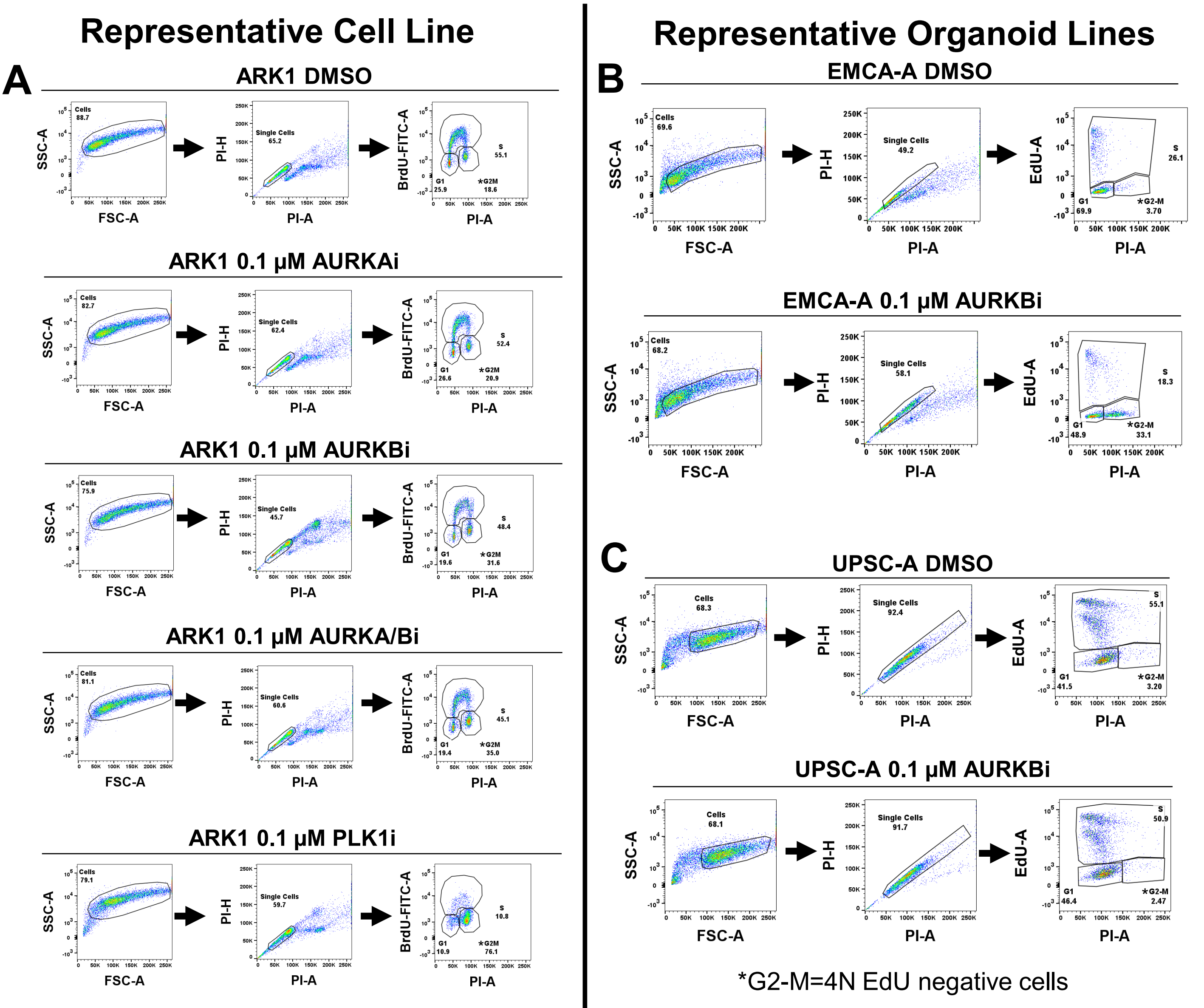

*G2M=4N BrdU negative cells

*G2-M=4N EdU negative cells

-Please note that for this Figure and the related Figure S11 only, because AURKBis and some other mitosis targeting drugs can potentially induce 4N G1 cells making 4N DNA content cells which are negative for BrdU or EdU difficult to classify, we are referring to the cells gated as "*G2M" or "*G2-M" in the above plots as 4N BrdU/EdU negative cells.

Figure S9. Endometrial carcinoma cells show varied cell cycle changes in response to different mitosis targeting agents. A, B, and C) Shown here is the gating strategy for combined bromodeoxyuridine (BrdU)/5-ethynyl-2'-deoxyuridine (EdU)-propidium iodide (PI) analysis corresponding to the data in Figures 3E, 3F, S8, and S11. Please note that for each cell or organoid line, the experiment was repeated three times, and the data from those three replicates was analyzed and represented in two ways with representative gating strategies for the two analysis methods shown here and in Figure S8.

Cell lines were treated with vehicle (DMSO) or 0.1 µM of the Aurora kinase A inhibitor MK5108 (AURKAi), the Aurora kinase B inhibitor Barasertib (AURKBi), the dual Aurora kinase A/B inhibitor (AURKA/Bi) Alisertib, or the PLK1 inhibitor onvansertib (PLK1i) for 24 hours. Organoid lines were treated with vehicle (DMSO) or 0.1µM AURKBi for 24 hours. Prior to harvest, the cell lines were pulsed with BrdU and the organoids were pulsed with EdU. Cells were then harvested, fixed, and stained with appropriate antibodies or chemicals as well as propidium iodide (PI) and then analyzed by flow cytometry. In the analyses shown here, the combined BrdU/EdU and PI data were analyzed together to take S phase into account. The gating strategy from one replicate for ARK1 cells is shown in Panel **A** in the left column for all four mitosis targeting drugs compared to the DMSO control as a representative cell line. The gating strategy from one replicate for EMCA-A or UPSC-A organoids with DMSO and AURKBi is shown in the right column in panels **B** and **C** respectively.

In this type of analysis, the different phases of the cell cycle demarcated by DNA content and BrdU/EdU incorporation are generally represented as a horseshoe which is shown in the far-right plots in each panel, with 1) 2N and 4N cells not duplicating their DNA and not incorporating BrdU or EdU as the bottom arms of the horseshoe, and 2) 2N cells duplicating their DNA to become 4N, and thus incorporating BrdU or EdU, representing S phase and forming the upside-down U top of the horseshoe. The S phase represented by 2N cells duplicating their DNA to become 4N DNA content is what is traditionally analyzed to demarcate G1 (2N DNA content, BrdU/EdU negative), S (BrdU/EdU positive), and G2/M (4N DNA content, BrdU/EdU negative), and that is what was analyzed here as is shown in the gated singlets on the PI-Height (PI-H)/PI-Area (PI-A) plot. However, some 4N cells may be in G1 phase in the setting of drug treatments like an AURKBi and attempt to duplicate their DNA to become >4N, and thus also incorporate BrdU or EdU, which may represent additional difficult to classify S phase cells not shown here. These 4N BrdU/EdU negative cells possibly in G1 phase are given a special name in this Figure as explained below. The potential additional >4N S phase cells, if present, were not analyzed here 1) as they are difficult to classify, 2) as they were not induced by all mitosis targeting drugs and were only present in some cell lines and not present in the slower cycling organoid lines as can be seen in the PI-H/PI-A plot, and 3) as it is only possible to include one S phase population in this type of analysis.

For the combined PI-BrdU or PI-EdU analysis specifically in these mitosis targeting drug experiments, in each case cells were gated on the side scatter (SSC)/forward scatter (FSC) plot. From those cells, single cells (singlets) representing 2N and 4N DNA content cells were gated on the PI-H/PI-A plots to capture the shared BrdU/EdU positive S phase cells. Singlets were then graphed with PI on the X axis and either BrdU-FITC for cell lines or EdU for organoids on the Y axis. Cells which had a 2N DNA content and were negative for BrdU or EdU were gated as G1 phase; cells which had a 4N DNA content and were negative for BrdU or EdU were gated as "*G2M" or "*G2-M" phase on the plots in the figure and are referred to as described below; and cells which were EdU or BrdU positive were gated and quantified as being in S phase as shown in panels A, B, and C. For this Figure and the related Figure S11 only, because AURKBis and some other mitosis targeting drugs can potentially induce 4N G1 cells making 4N DNA content cells which are negative for BrdU or EdU difficult to classify, we are referring to the cells gated as "*G2M" or "*G2-M" in panels A, B, and C and their related quantification in Figures S11B, S11D, and S11E as 4N BrdU/EdU negative cells. For other drug treatments in this work, the 4N BrdU/EdU negative population is referred to as G2/M; and that is carefully explained in the Figure legends and related text.
