# Supplementary material for: Functional Profiling of p53 and RB Cell Cycle Regulatory Proficiency Suggests Mechanism-Driven Molecular Stratification in Endometrial Carcinoma
Source: Cancer Res Commun. 2025 Apr 30;5(4):719–42. doi: 10.1158/2767-9764.CRC-24-0028 (PMC12042793; doi:10.1158/2767-9764.CRC-24-0028)
Supplement: Figure S10 — Supplementary Figure S10 [file crc-24-0028_figure_s10_suppsf10.pdf]

Figure S10

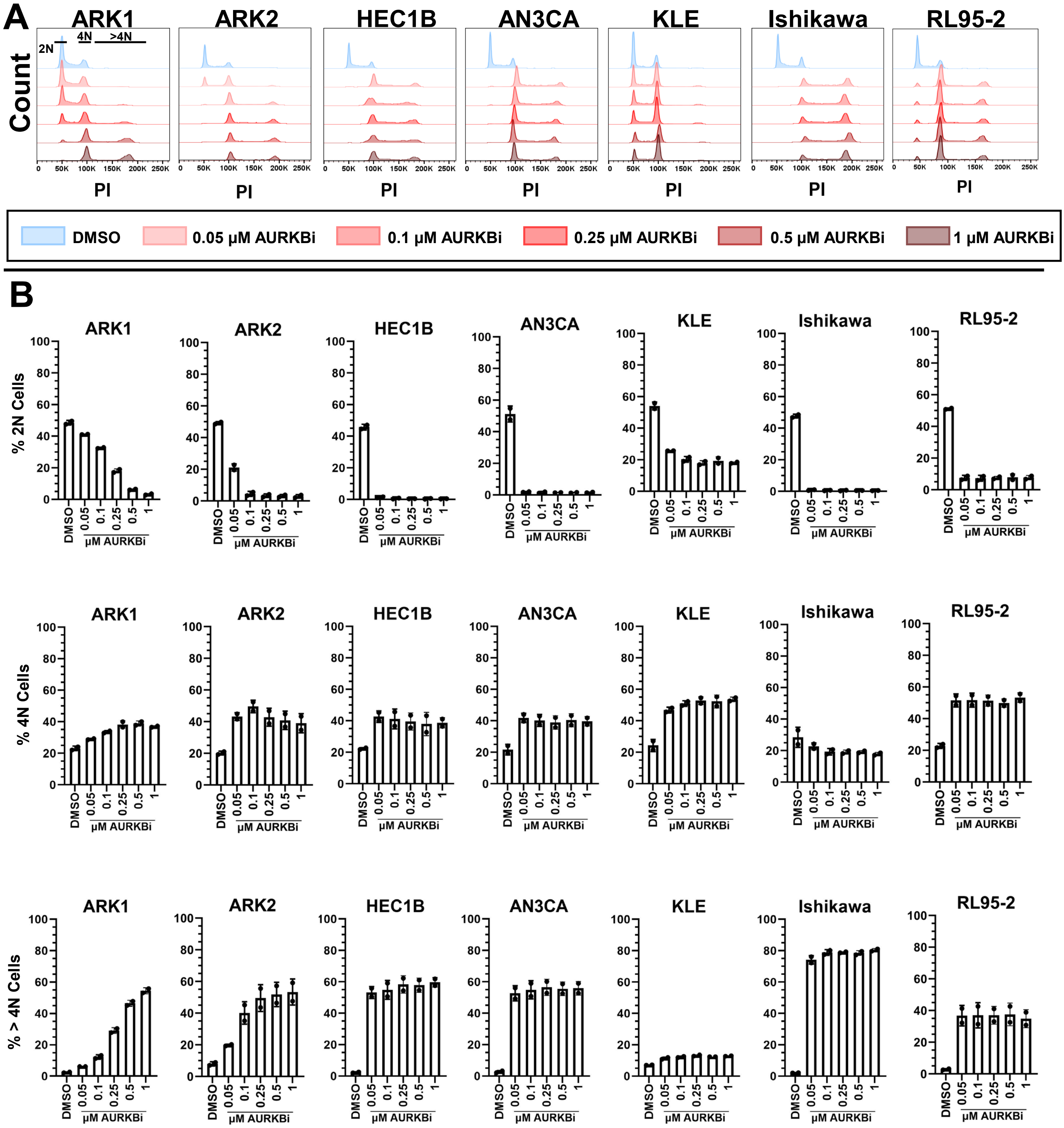

Figure S10. Endometrial cancer cell lines have varying cell cycle dynamics in response to Aurora kinase B inhibition. A and B) Cell lines were treated with vehicle (DMSO) or a dose curve of the Aurora kinase B inhibitor (AURKBi) Barasertib for 24 hours. The cells then underwent bromodeoxyuridine (BrdU)/propidium iodide (PI) cell cycle flow cytometry profiling. Shown in A are the PI profile plots for each cell line from one representative experiment. Shown in B are the percentage of cells with 2N (top), 4N (middle), or greater than 4N (bottom) DNA content for each cell line. Bar graphs represent the percent of cells with the different DNA contents from two independent replicates, and error bars represent standard deviation. See Figure S8 for a representative gating strategy.
