# Supplementary material for: Functional Profiling of p53 and RB Cell Cycle Regulatory Proficiency Suggests Mechanism-Driven Molecular Stratification in Endometrial Carcinoma
Source: Cancer Res Commun. 2025 Apr 30;5(4):719–42. doi: 10.1158/2767-9764.CRC-24-0028 (PMC12042793; doi:10.1158/2767-9764.CRC-24-0028)
Supplement: Figure S11 — Supplementary Figure S11 [file crc-24-0028_figure_s11_suppsf11.pdf]

Figure S11

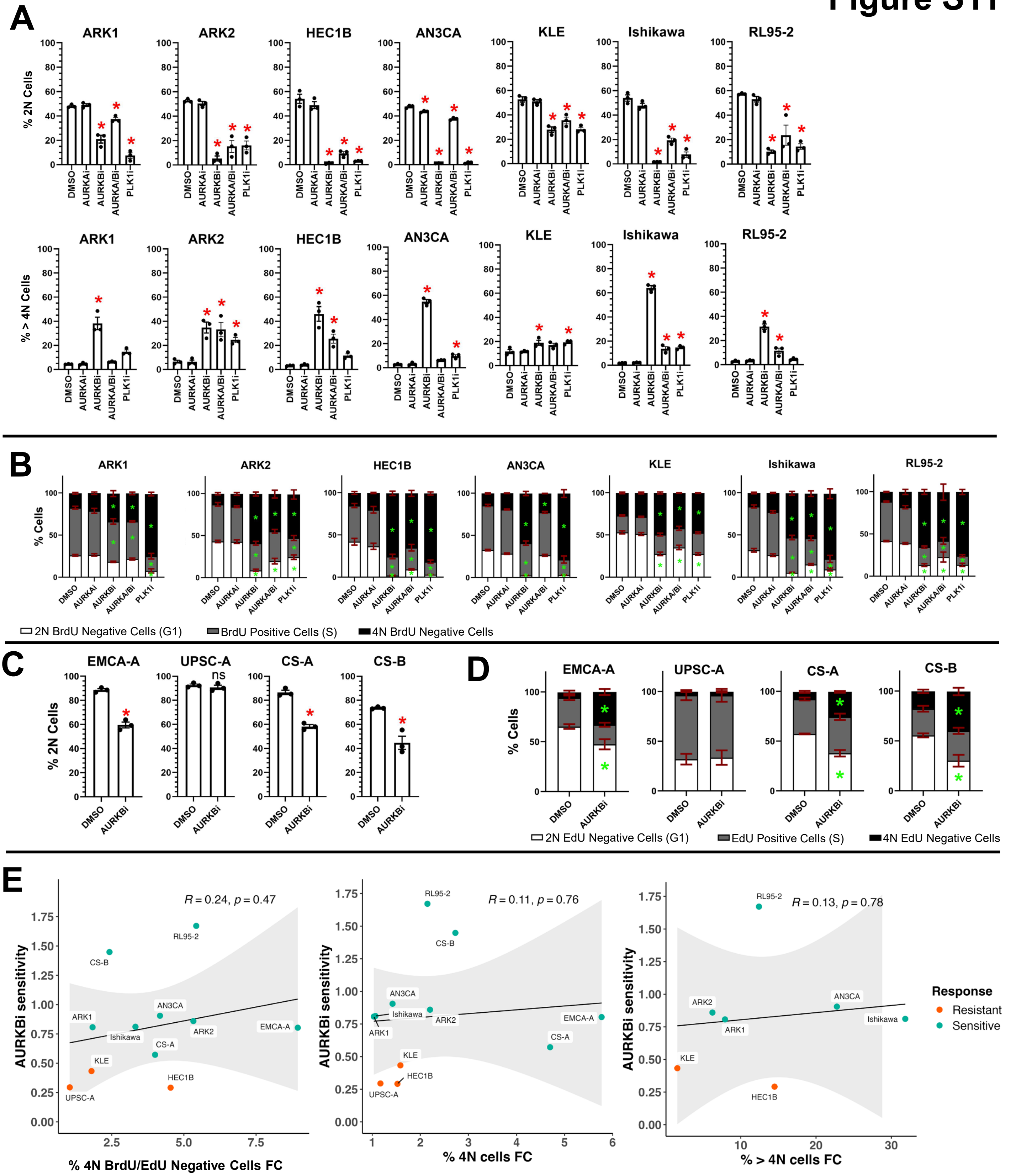

A
ARK1
ARK2
HEC1B
AN3CA
KLE
Ishikawa
RL95-2
% 2N Cells
% > 4N Cells
DMSO
AURKAi
AURKBi
AURKA/Bi
PLK1i
B
% Cells
2N BrdU Negative Cells (G1)
BrdU Positive Cells (S)
4N BrdU Negative Cells
C
EMCA-A
UPSC-A
CS-A
CS-B
ns
% 2N Cells
D
% Cells
2N EdU Negative Cells (G1)
EdU Positive Cells (S)
4N EdU Negative Cells
E
R = 0.24, p = 0.47
R = 0.11, p = 0.76
R = 0.13, p = 0.78
AURKBi sensitivity
% 4N BrdU/EdU Negative Cells FC
% 4N cells FC
% > 4N cells FC
Response
Resistant
Sensitive

Figure S11. Endometrial cancer cell lines have varying cell cycle dynamics in response to low dose mitotic kinase inhibition. A and B) These data represent the remaining quantitation and analysis of the same data shown in the profile plots and bar graphs in Figure 3E. Cell lines were treated with vehicle (DMSO) or 0.1μM of the Aurora kinase A inhibitor MK5108 (AURKAi), the Aurora kinase B inhibitor Barasertib (AURKBi), the dual Aurora kinase A/B inhibitor (AURKA/Bi) Alisertib, or the PLK1 inhibitor onvansertib (PLK1i). The cells then underwent bromodeoxyuridine(BrdU)/propidium iodide (PI) cell cycle flow cytometry profiling. Shown in **A** are the 2N and greater than 4N (>4N) DNA content bar graphs corresponding to data analyzed and shown in Figure 3E where the PI data alone from the flow cytometry was plotted in profile plots and the average percentage of cells with 2N, 4N or >4N DNA content were calculated from the replicates. The bar graphs for percentage of cells with 4N DNA content are shown in Figure 3E, and the bar graphs with the corresponding percentages of cells with 2N or >4N DNA content are shown here. Bars represent the average of three replicates, and error bars represent standard error of the mean. *=p<0.05 compared to DMSO for the specific DNA content by an ordinary one-way ANOVA with Šídák's multiple comparisons test. If there is no *, then the comparison was not significant. Shown in **B** are bar graphs of the combined BrdU/PI analysis of the data from Figure 3E. In these graphs, the bars represent the percentage of cells in each different cell cycle phase as calculated from the three independent replicates, with error bars representing standard error of the mean. G1 represents 2N DNA content BrdU negative cells. 4N DNA content BrdU negative cells are difficult to classify for cell cycle phase with these treatments. S phase represents BrdU positive cells. *=p<0.05 compared to DMSO for the specific cell cycle phase by an ordinary two-way ANOVA with Dunnett's multiple comparisons test. If there is no *, then the comparison was not significant. The color code for the cell cycle phase is below one of the graphs. **C and D)** These data represent the remaining quantitation and analysis of the same data shown in the profile plots and bar graphs in Figure 3F. Organoids were treated with vehicle (DMSO) or 0.1μM AURKBi. Prior to harvest, organoids were pulsed with 5-Ethynyl-2'-deoxyuridine (EdU). Organoids were harvested, fixed, and stained with appropriate chemicals and PI. Shown in **C** are the 2N bar graphs corresponding to data in Figure 3F where the PI data from the flow cytometry was plotted in profile plots and the percentage of cells with 2N or 4N DNA content were calculated. Bars represent the average of three replicates, and error bars represent standard error of the mean. *=p<0.05 compared to DMSO for the specific DNA content by a paired t-test. ns=not significant. Shown in **D** are bar graphs of the combined EdU/PI analysis of the data from Figure 3F. In the graphs, the bars represent the percentage of cells in each different cell cycle phase from the three independent replicates with error bars representing standard error of the mean. G1 represents 2N DNA content EdU negative cells. S phase represents EdU positive cells. 4N DNA content EdU negative cells are difficult to classify for cell cycle phase with this treatment. *=p<0.05 compared to DMSO for the specific cell cycle phase by an ordinary two-way ANOVA with Šídák's multiple comparisons test. If there is no *, then the comparison was not significant. The color code for the cell cycle phase is below one of the graphs. **E)** The scatter plots show the correlation between the median fold change (FC) in the percentage of 4N DNA content BrdU/EdU negative cells (left), the percentage of 4N DNA content cells (middle), or the percentage of >4N DNA content cells (right) following treatment with 0.1μM AURKBi compared to vehicle (DMSO) and the median sensitivity to AURKBi, quantified by the area over the growth rate corrected dose curves. The '*R*' value in the Figure represents the correlation coefficient, indicating the strength and direction of the relationship, while '*p*' represents the p-value obtained from Pearson's correlation analysis. The grey shaded region in the graph represents the 95% confidence interval for the black linear regression line in the graph. The lines were determined to be sensitive if the AURKBi sensitivity was greater than 0.5 as shown in Figure 3D, and this data was used for sensitivity values. For the plots in the middle and on the right, the percentage of cells with 4N or >4N DNA content post AURKBi compared to vehicle were calculated from the profile plots and related bar graphs shown in Main text Figures 3E and 3F for 4N DNA content for cell lines and organoid lines and in panel S11A above for >4N DNA content for cell lines only. For the plots on the left, the percentage of 4N DNA content BrdU/EdU negative cells was calculated from the data in the bar graphs in panels S11B and S11D above.
