# Supplementary material for: Functional Profiling of p53 and RB Cell Cycle Regulatory Proficiency Suggests Mechanism-Driven Molecular Stratification in Endometrial Carcinoma
Source: Cancer Res Commun. 2025 Apr 30;5(4):719–42. doi: 10.1158/2767-9764.CRC-24-0028 (PMC12042793; doi:10.1158/2767-9764.CRC-24-0028)
Supplement: Figure S12 — Supplementary Figure S12 [file crc-24-0028_figure_s12_suppsf12.pdf]

Figure S12

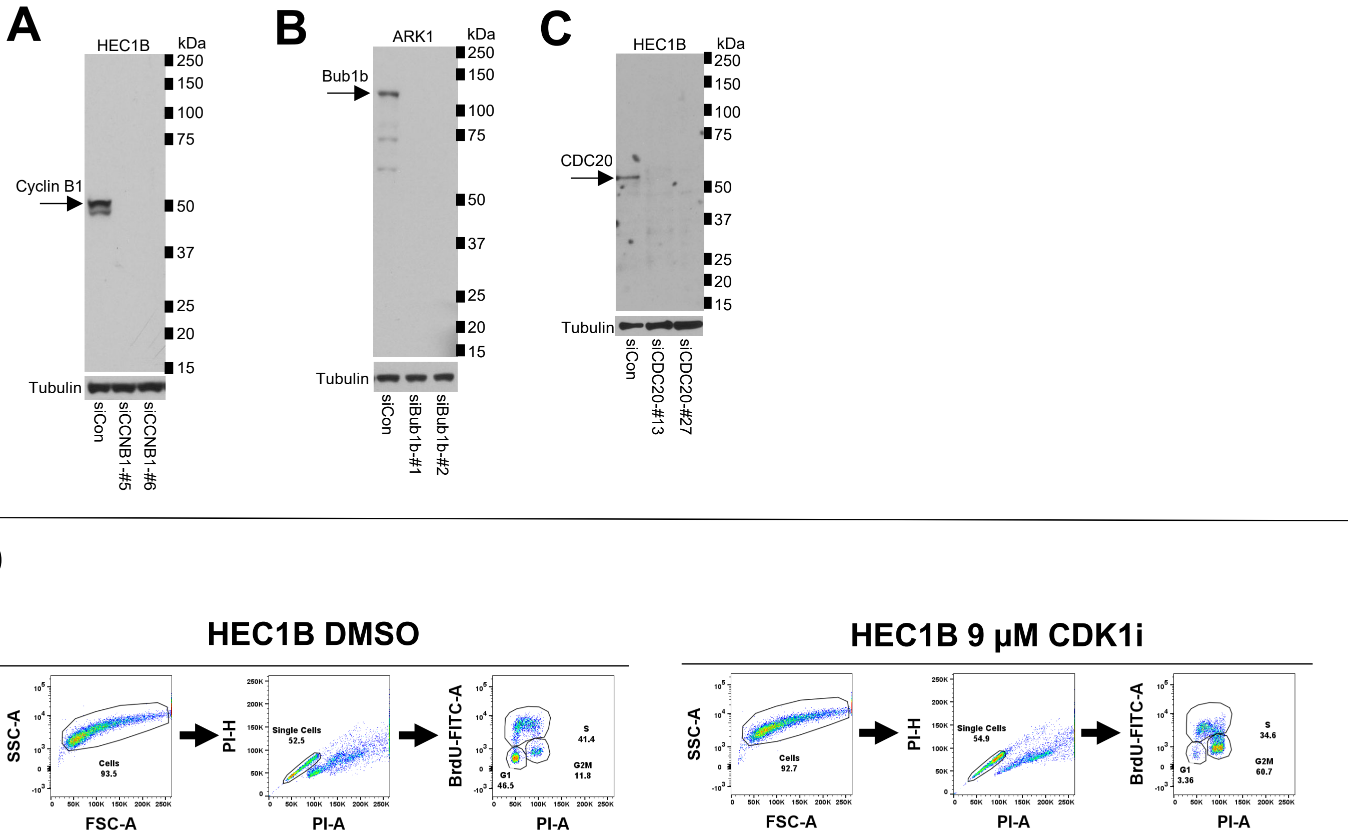

Figure S12. Western blot validation for mitotic protein antibodies and representative gating strategy for optimizing G2/M synchronization of endometrial cancer cell lines with a CDK1 inhibitor. A) HEC1B cells were transfected with siCon or two different Cyclin B1 siRNAs (siCCNB1-#5 and siCCNB1-#6). Lysates were prepared and a western blot performed. The membrane was first stained for Cyclin B1 and then stripped and re-probed for tubulin. **B)** ARK1 cells were transfected with a control siRNA (siCon) or two different Bub1b siRNAs (siBub1b-#1 and siBub1b-#2). Lysates were prepared and a western blot performed. The membrane was first stained for Bub1b and then stripped and re-probed for tubulin. **C)** HEC1B cells were transfected with siCon or two different CDC20 siRNAs (siCDC20-#13 or siCDC20-#27). Lysates were prepared and a western blot performed. The membrane was first stained for CDC20 and then stripped and re-probed for tubulin. **D)** This data corresponds to the data shown in Figure 4B. HEC1B cells were treated with vehicle (DMSO) or a dose curve of the CDK1 inhibitor (CDK1i) Ro-3306 for 16 hours. One hour prior to harvest, the cells were pulsed with bromodeoxyuridine (BrdU). Cells were harvested, fixed, and stained for BrdU/propidium iodide (PI) and then analyzed on a flow cytometer. Shown here is a general gating strategy for one replicate of the HEC1B cell line treated with DMSO (left) or 9µM CDK1i (right). In each case, cells were first gated on the side scatter (SSC)/forward scatter (FSC) plot. From cells, singlets representing cells with 2N and 4N DNA content were gated on the PI-Height (PI-H)/PI-Area (PI-A) plot. Singlets were then graphed with PI on the X axis and BrdU-FITC on the Y axis. Cells which had a 2N DNA content and were negative for BrdU were gated as G1 phase. Cells which had a 4N DNA content and were negative for BrdU were gated as G2/M phase cells. Cells which were BrdU positive were gated and quantified as being in S phase. These plots are from one representative DMSO and 9µM CDK1i replicate from the HEC1B data shown in Figure 4B.
