# Supplementary material for: Functional Profiling of p53 and RB Cell Cycle Regulatory Proficiency Suggests Mechanism-Driven Molecular Stratification in Endometrial Carcinoma
Source: Cancer Res Commun. 2025 Apr 30;5(4):719–42. doi: 10.1158/2767-9764.CRC-24-0028 (PMC12042793; doi:10.1158/2767-9764.CRC-24-0028)
Supplement: Figure S13 — Supplementary Figure S13 [file crc-24-0028_figure_s13_suppsf13.pdf]

Figure S13

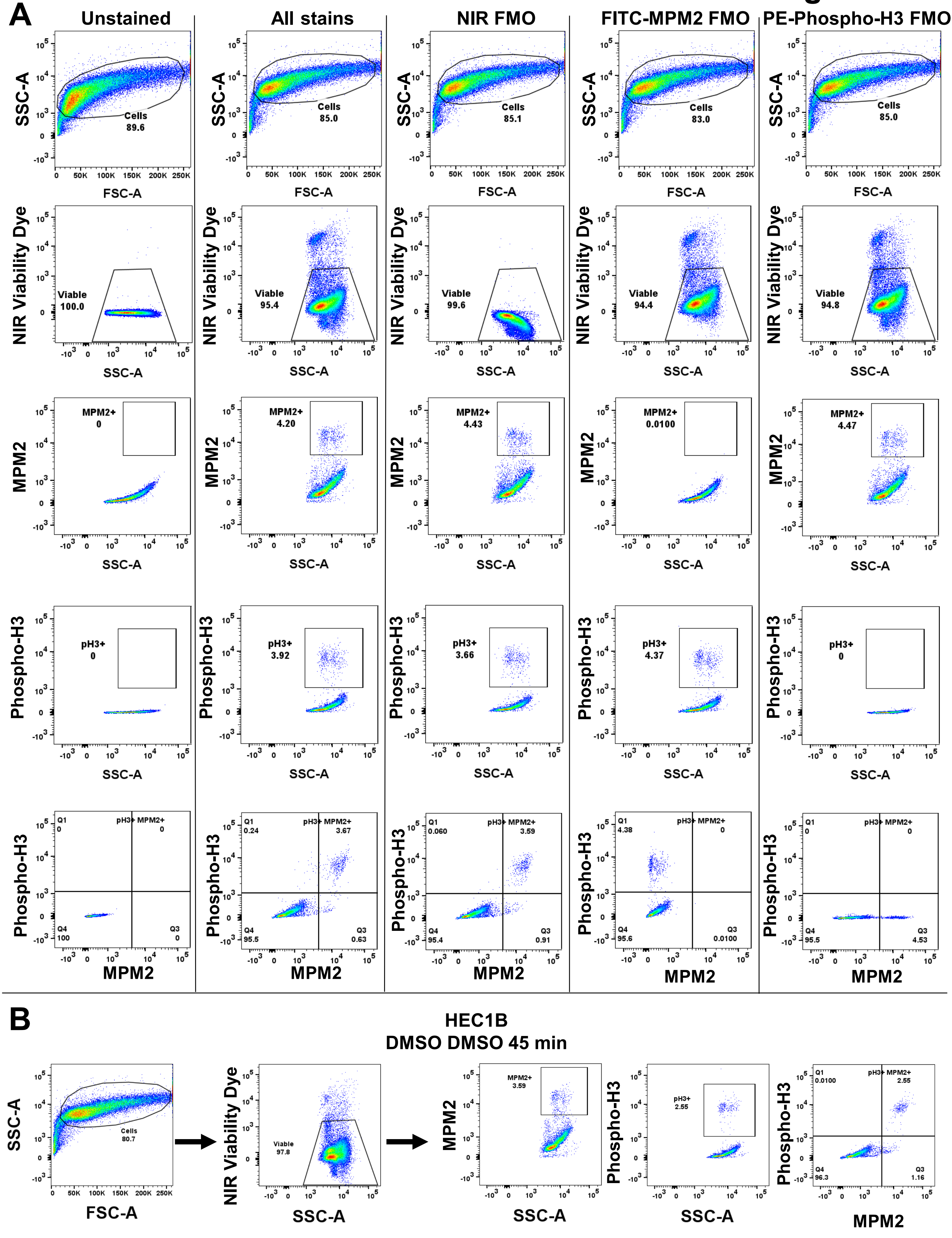
A
Unstained
All stains
NIR FMO
FITC-MPM2 FMO
PE-Phospho-H3 FMO
SSC-A
FSC-A
Cells
89.6
85.0
85.1
83.0
85.0
NIR Viability Dye
Viable
100.0
95.4
99.6
94.4
94.8
MPM2
MPM2+
0
4.20
4.43
0.0100
4.47
Phospho-H3
pH3+
0
3.92
3.66
4.37
0
Q1
pH3+ MPM2+
Q3
Q4
100
0.24
3.67
95.5
0.63
0.060
3.59
95.4
0.91
4.38
95.6
0.0100
4.53
B
HEC1B
DMSO DMSO 45 min
80.7
97.8
3.59
2.55
0.0100
96.3
1.16

Figure S13. Flow cytometry mitotic antibody validation and gating strategy. A) NIR viability dye, FITC-conjugated secondary antibody stained MPM2 antibody, and PE-conjugated histone H3 phosphorylated on serine 10 (Phospho-H3 or pH3) antibody together were validated as not strongly interfering with fluorescence readings from other channels; and gates were set based on this validation. Untreated HEC1B cells were stained as follows for this validation study. HEC1B cells 1) remained unstained, 2) were stained for NIR viability dye, MPM2 and FITC-conjugated secondary antibody (FITC-MPM2), and PE-Phospho-H3 (All stains), 3) were stained only for FITC-MPM2 and PE-Phospho-H3 (NIR Fluorescence Minus One (FMO)), 4) were stained only for NIR viability dye and PE-Phospho-H3 (FITC-MPM2 FMO), or 5) were stained only for NIR viability dye and FITC-MPM2 (PE-Phospho-H3 FMO). The results of this staining are shown in columns with gating from top to bottom in each column and with the staining for the column indicated at the top of the column in Panel A. In each case, cells were first gated on the side scatter (SSC)/forward scatter (FSC) plot as shown on the top in each column. From the "Cells" population, viable cells were gated on the NIR Viability Dye/SSC plot with the NIR FMO used to help set the gate. From viable cells, MPM2 positive (MPM2+), Phospho-H3 positive (pH3+), or Phospho-H3/MPM2 double positive (pH3+ MPM2+) cells were gated based on the FMO staining for the different antibodies. **B)** HEC1B cells underwent various treatments and then were stained for NIR Viability Dye, MPM2, and Phospho-H3 exactly as detailed in Main Text Materials and Methods. The general flow cytometry gating strategy for NIR Viability Dye, MPM2, Phospho-H3 stained cells is shown here for one representative replicate of a DMSO DMSO control sample at 45 minutes (min) post-release. Cells were gated on the side scatter (SSC)/forward scatter (FSC) plot. From the "Cells" population, cells negative for NIR viability dye were gated as viable cells on the NIR viability dye/SSC plot. From those viable cells, total MPM2 positive (MPM2+), total Phospho-H3 positive (pH3+), or double Phospho-H3/MPM2 positive (pH3+ MPM2+) cells were gated as shown. This strategy was used for all cell lines for this staining method in Figures 4C, 4D, 5C, 5D, S14, S18, S20, S21A, S21B, and S21C.
