# Supplementary material for: Functional Profiling of p53 and RB Cell Cycle Regulatory Proficiency Suggests Mechanism-Driven Molecular Stratification in Endometrial Carcinoma
Source: Cancer Res Commun. 2025 Apr 30;5(4):719–42. doi: 10.1158/2767-9764.CRC-24-0028 (PMC12042793; doi:10.1158/2767-9764.CRC-24-0028)
Supplement: Figure S15 — Supplementary Figure S15 [file crc-24-0028_figure_s15_suppsf15.pdf]

Figure S15

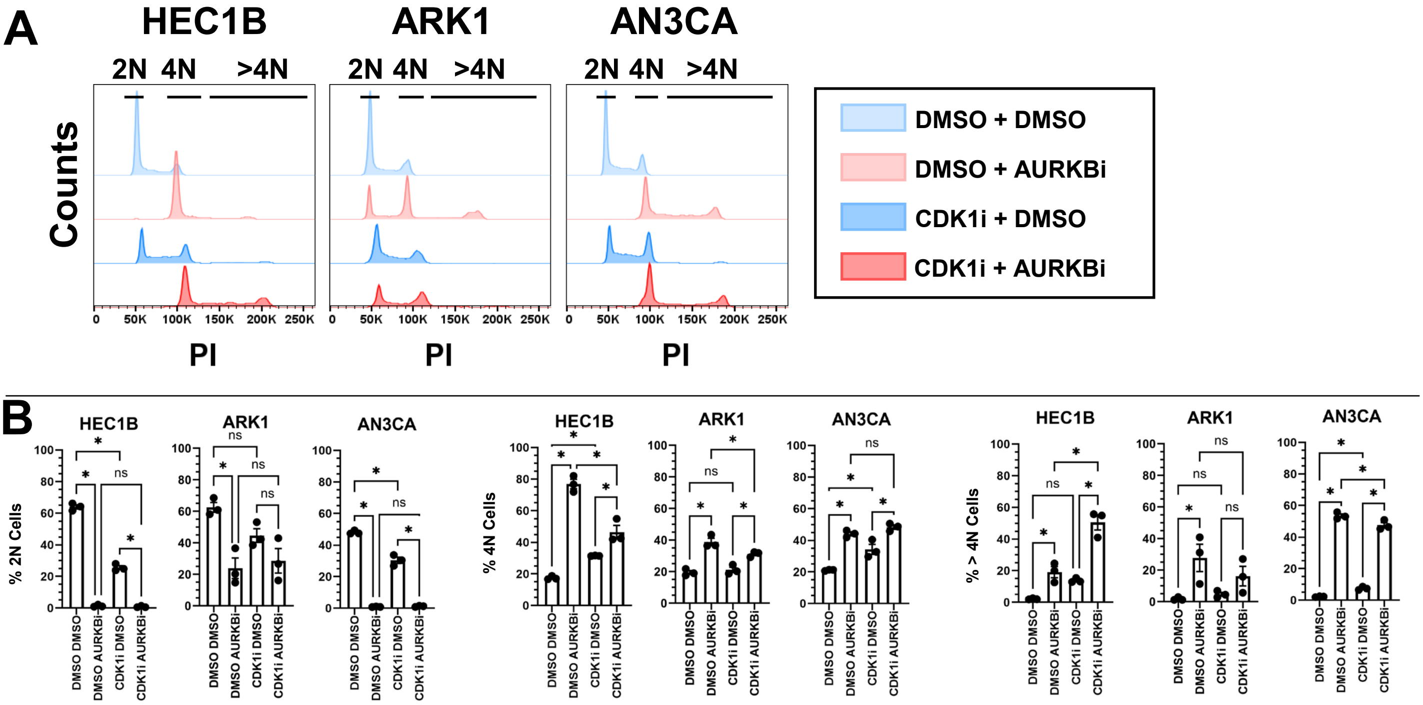

Figure S15. Cell cycle profiling data corresponding to Figures 4C and 4D. A and B) HEC1B, ARK1, or AN3CA cells were treated with vehicle (DMSO) or the CDK1 inhibitor (CDK1i) Ro-3306 for 16 hours, washed, and then treated with media containing either vehicle (DMSO) or the Aurora kinase B inhibitor (AURKBi) Barasertib for 24 hours. One hour prior to harvest, cells were pulsed with bromodeoxyuridine (BrdU). Cells were then harvested, stained for BrdU and Propidium Iodide (PI), and analyzed on a flow cytometer. The experiment was repeated three times. Representative PI profile plots are shown in Panel **A** from one experiment for each cell line with each drug treatment. The color code to the treatments is on the right. Bars/labels denoting peaks for 2N, 4N, and greater than 4N (>4N) DNA content are shown on the top of each stack. In panel **B**, the PI data from the three experiments was analyzed alone for DNA content, and the percentage of cells with 2N, 4N, or >4N DNA content are shown in bar graphs. Bars represent the average from the three experiments while error bars represent standard error of the mean. *=p<0.05 and ns=not significant compared to the treatment indicated by the bracket over the bars by an ordinary one-way ANOVA with Šídák's multiple comparisons test. Please see the representative gating strategy for this type of flow cytometry in Figure S8.
