# Supplementary material for: Functional Profiling of p53 and RB Cell Cycle Regulatory Proficiency Suggests Mechanism-Driven Molecular Stratification in Endometrial Carcinoma
Source: Cancer Res Commun. 2025 Apr 30;5(4):719–42. doi: 10.1158/2767-9764.CRC-24-0028 (PMC12042793; doi:10.1158/2767-9764.CRC-24-0028)
Supplement: Figure S16 — Supplementary Figure S16 [file crc-24-0028_figure_s16_suppsf16.pdf]

Figure S16

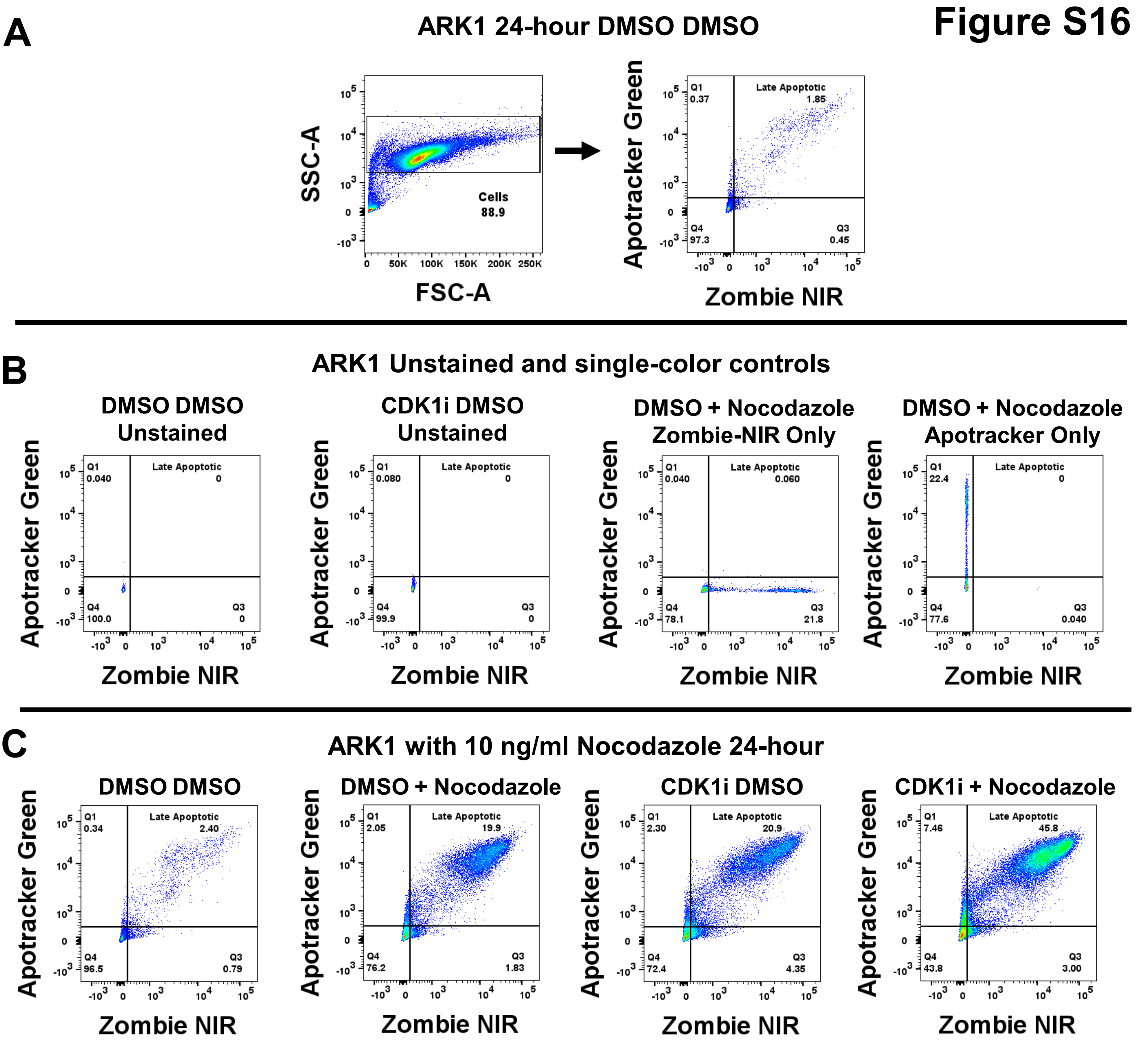

Figure S16. Gating strategy for Zombie NIR Viability Dye/Apotracker Green double stain flow cytometry. ARK1, HEC1B, or AN3CA cells were treated with vehicle (DMSO) or the CDK1 inhibitor (CDK1i) Ro-3306 for 16 hours, washed, and then treated with media containing either vehicle (DMSO) or Aurora kinase B inhibitor or vehicle (DMSO) or 10ng/mL or 20ng/mL (ARK1 and HEC1B only) nocodazole for 24 hours. Cells were harvested and either remained unstained, or were stained for Zombie NIR Viability Dye alone, Apotracker Green alone, or Zombie NIR Viability Dye and Apotracker Green together. Cells were then analyzed by flow cytometry. A representative gating strategy is shown here from some of the replicates of the ARK1 data shown in Figure 5E. **A)** A representative vehicle control ARK1 cell dataset is shown here to demonstrate the overall gating strategy. Cells were acquired on a side scatter (SSC)/forward scatter (FSC) plot, and then the cells to be analyzed were gated. Those cells were then plotted with Apotracker Green on the Y axis and Zombie NIR on the X-axis, and a quadrant gate was applied. **B and C)** Shown in B and C are the unstained and single-color controls (B) and a matched treated all stain data set (C) to demonstrate how the quadrant gate was set for each experiment. **B)** The quadrant gate applied in each experiment was set for the experiment based on the unstained cells shown in the two left panels here and the single stains for Zombie NIR in the third panel and for Apotracker in the fourth panel for the experiment. **C)** Shown here are quadrant gates for double stained ARK1 cells across four treatments using the quadrant gate based on the controls in B. The cells were gated and gates applied as shown in A and B. Calculations for the percentage of Apotracker/viability dye double positive cells, referred to as “late apoptotic cells,” were made based on these quadrant gates.
