# Supplementary material for: Functional Profiling of p53 and RB Cell Cycle Regulatory Proficiency Suggests Mechanism-Driven Molecular Stratification in Endometrial Carcinoma
Source: Cancer Res Commun. 2025 Apr 30;5(4):719–42. doi: 10.1158/2767-9764.CRC-24-0028 (PMC12042793; doi:10.1158/2767-9764.CRC-24-0028)
Supplement: Figure S17 — Supplementary Figure S17 [file crc-24-0028_figure_s17_suppsf17.pdf]

Figure S17

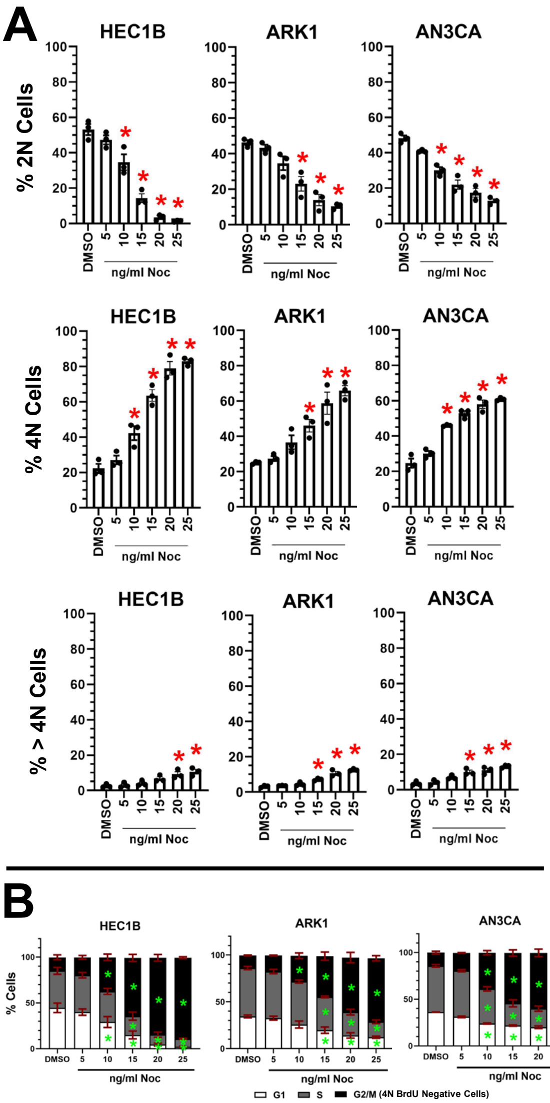

Figure S17. Endometrial cancer cell lines have varying responses to nocodazole-induced interference in mitotic spindle dynamics. A and B) Shown here is additional analysis of all replicates of the bromodeoxyuridine (BrdU)/propidium iodide (PI) cell cycle flow cytometry profiling data shown in Main Text Figure 5B.

HEC1B, ARK1, and AN3CA cells were treated with vehicle (DMSO) or a dose curve of nocodazole (Noc) for 24 hours. The cells then underwent BrdU/PI cell cycle flow cytometry profiling, and the data is shown in two ways, in Main Text Figure 5B and Panel A here as PI profile plots with the percentage of cells with a specific DNA content quantified from the PI data alone, and in Panel B here as stacked bar graphs showing the percent of cells in each cell cycle phase having analyzed the BrdU and PI data together. The experiment was repeated three times.

Shown in **A** are the percentages of cells with 2N, 4N, or greater than 4N (>4N) DNA content corresponding to the PI profile plots in Main Text Figure 5B, where PI profile plots from one representative experiment were shown for each cell line. The percentages of cells with 2N, 4N, or >4N DNA content were calculated from the PI data alone from the three independent experiments and are shown in bar graphs here. Bars represent the average of three replicates, and error bars represent standard error of the mean. *=$p<0.05$ compared to DMSO for the specific DNA content for the specific cell line by an ordinary one-way ANOVA with Šídák's multiple comparisons test. If there is no *, then the comparison was not significant.

Shown in **B** is the combined BrdU/PI data corresponding to the data from Main Text Figure 5B and also Panel A above. Shown are bar graphs with bars representing the average percent of cells in each different cell cycle phase from combined BrdU/PI analysis of the same three independent replicates analyzed in Panel A above. Error bars represent standard error of the mean. G1 represents 2N DNA content cells that are BrdU negative, S represents BrdU positive cells, and G2/M represents 4N DNA content cells that are BrdU negative. *=$p<0.05$ compared to DMSO for the specific cell cycle phase by an ordinary two-way ANOVA with Dunnett's multiple comparisons test. If there is no *, then the comparison was not significant. The color code for the cell cycle phase is below one of the graphs.

Please see Figures S8 (corresponds to A) and S4 (corresponds to B) for representative gating strategies for this type of flow cytometry but with different treatments.
