# Supplementary material for: Functional Profiling of p53 and RB Cell Cycle Regulatory Proficiency Suggests Mechanism-Driven Molecular Stratification in Endometrial Carcinoma
Source: Cancer Res Commun. 2025 Apr 30;5(4):719–42. doi: 10.1158/2767-9764.CRC-24-0028 (PMC12042793; doi:10.1158/2767-9764.CRC-24-0028)
Supplement: Figure S18 — Supplementary Figure S18 [file crc-24-0028_figure_s18_suppsf18.pdf]

Figure S18

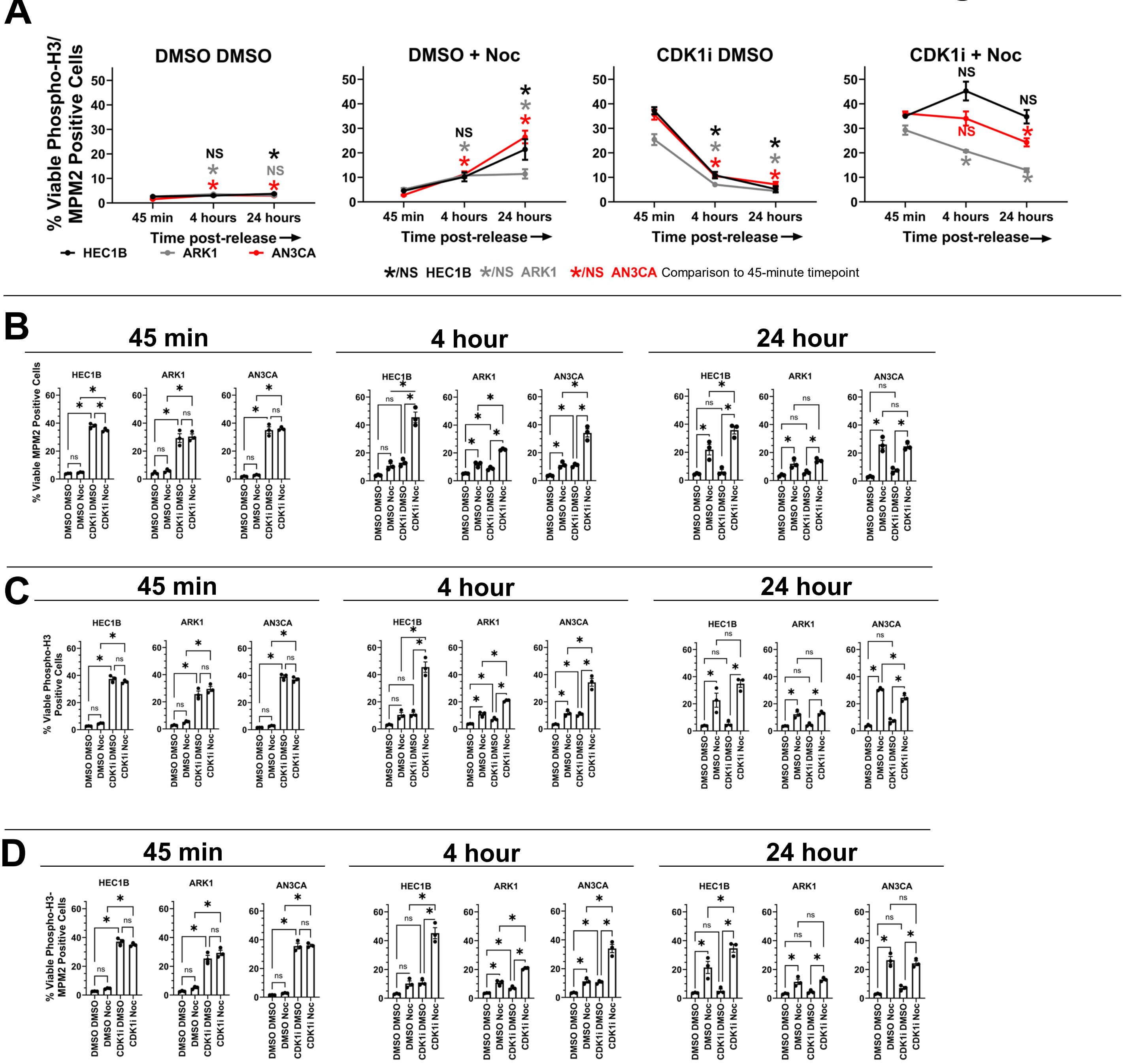

A
DMSO DMSO
DMSO + Noc
CDK1i DMSO
CDK1i + Noc
% Viable Phospho-H3/ MPM2 Positive Cells
45 min
4 hours
24 hours
Time post-release
NS
HEC1B
ARK1
AN3CA
*/NS HEC1B */NS ARK1 */NS AN3CA Comparison to 45-minute timepoint
B
45 min
4 hour
24 hour
% Viable MPM2 Positive Cells
DMSO DMSO
DMSO Noc
CDK1i DMSO
CDK1i Noc
ns
C
% Viable Phospho-H3 Positive Cells
D
% Viable Phospho-H3-MPM2 Positive Cells

Figure S18. Phospho-histone H3/MPM2 double positive data and additional statistical comparisons for CDK1 inhibitor/10ng/mL nocodazole data corresponding to Figure 5. A) The data shown in panel A is an additional analysis of the data shown in Figures 5C and 5D. ARK1, HEC1B, or AN3CA cells were treated with vehicle (DMSO) or the CDK1 inhibitor (CDK1i) Ro-3306 for 16 hours, washed, and then treated with media containing either vehicle (DMSO) or 10ng/mL nocodazole (Noc). Cells were harvested and stained for NIR viability dye, MPM2, and histone H3 phosphorylated on serine 10 (Phospho-H3) at 45 minutes (min), 4 hours, or 24 hours post-release from vehicle or CDK1i. The data was analyzed for Viable MPM2 or Phospho-H3 single positive cells which is shown in Figures 5C and 5D. Shown here are line graphs revealing the average percentage of Viable Phospho-H3/MPM2 double positive cells at each timepoint from the same data sets analyzed in Figures 5C and 5D. The individual points for each cell line in the individual graph for each treatment represent the average of three experiments, and error bars represent standard error of the mean. An ordinary one-way ANOVA with Šídák's multiple comparisons test was performed to assess the significance of the difference between either the 4 hour or the 24 hour timepoint and the 45 minute timepoint for each cell line within each treatment. The color code for the cell lines is shown below one of the graphs on the far left. The color code for the statistical markers is underneath the graphs in the middle. *=$p<0.05$, and NS=not significant compared to the 45 minute timepoint for the individual cell line with the individual drug combination, with the color of the * or letters corresponding to the cell line. **B, C, and D)** The data shown here is the same data analyzed in Figures 5C, 5D, and S18A but presented in a different way to allow for different statistical comparisons to be made. In those experiments, HEC1B, ARK1, and AN3CA cells were treated with vehicle (DMSO) or CDK1i for 16 hours, washed, and then treated with vehicle (DMSO) or 10ng/mL nocodazole (Noc). Cells were harvested at 45 minutes (min), 4 hours, and 24 hours post-release and analyzed for different markers by flow cytometry. In the line graphs in the previous Figures 5C, 5D, and S18A, the average percentage of viable cells positive for each marker at each timepoint was shown for one drug combination with timepoints on the X-axis and percent positive cells on the Y-axis. This allowed only for comparisons between the three timepoints for a single drug combination in a model.

Here to instead allow for statistical comparisons between each of the four drug treatments in one cell line at one timepoint, we are showing the data as bar graphs with the four drug treatments on the X-axis and the percentage of cells positive for the marker on the Y-axis. Panel B shows %Viable MPM2 positive cells and corresponds to Figure 5C, Panel C shows %Viable histone H3 phosphorylated on serine 10 (Phospho-H3) positive cells and corresponds to Figure 5D, and Panel D shows %Viable Phospho-H3/MPM2 double positive cells and corresponds to Figure S18A. There is one bar graph for each cell line at each timepoint. The bars in these graphs represent the average percentage of cells positive for the marker from the three independent replicates analyzed in the previous Figures. Error bars represent standard error of the mean. Brackets demonstrate statistical comparisons between different treatments at each timepoint, and statistical significance was determined by ordinary one-way ANOVA with Šídák's multiple comparisons test. *=$p<0.05$ and ns = not significant. Timepoints are indicated above the bar graphs.
