# Supplementary material for: Functional Profiling of p53 and RB Cell Cycle Regulatory Proficiency Suggests Mechanism-Driven Molecular Stratification in Endometrial Carcinoma
Source: Cancer Res Commun. 2025 Apr 30;5(4):719–42. doi: 10.1158/2767-9764.CRC-24-0028 (PMC12042793; doi:10.1158/2767-9764.CRC-24-0028)
Supplement: Figure S19 — Supplementary Figure S19 [file crc-24-0028_figure_s19_suppsf19.pdf]

Figure S19

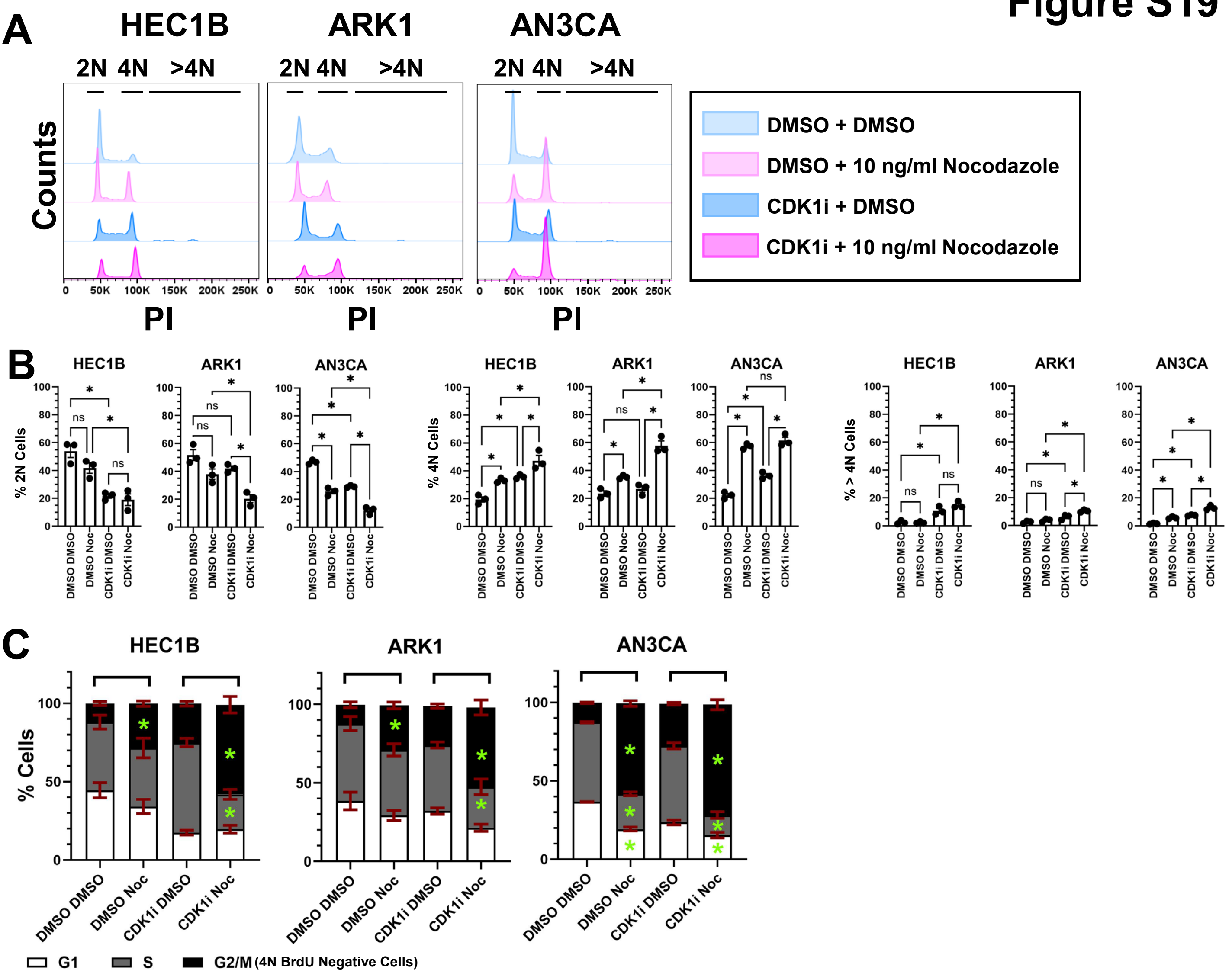

Figure S19. Cell cycle profiling data corresponding to Figures 5 and S18. A, B, and C) HEC1B, ARK1, or AN3CA cells were treated with vehicle (DMSO) or the CDK1 inhibitor (CDK1i) Ro-3306 for 16 hours, washed, and then treated with media containing either vehicle (DMSO) or 10ng/mL nocodazole (Noc) for 24 hours. One hour prior to harvest, cells were pulsed with bromodeoxyuridine (BrdU). Cells were then harvested, stained for BrdU and Propidium Iodide (PI), and analyzed on a flow cytometer. The experiment was repeated three times. Representative PI profile plots are shown in Panel **A** from one experiment for each cell line with each drug treatment. The color code to the treatments is on the right. Bars/labels denoting peaks for 2N, 4N, and greater than 4N (>4N) DNA content are shown on the top of each stack. The data was analyzed in two ways including the PI data alone for DNA content shown in Panel B and the combined PI/BrdU data to include S phase cells shown in Panel C. In panel **B**, the PI data from the three experiments was analyzed alone for DNA content, and the percentage of cells with 2N, 4N, or >4N DNA content from the three separate experiments is represented in the bar graphs. Bars represent the average from the three experiments while error bars represent standard error of the mean. *=p<0.05 or ns=not significant compared to the treatment indicated by the bracket over the bars by an ordinary one-way ANOVA with Šídák's multiple comparisons test. Please see the representative gating strategy in Figure S8 for the gating strategy used here. In Panel **C**, the combined PI/BrdU data analysis is represented. Shown are bar graphs with bars representing the percent of cells in each different cell cycle phase from the three independent replicates and error bars representing standard error of the mean. G1 represents 2N DNA content cells that are BrdU negative, S represents BrdU positive cells, and G2/M represents 4N DNA content cells that are BrdU negative. *=p<0.05 with comparisons indicated by bracket for the specific cell cycle phase by an ordinary two-way ANOVA with Šídák's multiple comparisons test. If there is no *, then the comparison was not significant. The color code for the cell cycle phase is below one of the graphs. Please see the representative gating strategy in Figure S4 for the gating strategy used here.
