# Supplementary material for: Functional Profiling of p53 and RB Cell Cycle Regulatory Proficiency Suggests Mechanism-Driven Molecular Stratification in Endometrial Carcinoma
Source: Cancer Res Commun. 2025 Apr 30;5(4):719–42. doi: 10.1158/2767-9764.CRC-24-0028 (PMC12042793; doi:10.1158/2767-9764.CRC-24-0028)
Supplement: Figure S20 — Supplementary Figure S20 [file crc-24-0028_figure_s20_suppsf20.pdf]

Figure S20

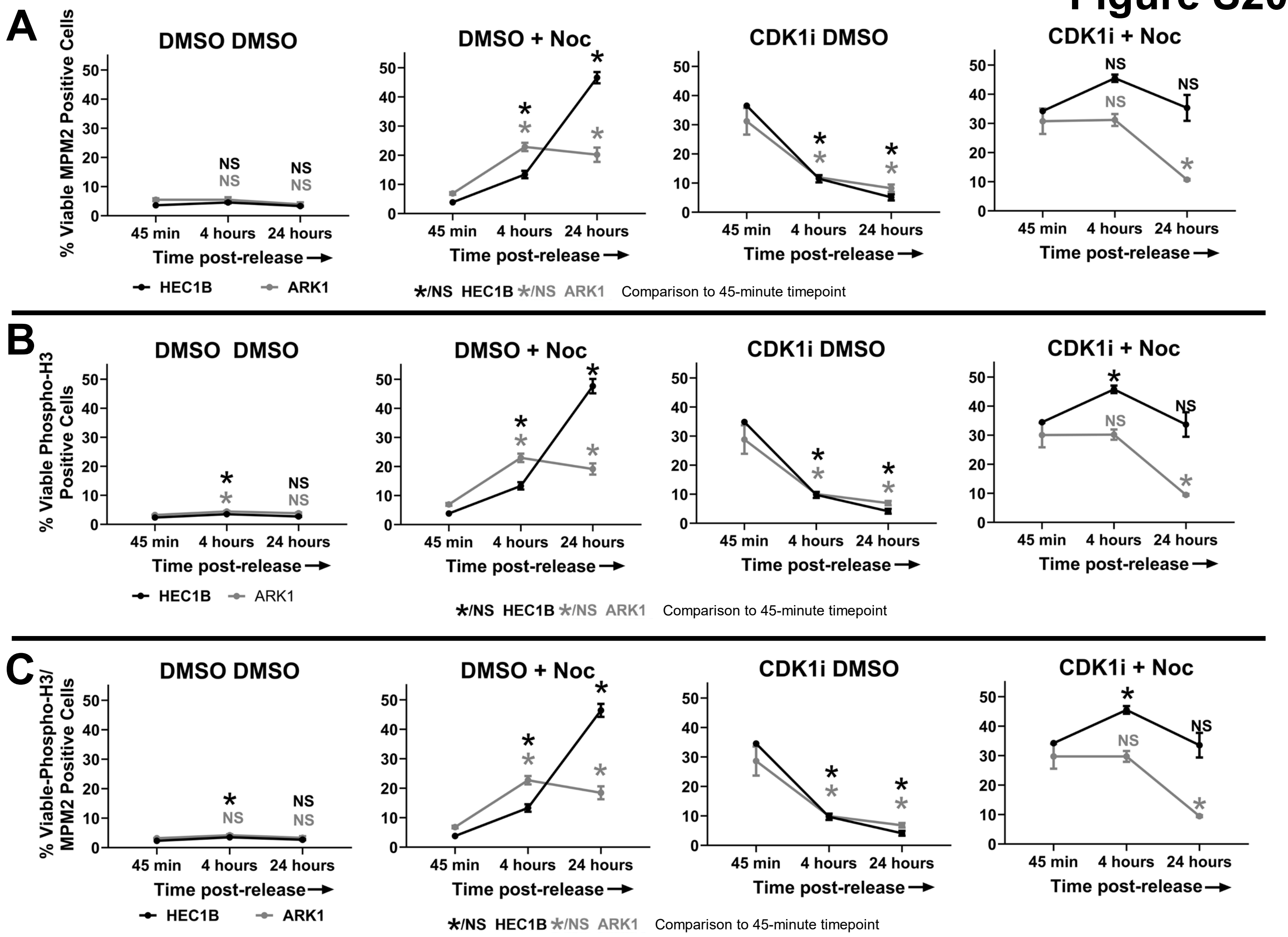

Figure S20. HEC1B and ARK1 cells behave similarly when released from synchronization with a CDK1 inhibitor into 20ng/mL nocodazole as with 10 ng/mL nocodazole. A, B, and C) HEC1B and ARK1 cells were treated with vehicle (DMSO) or the CDK1 inhibitor (CDK1i) Ro-3306 for 16 hours, washed, and then treated with vehicle (DMSO) or 20ng/mL nocodazole (Noc). Cells were harvested at 45 minutes (min), 4 hours, or 24 hours post release from vehicle/CDK1i. Cells were then stained for MPM2, histone H3 phosphorylated on serine 10 (Phospho-H3), and viability dye. Line graphs showing the percentage of cells positive for a marker for each cell line at each timepoint with each treatment are shown for Viable MPM2 positive cells (A), Viable Phospho-H3 positive cells (B), and Viable Phospho-H3/MPM2 double positive cells (C). For A-C, the individual points for each cell line in the individual graph for each treatment represent the average of three experiments, and error bars represent standard error of the mean. An ordinary one-way ANOVA with Šídák's multiple comparisons test was performed to assess the significance of the difference between either the 4 hour or the 24-hour timepoint and the 45-minute timepoint for each cell line within each treatment. The color code for the cell lines is shown below one of the graphs on the far left. The color code for the statistical markers is underneath the graphs in the middle. *=$p<0.05$, and NS=not significant compared to the 45-minute timepoint for the individual cell line with the individual drug combination, with the color of the * or letters corresponding to the cell line.
