# Supplementary material for: Functional Profiling of p53 and RB Cell Cycle Regulatory Proficiency Suggests Mechanism-Driven Molecular Stratification in Endometrial Carcinoma
Source: Cancer Res Commun. 2025 Apr 30;5(4):719–42. doi: 10.1158/2767-9764.CRC-24-0028 (PMC12042793; doi:10.1158/2767-9764.CRC-24-0028)
Supplement: Figure S21 — Supplementary Figure S21 [file crc-24-0028_figure_s21_suppsf21.pdf]

Figure S21

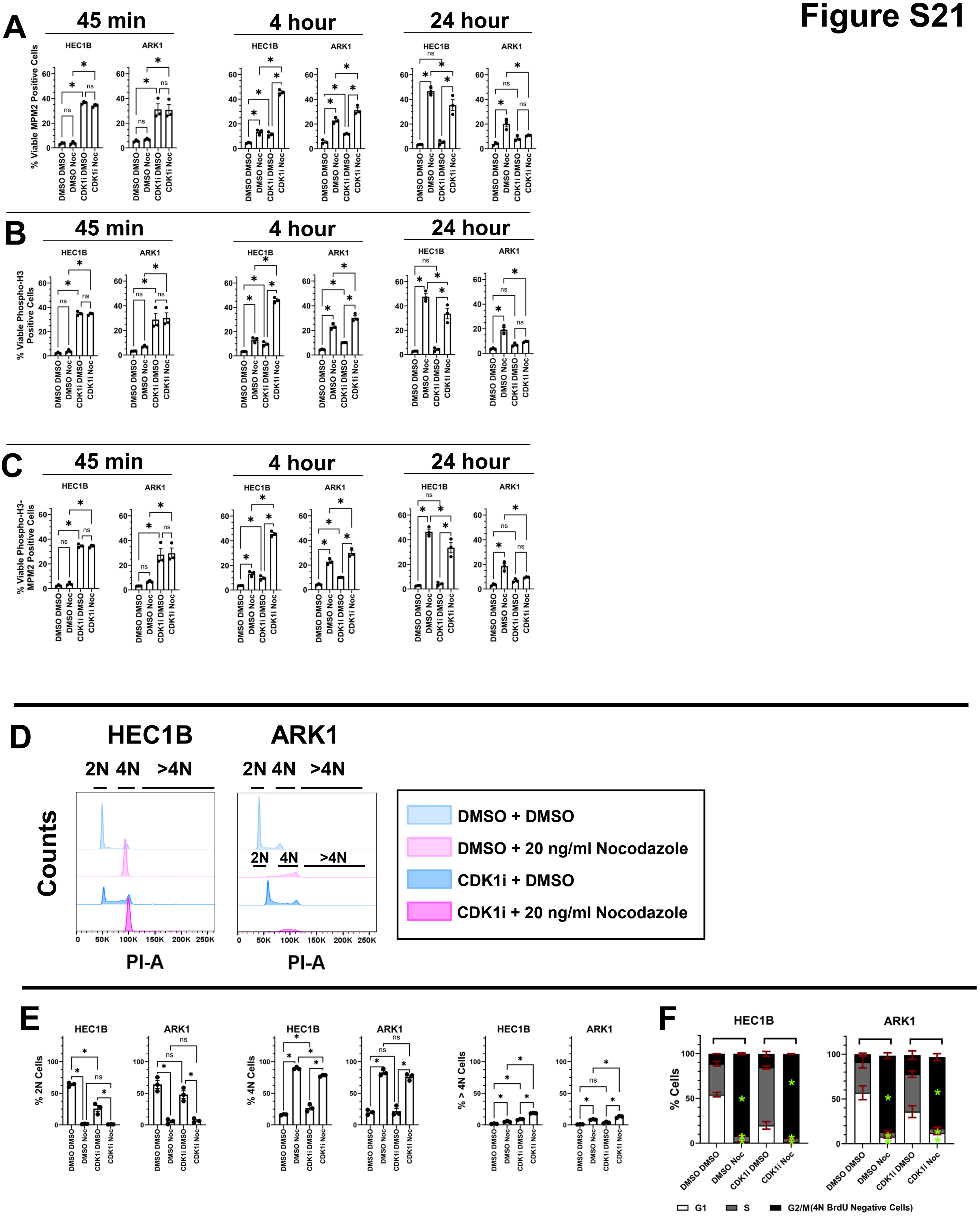
A
45 min
4 hour
24 hour
HEC1B
ARK1
% Viable MPM2 Positive Cells
B
% Viable Phospho-H3 Positive Cells
C
% Viable Phospho-H3-MPM2 Positive Cells
DMSO DMSO
DMSO Noc
CDK1i DMSO
CDK1i Noc
D
2N 4N >4N
Counts
PI-A
DMSO + DMSO
DMSO + 20 ng/ml Nocodazole
CDK1i + DMSO
CDK1i + 20 ng/ml Nocodazole
E
% 2N Cells
% 4N Cells
% > 4N Cells
F
% Cells
G1
S
G2/M(4N BrdU Negative Cells)

Figure S21. Additional statistical comparisons and cell cycle analysis for CDK1 inhibitor-20ng/mL nocodazole data corresponding to Figures S20A, S20B, and S20C. A, B, and C) The data shown here is the same data analyzed in Figures S20A, S20B, and S20C but presented in a different way to allow for different statistical comparisons to be made. In those experiments, HEC1B and ARK1 cells were treated with vehicle (DMSO) or the CDK1 inhibitor (CDK1i) Ro-3306 for 16 hours, washed, and then treated with vehicle (DMSO) or 20ng/mL nocodazole (Noc). Cells were harvested at 45 minutes (min), 4 hours, and 24 hours post-release and analyzed for different markers by flow cytometry. In the line graphs in the previous Figures S20A, S20B, and S20C, the average percentage of viable cells positive for each marker at each timepoint was shown for one drug combination with timepoints on the X-axis and percent positive cells on the Y-axis. This allowed only for comparisons between the three timepoints for a single drug combination in a model. Here to instead allow for statistical comparisons between each of the four drug treatments in one cell line at one timepoint, we are showing the data as bar graphs with the four drug treatments on the X-axis and the percentage of cells positive for the marker on the Y-axis. Panel A shows %Viable MPM2 positive cells and corresponds to Figure S20A, Panel B shows %Viable histone H3 phosphorylated on serine 10 (Phospho-H3) positive cells and corresponds to Figure S20B, and Panel C shows %Viable Phospho-H3/MPM2 double positive cells and corresponds to Figure S20C. There is one bar graph for each cell line at each timepoint. The bars in these graphs represent the average percentage of cells positive for the marker from the three independent replicates analyzed in the previous Figures. Error bars represent standard error of the mean. Brackets demonstrate statistical comparisons between different treatments at each timepoint, and statistical significance was determined by ordinary one-way ANOVA with Šídák's multiple comparisons test. *=p<0.05 and ns=not significant. Timepoints are indicated above the bar graphs. **D, E, and F)** HEC1B and ARK1 cells were treated with vehicle (DMSO) or CDK1i for 16 hours, washed, and then treated with vehicle (DMSO) or 20ng/mL nocodazole (Noc) for 24 hours. One hour prior to harvest, cells were pulsed with bromodeoxyuridine (BrdU). Cells were then harvested, stained for BrdU and Propidium Iodide (PI), and analyzed on a flow cytometer. The experiment was repeated three times. Representative PI profile plots are shown in Panel **D** from one experiment for each cell line with each drug treatment. The color code to the treatments is on the right. Bars/labels denoting peaks for 2N, 4N, and greater than 4N (>4N) DNA content cells are shown on the top of each stack and also in the middle for ARK1 cells. The data was analyzed in two ways including the PI data alone for DNA content shown in Panel E and the combined PI/BrdU data to include S phase cells shown in Panel F. In panel **E** the PI data was analyzed alone for DNA content, and the percentage of cells with 2N, 4N, or >4N DNA content is represented in the bar graphs. Bars represent the average from the three experiments while error bars represent standard error of the mean. *=p<0.05 and ns=not significant compared to the treatment indicated by the bracket over the bars by an ordinary one-way ANOVA with Šídák's multiple comparisons test. Please see Figure S8 for a representative gating strategy. In Panel **F**, the combined PI/BrdU data analysis is represented. Shown are bar graphs with bars representing the percent of cells in each different cell cycle phase from the three independent replicates and error bars represent standard error of the mean. G1 represents 2N DNA content cells that are BrdU negative, S represents BrdU positive cells, and G2/M represents 4N DNA content cells that are BrdU negative. *=p<0.05 with comparisons indicated by bracket for the specific cell cycle phase by an ordinary two-way ANOVA with Šídák's multiple comparisons test. If there is no *, then the comparison was not significant. The color code for the cell cycle phase is below one of the graphs. Please see Figure S4 for a representative gating strategy.
