# Supplementary material for: Functional Profiling of p53 and RB Cell Cycle Regulatory Proficiency Suggests Mechanism-Driven Molecular Stratification in Endometrial Carcinoma
Source: Cancer Res Commun. 2025 Apr 30;5(4):719–42. doi: 10.1158/2767-9764.CRC-24-0028 (PMC12042793; doi:10.1158/2767-9764.CRC-24-0028)
Supplement: Figure S22 — Supplementary Figure S22 [file crc-24-0028_figure_s22_suppsf22.pdf]

Figure S22

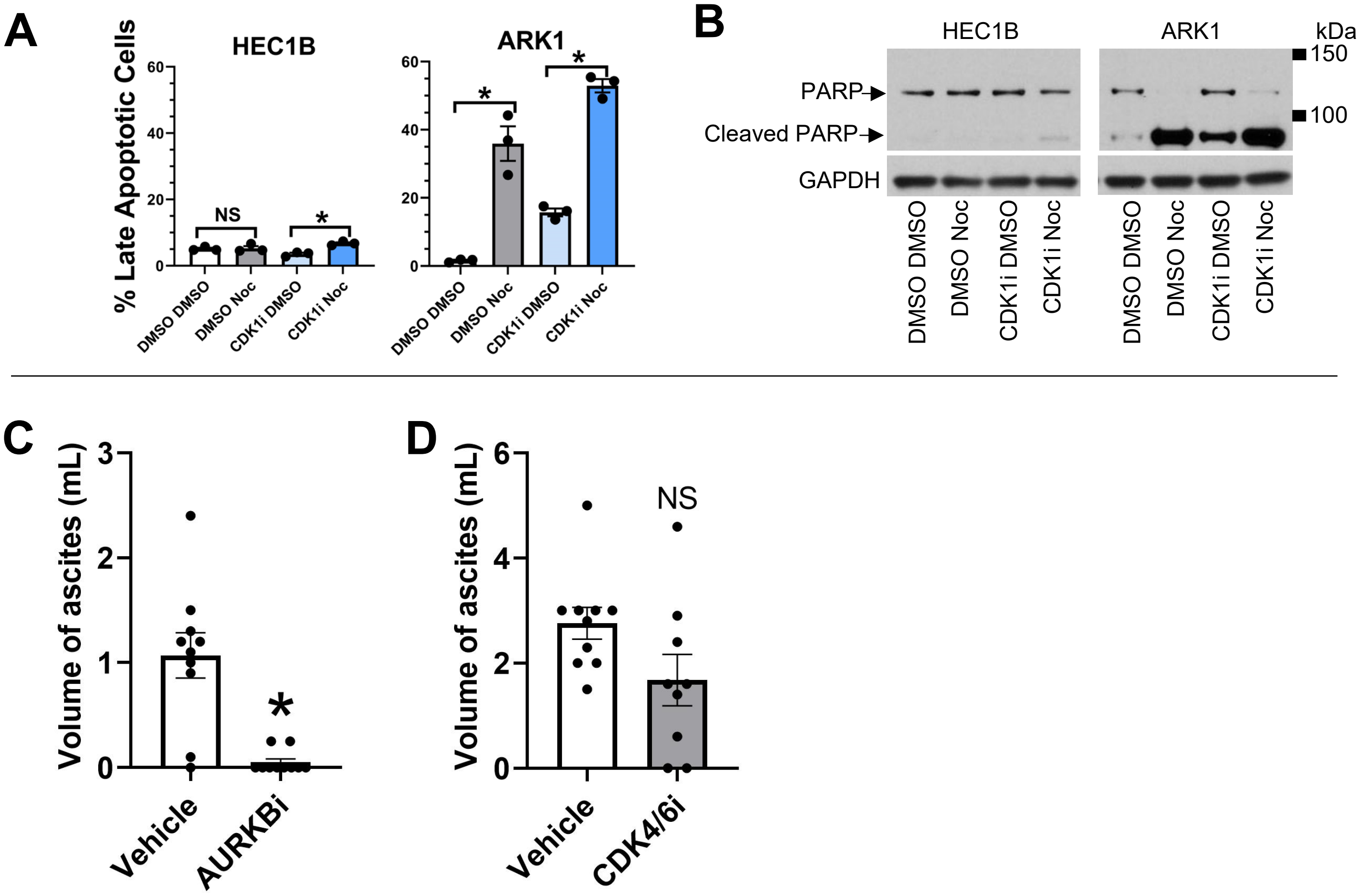

Figure S22. Apoptosis analysis for 20ng/mL nocodazole drug combinations, and ascites volumes corresponding to animal studies in Figure 6. A) HEC1B and ARK1 cells were treated with vehicle (DMSO) or the CDK1 inhibitor (CDK1i) Ro-3306 for 16 hours, washed, and then treated with vehicle (DMSO) or 20ng/mL nocodazole (Noc) for 24 hours. Cells were harvested at the appropriate timepoint and then immediately co-stained for Zombie NIR viability dye and Apotracker Green. Cells were then analyzed by flow cytometry, and the percentage of late apoptotic cells (Zombie viability dye and Apotracker Green double positive) was quantified. The bar graphs show the average percent of cells for each of the four treatments with the bars representing the average of three independent experiments with error bars representing standard error of the mean. For comparisons indicated by brackets over the treatment groups being compared, *=p<0.05 and NS=not significant by an ordinary one-way ANOVA with Šídák's multiple comparisons test. **B)** HEC1B and ARK1 cells were treated with vehicle (DMSO) or CDK1i for 16 hours, washed, and then treated with media containing vehicle (DMSO) or 20ng/mL Noc for 24 hours. Cells were then analyzed for apoptosis by western blot. Protein lysates were prepared from the variously treated cells, the same amount of protein for each cell line for each treatment was loaded into a gel to allow for comparison of markers between cell lines, and then membranes were analyzed by western blot. Membranes were first probed for PARP and cleaved PARP indicated by labels on the left, and then stripped and re-probed for GAPDH as a loading control. The cleaved PARP/PARP images shown are from the same exposure and can be compared for protein levels. **C and D)** Ascites volumes are shown here for the *in vivo* studies in Figure 6. **C)** The volume of ascites at the end of the ARK1 Aurora kinase B inhibitor (AURKBi) Barasertib experiment in Figures 6B, 6C, and 6D was measured from each animal. The bar graph shown here represents the remaining ascites volume for the 10 animals in either the vehicle- or AURKBi-treated groups. Error bars represent standard error of the mean. Significance was calculated by an unpaired t-test comparing AURKBi to vehicle, and *=p<0.05. **D)** The volume of ascites at the end of the HEC1B CDK4/6 inhibitor (CDK4/6i) Abemaciclib experiment in Figures 6E, 6F, and 6G was measured from each animal that could be assessed. The bar graph shown here represents the remaining ascites volume for the 10 animals assessed in the vehicle- or the nine animals assessed in the CDK4/6i-treated groups. Error bars represent standard error of the mean. Significance was calculated by an unpaired t-test comparing CDK4/6i to vehicle, and NS=not significant.
